# Supplementary material for: Factors associated with the time to return negative RT-PCR from COVID-19 in paediatric patients: a retrospective cohort study
Source: BMJ Open. 2021 Oct 22;11(10):e052609. doi: 10.1136/bmjopen-2021-052609 (PMC8557243; doi:10.1136/bmjopen-2021-052609)
Supplement: Supplementary data [file bmjopen-2021-052609supp001.pdf]

# Title: Factors associated with the time to return negative RT-PCR from COVID-19 in pediatric patients: a retrospective cohort study

## Authors and Institutions:

Jianbo Shao[a, †], Hong Xu[b, †], Zhixi Liu[c, †], Xiaohua Ying[c, †], Hua Xu[d], Xianfeng Wang[e], Jinmiao Lu[f], Yidie Huang[f], Guangfei Wang[f], Yanling He[g], Jie Chen[h], Shuli Ma[i], Shangrong Zou[j], Yuxia Cui[k], Ruijie Chen[l], Jin Lu[m], Xuyuan Li[n], Zhiping Li[f, *], Guoying Huang [f, *], Weibing Wang [c, *]

[a]Department of Radiological Medicine, Wuhan Children's Hospital (Wuhan Maternal and Child Healthcare Hospital), Tongji Medical College, Huazhong University of Science & Technology, Wuhan 430016, China; and [b]Department of Nephrology, National Children's Medical Center, Children's Hospital of Fudan University, Shanghai 201102, China; and [c]School of Public Health, Fudan University, Shanghai 200032, China; and [d]Department of Pharmacy, Wuhan Children's Hospital (Wuhan Maternal and Child Healthcare Hospital), Tongji Medical College, Huazhong University of Science & Technology, Wuhan 430016, China; and [e]Department of Pediatrics, The Third People's Hospital of Shenzhen, Shenzhen 518020, China; and [f]Department of Clinical Pharmacy, National Children's Medical Center, Children's Hospital of Fudan University, Shanghai 201102, China; and [g]Department of Pharmacy, Guangzhou Women and Children's Medical Center, Guangzhou 510623, China; and [h]Department of Pharmacy, The Third People's Hospital of Kunming, Kunming 650000, China; and [i]Department of Pharmacy, Children's Hospital Affiliated to Zhengzhou University, Zhengzhou 450000, China; and [j]Department of Pharmacy, Guangzhou Eighth People's Hospital, Guangzhou 510060, China; and [k]Department of Pediatrics, Guizhou Provincial People's Hospital, Guiyang 550002, China; and [l]Wenzhou Municipal Key Laboratory of Paediatric Pharmacy, Department of Pharmacy, The Second

Affiliated Hospital and Yuying Children's Hospital of Wenzhou Medical University, Wenzhou 325027, China; and [m]Department of Pharmacy, The First Affiliated Hospital of USTC, Division of Life Sciences and Medicine, University of Science and Technology of China, Anhui 230001, China; and [n]Department of Biology, University of California, Santa Barbara 93101, California, the United States

[†] Jianbo Shao, Hong Xu, Zhixi Liu, Xiaohua Ying contributed equally to this manuscript

[*] Zhiping Li, Guoying Huang, Weibing Wang contributed equally to corresponding work of this manuscript

## Corresponding author:

Dr. Zhiping Li, PhD, Department of Clinical Pharmacy, National Children's Medical Center, Children's Hospital of Fudan University, Shanghai 201102, China

Email: zpli@fudan.edu.cn

Dr. Guoying Huang*, MD, PhD, Centre of Cardiovascular, National Children's Medical Center, Children's Hospital of Fudan University, Shanghai 201102, China

Email: gyhuang@shmu.edu.cn

Dr. Weibing Wang, PhD, Department of Epidemiology, School of Public Health, Fudan University, 138 Yi Xue Yuan Road, Shanghai 200032, China, 86-21-54237811

Email: wwb@fudan.edu.cn

## Contents:

Supplementary Table 1. Definitions of clinical types for COVID-19 pediatric patients
Supplementary Figure 1. The spatial distribution of the 12 COVID-19 tertiary hospitals
Supplementary Figure 2. Comparison of clinical indicators stratified by age pre-therapy and post-treatment

**Supplementary Table 1. Definitions of clinical types for COVID-19 pediatric patients**

| Classification of clinical types | Based on the clinical characteristics of confirmed COVID-19 pediatric cases |
|---|---|
| Asymptomatic disease | • No clinical symptoms or signs<br>• Normal chest imaging examination<br>• Positive RT-PCR test for SARS-CoV-2 or positive result for retrospectively diagnosed sero-specific antibody |
| Mild disease | • Acute upper respiratory tract infection (*e.g.*, fever, fatigue, myalgia, cough, sore throat, runny nose and sneezing, *etc.*)<br>• Pharyngeal congestion<br>• No positive signs of lungs<br>• Some may have no fever, or only other gastrointestinal symptoms (*e.g.*, nausea, vomiting, abdominal pain and diarrhea) |
| Moderate disease | • Pneumonia<br>• Clinical symptoms such as fever, cough<br>• Wheezing, dry and (or) moist rales were heard in the lungs<br>• Sub-clinical: some without clinical symptoms or signs but with abnormal pulmonary lesions |
| Severe disease | • Early symptoms of respiratory tract (*e.g.*, fever and cough, *etc*); may be accompanied by digestive tract symptoms (e.g., diarrhea, *etc*)<br>• Progression of disease within a week or so, with dyspnea, central cyanosis, hypoxia, pulse oximetry <0.92 |
| Critical disease | • ARDS<br>• Respiratory failure<br>• Multiple organ dysfunction (*e.g.*, shock, encephalopathy, myocardial injury or failure, coagulation dysfunction and acute kidney injury) |

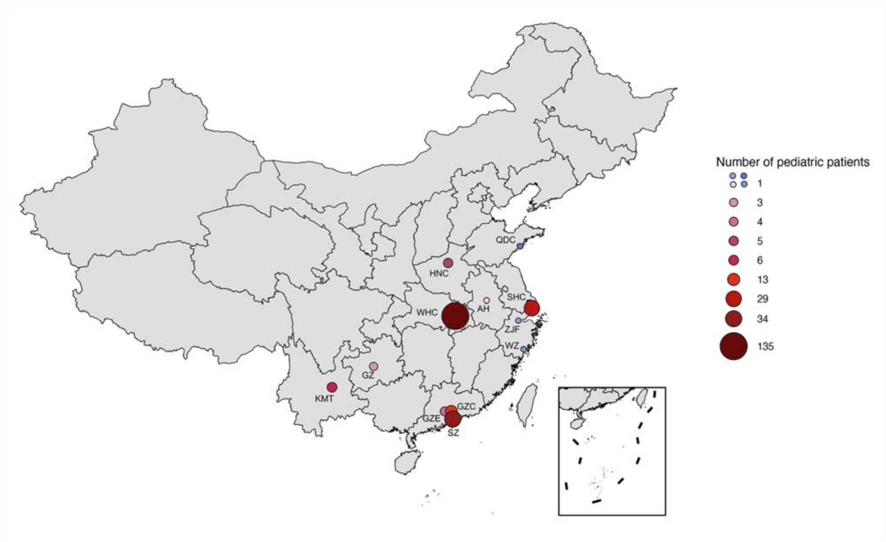


**Supplementary Figure 1. The spatial distribution of the 12 COVID-19 tertiary hospitals**

The number of patients enrolled in the study is varied by the size of the points and the hospitals varied by the color of the points. Abbreviations: WHC: Wuhan Children's Hospital (Wuhan Maternal and Child Healthcare Hospital), Tongji Medical College, Huazhong University of Science & Technology; SZ: The Third People’s Hospital of Shenzhen; SHC: National Children’s Medical Center, Children’s Hospital of Fudan University; GZC: Guangzhou Women and Children’s Medical Center; KMT: The Third People’s Hospital of Kunming; HNC: Children’s Hospital Affiliated to Zhengzhou University; GZE: Guangzhou Eighth People’s Hospital; GZ: Guizhou Provincial People’s Hospital; QDC: Qingdao Children’s Hospital; AH: The First Affiliated Hospital of USTC, Division of Life Sciences and Medicine, University of Science and Technology of China; ZJF: The First Hospital of Zhejiang University and WZ: The Second Affiliated Hospital and Yuying Children’s Hospital of Wenzhou Medical University.

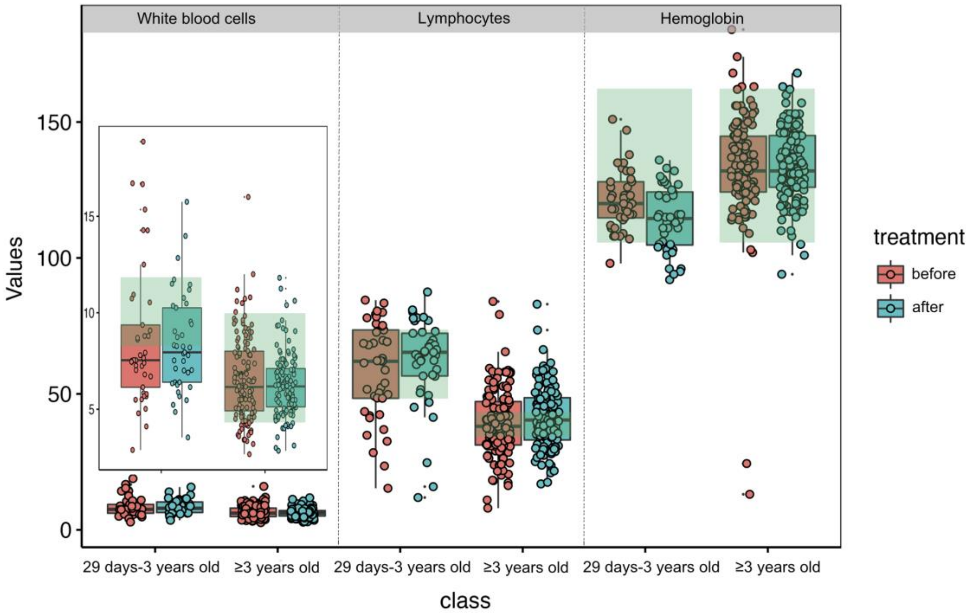


**Supplementary Figure 2. Comparison of clinical indicators stratified by age pre-therapy and post-treatment**

The red and green boxes represent the clinical indicator values before and after treatment, respectively. The scattered points on each box represent the values of each clinical indicator for each patient. The shaded green rectangle represents the normal reference range for an indicator for that age group. The middle line of the box represents the medium value of the group. The height of the box represents the interquartile range (IQR) of each group.
