# Supplementary material for: Factors associated with the time to return negative RT-PCR from COVID-19 in paediatric patients: a retrospective cohort study
Source: BMJ Open. 2021 Oct 22;11(10):e052609. doi: 10.1136/bmjopen-2021-052609 (PMC8557243; doi:10.1136/bmjopen-2021-052609)
Supplement: Author's manuscript [file bmjopen-2021-052609.draft_revisions.pdf]

BMJ Open is committed to open peer review. As part of this commitment we make the peer review history of every article we publish publicly available.

When an article is published we post the peer reviewers' comments and the authors' responses online. We also post the versions of the paper that were used during peer review. These are the versions that the peer review comments apply to.

The versions of the paper that follow are the versions that were submitted during the peer review process. They are not the versions of record or the final published versions. They should not be cited or distributed as the published version of this manuscript.

BMJ Open is an open access journal and the full, final, typeset and author-corrected version of record of the manuscript is available on our site with no access controls, subscription charges or pay-per-view fees (http://bmjopen.bmj.com).

If you have any questions on BMJ Open's open peer review process please email info.bmjopen@bmj.com

# Factors associated with the time to turn negative RT-PCR from COVID-19 in pediatric patients


| | |
|---:|---|
| Journal: | *BMJ Open* |
| Manuscript ID | bmjopen-2021-052609 |
| Article Type: | Original research |
| Date Submitted by the Author: | 20-Apr-2021 |



| | |
|---:|---|
| Complete List of Authors: | Shao, Jianbo; Huazhong University of Science and Technology Tongji Medical College Affiliated Wuhan Children's Hospital<br>Xu, Hong; Children's Hospital of Fudan University, Pediatric Nephrology<br>Liu, Zhixi; Fudan University School of Public Health<br>YING, Xiaohua; Fudan University School of Public Health<br>Xu, Hua; Huazhong University of Science and Technology Tongji Medical College Affiliated Wuhan Children's Hospital, Department of Pharmacy<br>Wang, Xianfeng; The Third People's Hospital of Shenzhen<br>Lu, Jinmiao; Children's Hospital of Fudan University, Department of Clinical Pharmacy<br>Huang, Yidie; Children's Hospital of Fudan University, Department of Clinical Pharmacy<br>Wang, Guangfei; Children's Hospital of Fudan University, Department of Clinical Pharmacy<br>He, Yanling; Guangzhou Women and Children's Medical Center, Department of Pharmacy<br>Chen, Jie; Dali University, Affiliated Sixth People's Hospital<br>Ma, Shuli; Zhengzhou University, Affiliated Children's Hospital<br>Zou, Shangrong; Guangzhou Eighth People's Hospital<br>Cui, Yuxia; Guizhou Provincial People's Hospital<br>Chen, Ruijie; Yuying Children's Hospital of Wenzhou Medical College<br>Lu, Jin; University of Science and Technology of China<br>Li, Xuyuan; University of California Santa Barbara<br>Li, Zhiping; Children's Hospital of Fudan University, Department of Clinical Pharmacy<br>Huang, Guoying; Children's Hospital of Fudan University, Department of Clinical Pharmacy<br>Wang, Weibing; Fudan University School of Public Health, Department of Pediatrics |



| | |
|---:|---|
| Keywords: | COVID-19, Epidemiology < INFECTIOUS DISEASES, PAEDIATRICS |
| | |


SCHOLARONE™
Manuscripts

*I, the Submitting Author has the right to grant and does grant on behalf of all authors of the Work (as defined in the below author licence), an exclusive licence and/or a non-exclusive licence for contributions from authors who are: i) UK Crown employees; ii) where BMJ has agreed a CC-BY licence shall apply, and/or iii) in accordance with the terms applicable for US Federal Government officers or employees acting as part of their official duties; on a worldwide, perpetual, irrevocable, royalty-free basis to BMJ Publishing Group Ltd ("BMJ") its licensees and where the relevant Journal is co-owned by BMJ to the co-owners of the Journal, to publish the Work in this journal and any other BMJ products and to exploit all rights, as set out in our licence.*

*The Submitting Author accepts and understands that any supply made under these terms is made by BMJ to the Submitting Author unless you are acting as an employee on behalf of your employer or a postgraduate student of an affiliated institution which is paying any applicable article publishing charge ("APC") for Open Access articles. Where the Submitting Author wishes to make the Work available on an Open Access basis (and intends to pay the relevant APC), the terms of reuse of such Open Access shall be governed by a Creative Commons licence – details of these licences and which Creative Commons licence will apply to this Work are set out in our licence referred to above.*

*Other than as permitted in any relevant BMJ Author's Self Archiving Policies, I confirm this Work has not been accepted for publication elsewhere, is not being considered for publication elsewhere and does not duplicate material already published. I confirm all authors consent to publication of this Work and authorise the granting of this licence.*

For peer review only

# Title: Factors associated with the time to turn negative RT-PCR from COVID-19 in pediatric patients

## Authors and Institutions:

Jianbo Shao[a, †], Hong Xu[b, †], Zhixi Liu[c, †], Xiaohua Ying[c, †], Hua Xu[d], Xianfeng Wang[e], Jinmiao Lu[f], Yidie Huang[f], Guangfei Wang[f], Yanling He[g], Jie Chen[h], Shuli Ma[i], Shangrong Zou[j], Yuxia Cui[k], Ruijie Chen[l], Jin Lu[m], Xuyuan Li[n], Zhiping Li[f, *], Guoying Huang [f, *], Weibing Wang [c, *]

[a]Department of Radiological Medicine, Wuhan Children's Hospital (Wuhan Maternal and Child Healthcare Hospital), Tongji Medical College, Huazhong University of Science & Technology, Wuhan 430016, China; and [b]Department of Nephrology, National Children's Medical Center, Children's Hospital of Fudan University, Shanghai 201102, China; and [c]School of Public Health, Fudan University, Shanghai 200032, China; and [d]Department of Pharmacy, Wuhan Children's Hospital (Wuhan Maternal and Child Healthcare Hospital), Tongji Medical College, Huazhong University of Science & Technology, Wuhan 430016, China; and [e]Department of Pediatrics, The Third People's Hospital of Shenzhen, Shenzhen 518020, China; and [f]Department of Clinical Pharmacy, National Children's Medical Center, Children's Hospital of Fudan University, Shanghai 201102, China; and [g]Department of Pharmacy, Guangzhou Women and Children's Medical Center, Guangzhou 510623, China; and [h]Department of Pharmacy, The Third People's Hospital of Kunming, Kunming 650000, China; and [i]Department of Pharmacy, Children's Hospital Affiliated to Zhengzhou University, Zhengzhou 450000, China; and [j]Department of Pharmacy, Guangzhou Eighth People's Hospital, Guangzhou 510060, China; and [k]Department of Pediatrics, Guizhou Provincial People's Hospital, Guiyang 550002, China; and [l]Wenzhou Municipal Key Laboratory of Paediatric Pharmacy, Department of Pharmacy, The Second Affiliated Hospital and Yuying Children's Hospital of Wenzhou Medical University, Wenzhou 325027, China; and [m]Department of Pharmacy,



For peer review only - http://bmjopen.bmj.com/site/about/guidelines.xhtml

The First Affiliated Hospital of USTC, Division of Life Sciences and Medicine, University of Science and Technology of China, Anhui 230001, China; and [n]Department of Biology, University of California, Santa Barbara 93101, California, the United States

† Jianbo Shao, Hong Xu, Zhixi Liu, Xiaohua Ying contributed equally to this manuscript

* Zhiping Li, Guoying Huang, Weibing Wang contributed equally to corresponding work of this manuscript

## Corresponding author:

Dr. Zhiping Li, PhD, Department of Clinical Pharmacy, National Children's Medical Center, Children's Hospital of Fudan University, Shanghai 201102, China

Email: zpli@fudan.edu.cn

Dr. Guoying Huang*, MD, PhD, Centre of Cardiovascular, National Children's Medical Center, Children's Hospital of Fudan University, Shanghai 201102, China

Email: gyhuang@shmu.edu.cn

Dr. Weibing Wang, PhD, Department of Epidemiology, School of Public Health, Fudan University, 138 Yi Xue Yuan Road, Shanghai 200032, China, 86-21-54237811

Email: wwb@fudan.edu.cn

Word count: 3105

For peer review only

# Abstract

**Objective** This study aimed to describe the epidemiological and clinical features and potential factors related to the time to turn negative RT-PCR in discharged pediatric patients with COVID-19.

**Design** Retrospective cohort study

**Methods** This retrospective study examined 233 pediatric cases confirmed COVID-19 admitted to 12 tertiary hospitals in China from January 1, 2020 to April 17, 2020. We retrieved the electronic medical records of these cases, including the patient epidemiological characteristics, laboratory results, and clinical information. Statistical descriptions were conducted by Fisher exact test or Chi-square test. Furthermore, we used Cox proportional hazards regression models to explore factors related to the time to turn negative RT-PCR.

**Results** The median age of the cohort was 7·50 (IQR: 2·92,12·17) years and 133 (57·1%) patients were male. 42 (18.0%) patients were evaluated as asymptomatic, while 162 (69.5%) and 25 (10.7%) patients were classified as mild or moderate, respectively. In Cox regression analysis, longer time to negative RT-PCR was associated with the presence of confirmed patients in the family (Hazard Ratio (HR) [95%CI]: 0.56 [0.41–0.79]). Pediatric patients with gastrointestinal symptoms had a longer time to turn negative (HR [95%CI]: 0.33 [0.14–0.78]). Usage of combination of Chinese medicine and antiviral drug isn't more conducive to turn negative than only use antiviral drug (HR [95%CI]: 0.85[0.64-1.13]).

**Conclusions** The mode of transmission might be a critical factor determining the disease severity of COVID-19. Patients with gastrointestinal symptoms, complications or infected members in families may prolong rehabilitation. However, no significant favorable effects from TCM when the patients have received antiviral treatment.


**Keywords** SARS-CoV-2; COVID-19; infectious disease; pediatrics

For peer review only



For peer review only - http://bmjopen.bmj.com/site/about/guidelines.xhtml

## Strengths and limitations of this study

- This study enrolled all pediatric COVID-19 patients (n = 233) discharged from 12 tertiary hospitals across 11 cities and found pediatric patients with gastrointestinal symptoms (*e.g.*, emesis) had a longer time to turn negative.
- We determined the in-family transmission is the main infection route for pediatric COVID-19 cases.
- Transmission mode may be key factors determining the clinical course of COVID-19.
- This study was limited to a small number of pediatric COVID-19 patients, given that the prevalence of COVID-19 pediatric cases is relatively low compared with adults population.

For peer review only

# Introduction

Coronavirus disease 2019 is an emerging infectious disease with high transmissibility. As of June 29, 2020, the cumulative number of cases worldwide has reached 10,021,401, with 85,204 of those cases in China[1]. On March 11, 2020, the World Health Organization (WHO) further announced the epidemic of COVID-19 (Coronavirus disease 2019) would be classified as a global pandemic[2]. According to the data released by the WHO[3], pneumonia is the leading cause of death among children worldwide and the second among children under five in China[4]. Pediatric cases were reported during the epidemics of SARS-CoV (severe acute respiratory syndrome coronavirus)[5 6]. In pediatric SARS patients, the clinical symptoms of teenagers were similar to those of adults[7]. Furthermore, they maintained stable conditions throughout the disease course and had good prognoses[8]. Another study demonstrated that pediatric cases accounted for only 2% of all Middle East Respiratory Syndrome cases[9]. One previous study that enrolled 36 COVID-19 pediatric cases concluded that the close contacts with family members might be difficult to avoid in daily life[10].

The low proportion of pediatric patients among COVID-19 cases has perplexed clinicians, epidemiologists, and scientists. Current understanding regarding the duration of positive RT-PCR to negative for COVID-19 is limited, and this knowledge gap is particularly notable among children. Therefore, we aimed to explore and analyze the clinical and epidemiological characteristics of 233 discharged pediatric patients to reveal corresponding information for the prevention and treatment of pediatric COVID-19 patients.

# Materials and Methods

## Study population

This multi-center retrospective study was conducted in 12 tertiary hospitals in 11 Chinese cities. We collected information about the discharged pediatric COVID-19 patients treated in these hospitals from January 1, 2020 to



For peer review only - http://bmjopen.bmj.com/site/about/guidelines.xhtml

April 17, 2020. The pediatric patients with confirmed COVID-19 were diagnosed based on guidelines issued by the National Health Commission of the People's Republic of China[11]. This study was approved by the ethics committee of the National Children's Medical Center, Children's Hospital of Fudan University, and followed the Declaration of Helsinki. Written consent was obtained from the guardians of the patients.

## Procedure

Relevant medical information was collected from electronic medical records and reviewed by trained doctors to ensure the quality of the medical data. Retrieved information included the demographic information, COVID-19 contact history (e.g., history of travel to or residence in the COVID-19 epidemic areas or history of contact with SARS-CoV-2-infected individuals), complications, laboratory tests results, chest computed tomography and B-scan ultrasonography results. Laboratory tests included routine tests and examinations for lung, liver, myocardial, and kidney function, such as measurements of the aspartate amino-transferase (AST), alanine amino-transferase (ALT), lactate dehydrogenase (LDH), α-hydroxybutyrate dehydrogenase (α-HBDH) and the erythrocyte sedimentation rate (ESR).

The preliminary diagnosis of suspected COVID-19 was based on clinical symptoms and potential exposure via travel to COVID-19 epidemic areas or close contact with a confirmed case. The diagnosis was subsequently confirmed based on results from real-time reverse transcriptase-polymerase chain reaction (RT-PCR) tests for SARS-CoV-2 (severe acute respiratory syndrome coronavirus 2) on nasal or pharyngeal swab specimens from all suspected patients diagnosed by the previous step. RT-PCR is recommended by the Center for Disease Control and Prevention (CDC)[11] and WHO interim guidance as a solid method for confirming COVID-19 diagnosis[12]. RT-PCR testing was performed to detect infection with other viruses (i.e., adenovirus, influenza virus, parainfluenza virus, and respiratory syncytial virus) and performed sputum culture to test for potential infections with bacteria,



For peer review only - http://bmjopen.bmj.com/site/about/guidelines.xhtml

including *Legionella pneumophila*, *Mycoplasma pneumoniae*, and *Chlamydia pneumoniae*. We evaluated the clinical type of each admitted pediatric COVID-19 patient according to the recommendations issued by the pediatrics branch of the Chinese Medical Association[13]. Patients were described as having one of four clinical types of COVID-19, i.e., asymptomatic, mild, moderate and severe disease, based on their RT-PCR results, clinical symptoms and radiological results (online supplementary Table 1)[14 15]. Once admitted, these pediatric patients were given available antiviral treatments (e.g., Ribavirin aerosol, Oseltamivir granule, etc.) and oxygen if necessary (blood oxygen saturation <0.92). The discharge criteria were that the patient must have obtained two consecutive negative RT-PCR test results.

## Statistical analysis

Categorical variables are presented as number and frequency rate using the Fisher exact test or Chi-square test. Continuous variables with non-normal distribution are presented as the median and interquartile range (IQR) and were compared using the Kruskal-Wallis Rank Sum Test. In addition, we analyzed the first and last laboratory test results to examine the effect of treatment during hospitalization. These two test results were compared by a paired Student's t-test or Wilcoxon's signed rank test.

Cox proportional hazards regression models were used to estimate hazard ratios (HR) and corresponding 95% confidence intervals (CI) to explore associations between risk factors and the number of days to turn negative RT-PCR for the 233 pediatric COVID-19 cases. In Cox regression models, the interpretation of results is that the endpoint event we observed was negative RT-PCR (benign event); if the HR value of a variable is less than 1, the variable is not conducive to the occurrence of the outcome of negative RT-PCR, and there will be a longer day to turn negative, and vice versa. Time to turn negative was defined as the time from positive RT-PCR results to negative RT-PCR results for SARS-CoV-2 during the hospitalization. During the analysis, sample size varied



For peer review only - http://bmjopen.bmj.com/site/about/guidelines.xhtml

because of missing data; the sample sizes for each analysis are listed in Table 1. The variables assessed in the statistical analysis were contact history, clinical symptoms, and clinical indicators. Univariate and multivariate analyses adjusted for sex, age, and weight were carried out by Cox proportional hazard ratio models to examine significant associations. These analyses conducted by R-packages 'dplyr', a data manipulation package, will provide any information about factors that contribute to a longer duration of positive RT-PCR to negative; 'survival', 'survminer' helped to determine the risk factors that contributed to a longer time of positive RT-PCR.

All analyses were conducted on the subjects who lacked missing data, and only differences with two-sided α and a p-value less than 0.05 was considered statistically significant. All analyses were conducted with the use of R software version 3.6.2.

### Patient and Public Involvement

Patients and/or the public were not involved in the design, or conduct, or reporting or dissemination plans of this research.

## Results

### Demographic and clinical characteristics

From January 1, 2020 and April 17, 2020, 233 pediatric COVID-19 cases were discharged from the 12 study hospitals (online supplementary Figure 1). These cases with a median age of 7.50 (IQR: 2.92,12.17) years and 133 (57.1%) male patients. Most patients (n = 199 [85.4%]) had a COVID-19-contact history, including contact with people from Wuhan/Hubei or travel to Wuhan/Hubei. 59 (26.5%) of the pediatric patients were the first SARS-CoV-2-infected individual of their family. Additionally, 40 (17.2%) patients had a simultaneous infection with an additional virus, and 79 (33.9%) patients had a simultaneous bacterial infection. None of the pediatric patients in our study were found to be at risk of death due to COVID-19 during hospitalization (Table 1).



For peer review only - http://bmjopen.bmj.com/site/about/guidelines.xhtml

For peer review only

**Table 1. Clinical Features of 233 Pediatric Patients Infected with SARS-CoV-2**

| Clinical factors | | All patients N (%) 233(100.0) | Asymptomatic N (%) 42(18.0) | Mild[c] N (%) 162(69.5) | Moderate Severe N (%) 29(12.4) | p[e] |
|---|---|---|---|---|---|---|
| Sex | Male | 133(57.1) | 29(69.0) | 88(54.3) | 16(55.2) | 0.223 |
| | Female | 100(42.9) | 13(31.0) | 74(45.7) | 13(44.8) | |
| Age, y | ≤ 5 | 84(36.1) | 6(14.3) | 61(37.7) | 17(58.6) | <0.001 |
| | > 5 | 149(63.9) | 36(85.7) | 101(62.3) | 12(41.4) | |
| Age, median (IQR), y | | 7.50(2.92,12.17) | 9.37(7.00,12.15) | 7.92(2.85,12.29) | 4.00(0.85,8.92) | 0.010[f] |
| Contact history of epidemic area | Yes | 199(85.4) | 41(97.6) | 132(81.5) | 26(89.7) | 0.015[g] |
| Number of infected family member | 0 | 59(26.5) | 4(9.8) | 46(29.5) | 9(34.6) | 0.185 |
| | 1 | 67(30.0) | 16(39.0) | 43(27.6) | 8(30.8) | |
| | 2 | 54(24.2) | 10(24.4) | 39(25.0) | 5(19.2) | |
| | ≥ 3 | 43(19.3) | 11(26.8) | 28(17.9) | 4(15.4) | |
| Combined with virus infection[a] | Yes | 40(17.2) | 2(4.8) | 31(19.1) | 7(24.1) | 0.039 |
| Combined with bacterial infection[b] | Yes | 79(33.9) | 13(31.0) | 60(37.0) | 6(20.7) | 0.142 |
| Fever | Yes | 86(36.9) | 0(0.0) | 69(42.6) | 17(58.6) | <0.001 |
| Cough | Yes | 83(35.6) | 0(0.0) | 68(42.0) | 15(51.7) | <0.001 |
| Running nose | Yes | 21(9.0) | 0(0.0) | 17(10.5) | 4(13.8) | 0.027[g] |
| Diarrhea | Yes | 12(5.2) | 0(0.0) | 9(5.6) | 3(10.3) | 0.127[g] |
| Nausea | Yes | 38(16.3) | 0(0.0) | 32(19.8) | 6(20.7) | 0.001[g] |
| Coughing of phlegm | Yes | 23(9.9) | 0(0.0) | 16(9.9) | 7(24.1) | 0.002[g] |
| Pharyngitis | Yes | 13(5.6) | 0(0.0) | 10(6.2) | 3(10.3) | 0.111[g] |
| X-ray or CT test findings | Abnormal | 139(59.7) | 17(40.5) | 94(58.0) | 28(96.6) | <0.001 |
| Complications | Yes | 31(13.3) | 3(7.1) | 22(13.6) | 6(20.7) | 0.268 |
| Days in hospital, median (IQR), d | | 12.00(9.00,17.00) | 10.50(8.25,14.00) | 12.00(9.00,17.00) | 13.00(9.75,17.00) | 0.416[f] |

a: Adenovirus, influenza virus, parainfluenza virus, respiratory syncytial virus, *etc*.

b: Legionella pneumophila, Mycoplasma pneumoniae, Chlamydia pneumoniae, *etc*.

c: Mild disease: upper respiratory symptoms for a short duration or asymptomatic infection; positive RT-PCR test for SARS-CoV-2; no abnormal radiographic and septic presentation.

d: Moderate disease: mild pneumonia; symptoms such as fever, cough, fatigue, headache, and myalgia; no complications and manifestations related to severe conditions.

e: p-values indicate the difference among four different disease types in pediatric COVID-19 patients with pneumonia.

f: p-value was obtained from a Kruskal-Wallis test; [g]: p-values were obtained from Fisher exact tests.

The most common clinical symptoms of the 233 pediatric COVID-19 patients were fever (40.8%), cough (35.6%) and nausea (16.3%). The symptoms of running nose, diarrhea, and pharyngitis were also reported. All patients

For peer review only



For peer review only - http://bmjopen.bmj.com/site/about/guidelines.xhtml

underwent a chest X-ray or CT test, and the results suggest that 139 (59.7%) patients had lung abnormalities, which include 17(40.5%) of asymptomatic patients, 94 (58.0%) of mild patients, and 28 (96.6%) of moderate or severe patients. The lung abnormalities included ground-glass opacities and patchy shadows or multiple plaques in some of the lung lobes on a CT scan. The median length of the patient hospital stay was 12 days (IQR: 9–17 days). An examination of patients' complications revealed that 31 of the 233 pediatric COVID-19 patients had complications (e.g., myocardial injury and liver insufficiency, etc.), including 3 (7.1%) asymptomatic patients, 22 (13.6%) mild patients, and 6 (20.7%) moderate or severe patients.

The clinical disease was classified as asymptomatic in 42 (18.0%) patients, mild in 162 (69.5%) patients, moderate in 25 (10.7%) patients and severe in 4 (1.7%) patients. As the severity of disease increased, the median age (p = 0.010) decreased and the proportion of children under 5 years old (p < 0.001) increased. Notably, there was no significant sex difference in severity of disease (p = 0.223). When comparing between children with or without infected family members, the severity of illness exhibited no significant correlation. Among the patients with moderate or severe disease, 17 (58.6%) had fever, 15 (51.7%) had cough and 7 (24.1%) had coughing of phlegm. Only 13.8% and 10.3% had the symptoms of running nose and diarrhea, respectively. Of the mild cases, 69 (42.6%), 68 (42.0%), and 32 (19.8%) patients had fever, cough, and nausea, respectively. In contrast, none of the asymptomatic patients had any of the clinical symptoms that were common in the patients with mild or moderate disease.

## Laboratory indices at hospital admission for the 233 pediatric COVID-19 patients

Here are the results of laboratory indices obtained in the first laboratory test after hospital admission (Table 2). Of the 227 cases, fifty had leukopenia. More than half of the patients (n = 150 [65.50%]) had an abnormal lymphocyte percentage; 52 (22.70%) had lymphopenia. In addition, some patients were diagnosed with abnormal liver function,



For peer review only - http://bmjopen.bmj.com/site/about/guidelines.xhtml

For peer review only

as indicated by 69 (30.70%) patients with elevated AST levels, while 20 (8.80%) patients had elevated ALT levels and 41 (20.20%) had increased procalcitonin levels. We also detected renal damage in some patients, as indicated by elevated levels of creatinine (n = 12 [5.40%]) and urea (n = 11 [5.00%]). Some patients exhibited elevated levels of LDH (n = 36 [18.30%]) and α-HBDH (n = 13 [59.10%]). Levels of infection-related indices, such as IL-6 (35 [24.10%]), CRP (40 [19.00%]), and ESR (19 [17.00%]), were also elevated in some patients.

For peer review only

**Table 2. Laboratory Indices at Hospital Admission of Pediatric Patients with COVID-19 Pneumonia**

| Tests in study population | Reference values | No. of patients tested | Median (IQR[l]) | No. of patients with value deviation from reference (%) | |
|---|---|---|---|---|---|
| | | | | Below reference | Above reference |
| **Hematologic** | | | | | |
| White blood cells, $\times 10^9$/mL | Varied with age[j] | 227 | 6.64(5.31-8.18) | 50 (22.0) | 17 (7.5) |
| Lymphocytes, % | Varied with age[j] | 229 | 42.40(33.00-55.10) | 52 (22.7) | 98 (42.8) |
| Platelets, $\times 10^9$/L | 100-400 | 224 | 266.00(225.00-325.00) | 1 (0.4) | 20 (8.9) |
| Hemoglobin, g/L | Varied with age[j] | 224 | 129.00(119.80-138.20) | 16 (7.2) | 7 (3.2) |
| **Biochemical** | | | | | |
| AST[a], IU/L | 13-35 | 225 | 29.00(20.00-41.00) | 10 (4.4) | 69 (30.7) |
| ALT[b], IU/L | 7-40 | 227 | 15.50(11.00-24.75) | 16 (7.0) | 20 (8.8) |
| Albumin, g/L | 40-55 | 223 | 45.60(43.10-48.05) | 16 (7.2) | 3 (1.3) |
| Prealbumin, mg/L | 200-400 | 142 | 194.30(153.85-229.68) | 76 (53.5) | - |
| Urea, mmol/L | 2.8-7.6 | 218 | 3.97(3.23-5.06) | 28 (12.8) | 11 (5.0) |
| Creatinine, µmol/L | Varied with age[j] and sex | 225 | 36.60(26.80-48.90) | 8 (3.6) | 12 (5.4) |
| Cholinesterase, U/L | 5300-11300 | 40 | 8914.00(7690.00-10022.00) | - | 4 (10.0) |
| LDH[c], U/L | Varied with age[j] | 196 | 238.00(194.80-311.80) | 2 (1.0) | 36 (18.3) |
| α-HBDH[d], U/L | 72-182 | 22 | 185.50(162.50-231.50) | - | 13 (59.1) |
| Procalcitonin, ng/ml | < 0.1 | 186 | 0.05(0.03-0.08) | - | 41 (20.2) |
| **Infection-related indices** | | | | | |
| IL-6[e], pg/ml | < 7 | 145 | 3.63(2.36-6.85) | - | 35 (24.1) |
| CRP[f], mg/L | < 8 | 211 | - | - | 40 (19.0) |
| ESR[g], mm/h | Varied with age and sex[k] | 112 | 10.00(5.75-19.00) | - | 19 (17.0) |
| **Coagulation function** | | | | | |
| PT[h], s | 11-14.5 | 191 | 11.40(10.80-12.50) | 59 (30.9) | 6 (3.1) |
| APTT[i], s | 26-40 | 191 | 32.8(29.70-35.35) | 12 (6.3) | 25 (13.1) |
| D-dimer, ug/ml | 0-0.5 | 150 | 0.26(0.15-0.42) | - | 28 (18.7) |
| Serum troponin, ug/L | < 0.01 | 55 | - | - | 18 (30.0) |

a: aspartate aminotransferase; b: alanine aminotransferase; c: lactate dehydrogenase; d: α-hydroxybutyric dehydrogenase; e: interleukin-6; f: C-reactive protein; g: erythrocyte sedimentation rate; h: prothrombin time; i: activated partial thromboplastin time; l: interquartile range.

j: White blood cells ($\times 10^9$ cells/L): <28 days old: 10.0–24.0, 29 days–3 years old, 8.0–12.0, >3 years old: 4.0–10.0; Lymphocytes (%): <28 days old: 30–40, 29 days–3 years old: 50–70, >3 years old: 30–40; Hemoglobin ($\times 10^9$/L): <28 days old: 10.0–24.0, 29 days–3 years: 8.0–12.0, >3 years old: 4.0–10.0; LDH: 0–29 days old: 290–2000, 30 days–23 months old: 180–430, >23 months old: 110–290.

k: Creatinine (µmol/L): ≤2 months old: 22–90, 2 months–3 years old: 11–34, 3–15 years old: 21–65, >15 years old:

For peer review only



For peer review only - http://bmjopen.bmj.com/site/about/guidelines.xhtml

male: 64–104, female: 49–90; ESR (mm/h): male: 0–21, female: 0–26.

Figure 1 shows the difference between the results of the first and last laboratory tests for the 233 pediatric COVID-19 cases. The value of liver damage indices (AST [difference: 6.05 IU/L; 95%CI: 1.96–10.14 IU/L; p = 0.004]) and myocardial indices (LDH [difference, 40.33 IU/L; 95%CI, 5.11– 75.55 IU/L; p = 0.026]) were significantly decreased over the disease course. Additionally, the lymphocyte percentage (difference, -2.82%; 95%CI, -4.79% to −0.85%; p < 0.006), platelets count (difference, $-38.74\times10^9$ cells/L; 95%CI, −53.46 to $-24.02\times10^9$ cells/L; p < 0.0001), and prealbumin levels (difference: −25.15 mg/L; 95%CI: −39.41 to –10.89 mg/L; p = 0.001) were significantly increased by the final laboratory test. In order to remove age effects, the study examined the trends of different clinical indicators before and after treatment by age stratification (online supplementary Figure 2).

## Factors impacting the duration to turn negative RT-PCR

Figure 2 shows the distribution in the number of days to turn negative (i.e., the number of days from positive RT-PCR to negative RT-PCR results for SARS-CoV-2) in the 233 pediatric COVID-19 cases based on different classification criteria, including age, sex, and clinical type. This length of duration time from positive RT-PCR to negative for individual patients ranged from 0 to 32 days. The mean lengths of time to turn negative RT-PCR for asymptomatic, mild, moderate, and severe cases were 7.86±4.26, 8.24±4.77, 8.56±3.18, and 15.25±9.57 days, respectively. Regarding patient age, the mean number of days to turn negative was 7.73±7.73 and 8.66±4.76 for the groups aged less than five years old and more than five years old, respectively. Regarding patient sex, the mean length of time to turn negative was 8.43±4.51 and 8.19±4.97 days in male and female patients, respectively.

We constructed Cox proportional hazards regression models to explore the factors that influenced the time to turn negative. The results are shown in Table 3. After controlling for the factors of sex, age, and weight, comparing the



For peer review only - http://bmjopen.bmj.com/site/about/guidelines.xhtml

HRs for the time from positive RT-PCR to negative RT-PCR between patients who had infected family members and those who did not have infected family members yielded an HR and corresponding 95% confidence interval of 0.56 (0.41–0.79). Furthermore, comparisons of patients who had zero infected family members with those who had one, two, or at least three infected family members yielded HRs (95%CI) of 0.47 (0.32–0.69), 0.51 (0.34–0.76), and 0.56 (0.36–0.86), respectively. And a lower HR means a longer time to turn negative. Regarding the clinical disease types, we found the group with severe disease had a significantly longer time to turn negative RT-PCR (HR [95%CI]: 0.14 [0.03–0.59]), after controlling for demographic factors (p = 0.008), while the time from positive RT-PCR to negative of mild and moderate patients was not significantly different from that of asymptomatic patients. The symptom of the occurrence of emesis was significantly associated with the time to turn negative (HR [95%CI]: 0.33 [0.14–0.78]), as was having complications (HR [95%CI]: 0.57 [0.37–0.89]). There was no significant association between use of traditional Chinese medicine (e.g, Forsythia, Honeysuckle, Isatis root) and antiviral drug and time to turn negative (HR [95% CI]: 0.85 [0.64–1.13]).



For peer review only - http://bmjopen.bmj.com/site/about/guidelines.xhtml

**Table 3. Hazard Ratios of Time to Turn Negative RT-PCR from COVID-19 in Cox Proportional Hazards Regression Model.**

| Risk factors | Univariate Cox regression[a] | | Multivariate Cox regression[a, b] | |
|---|---|---|---|---|
| | HR (95% CI) | *p* | HR (95% CI) | *p* |
| **Clinical characteristics** | | | | |
| Sex (versus male) | 1.10 (0.81-1.37) | 0.702 | - | - |
| Age | 0.82 (0.63-1.08) | 0.160 | - | - |
| Contact history | 1.40 (0.96-2.02) | 0.079 | 1.02(0.67-1.57) | 0.918 |
| **Clinical symptoms** | | | | |
| Fever | 0.94 (0.72-1.23) | 0.655 | 0.96(0.72-1.28) | 0.789 |
| Cold | 1.10 (0.27-4.45) | 0.890 | 1.15(0.28-4.67) | 0.844 |
| Diarrhea | 1.70 (0.97-3.13) | 0.063 | 1.56(0.85-2.90) | 0.155 |
| Emesis | 0.51 (0.24-1.11) | 0.089 | 0.33(0.14-0.78) | 0.012 |
| Nausea | 0.85 (0.60-1.20) | 0.348 | 0.76(0.53-1.10) | 0.147 |
| Running nose | 0.98 (0.63-1.54) | 0.934 | 0.94(0.58-1.53) | 0.814 |
| Coughing of phlegm | 0.94 (0.61-1.45) | 0.778 | 0.90(0.57-1.43) | 0.658 |
| Complications | 0.74 (0.50-1.08) | 0.12 | 0.57(0.37-0.89) | 0.013 |
| **Clinical type (versus asymptomatic)** | | | | |
| Mild | 0.91(0.65-1.28) | 0.596 | 0.92(0.65-1.31) | 0.663 |
| Moderate | 0.92(0.56-1.51) | 0.743 | 0.86(0.50-1.48) | 0.591 |
| Severe | 0.29(0.10-0.86) | 0.025 | 0.14(0.03-0.59) | 0.008 |
| **Household contact** | | | | |
| Family member infection | 0.65(0.48-0.89) | 0.006 | 0.56(0.41-0.79) | 0.001 |
| **Number of infectious family member** | | | | |
| 1 | 0.56(0.39-0.80) | 0.002 | 0.47(0.32-0.69) | <0.001 |
| 2 | 0.62(0.43-0.90) | 0.012 | 0.51(0.34-0.76) | 0.001 |
| ≥3 | 0.61(0.41-0.92) | 0.019 | 0.56(0.36-0.86) | 0.008 |
| **Other respiratory pathogen infection** | | | | |
| Combined with virus[c] infection | 0.92 (0.65-1.30) | 0.621 | 0.84(0.59-1.21) | 0.348 |
| Combined with bacterial infection[d] | 1.10 (0.82-1.44) | 0.574 | 1.04(0.78-1.40) | 0.771 |
| **Treatment (versus ATD)** | | | | |
| TCM[e]+ATD[f] | 0.80 (0.61-1.04) | 0.093 | 0.85(0.64-1.13) | 0.259 |

a: Results of Cox proportional hazards regression models are represented as hazard ratios and 95% confidence intervals. Wald tests were performed to calculate the *p*-values for trend.

b: Adjusted for sex, age, and weight.

c: Other detected viruses included adenovirus, influenza virus, parainfluenza virus, and respiratory syncytial virus.

d: Detected bacteria included Legionella pneumophila, Mycoplasma pneumoniae, and Chlamydia pneumoniae.

e: Traditional Chinese Medicine; f: Antiviral Drug

For peer review only



For peer review only - http://bmjopen.bmj.com/site/about/guidelines.xhtml

# Discussion

We found that nearly 70.4% of the pediatric COVID-19 cases were infected through close contact with family members. The pediatric COVID-19 patients in our cohort were diagnosed with mainly mild disease, followed by asymptomatic and moderate disease. The proportion of severe patients was lower compared with that generally observed in adult patients, a pattern which is consistent with that of SARS[6]. The median length of hospital stay in our pediatric COVID-19 patients was 12 days, which is similar to that reported for a pediatric COVID-19 case study in Wuhan[16]. Pediatric COVID-19 patients generally exhibited the clinical symptoms of fever, cough, nausea, and coughing of phlegm, similar to children infected with SARS[6]. It has been reported that adult patients with gastrointestinal symptoms are more likely to develop severe conditions[17] which is consistent with the finding in pediatric patients. Notably, the pediatric COVID-19 cases who exhibited gastrointestinal symptoms (e.g., emesis) took a longer time to be clear from detectable SARS-CoV-2 (HR [95%CI]: 0.33 [0.14–0.78]).

In contrast with a similar study[10], we did find 17 asymptomatic pediatric COVID-19 patients who had abnormal X-ray or CT test results. Although the asymptomatic pediatric COVID-19 patients did not present the typical symptoms of pneumonia, the radiographic examinations were still able to provide some supportive evidence for diagnosis. Since most pediatric COVID-19 patients have mild type disease, routine CT examination is necessary to detect it because a plain X-ray examination may fail to show some lesion details, leading to a misdiagnosis or missed diagnosis[18]. We observed a tendency toward a higher detection of abnormalities in the lungs by X-ray or CT with increasingly more severe disease, evidenced by the detection of abnormalities in radiographic examinations for all the patients with moderate or severe type disease in our study ($p = <0.001$). Additionally, younger pediatric COVID-19 patients were found to be more prone to developing severe conditions ($p < 0.001$). Therefore, if they become infected with SARS-CoV-2, they would be more prone to developing a more severe condition with a more fragile immune system.



For peer review only - http://bmjopen.bmj.com/site/about/guidelines.xhtml

A small proportion of pediatric COVID-19 patients were found to have complications, although the incidence of complications in children with COVID-19 was lower than that observed in adults. Pediatric COVID-19 patients with underlying medical conditions were detected to have some abnormal clinical indices (e.g., elevated AST and procalcitonin levels and longer prothrombin time), and they took longer to turn negative RT-PCR than did those without any complications (HR [95%CI]: 0.57 [0.37–0.89]). We suspected that pediatric COVID-19 patients whose physical functionality may be impaired before infection with SARS-CoV-2, e.g., by an underlying infection or other complications, will need more time to turn negative from COVID-19.

Elevated levels of inflammatory factors such as IL-6 and CRP were observed in the pediatric COVID-19 patients. The reason for this finding could be that cytokine storm plays a role in the immune pathophysiology of COVID-19[18]. Similar immune mechanisms have been reported in previous influenza studies[19]. However, it would need more data to determine if cytokine storms are a major determinant of severity and probably of fatal outcome. Furthermore, some pediatric COVID-19 patients presented with abnormal coagulation function (elevated d-dimer and APTT levels). In contrast with the findings from a study of adult COVID-19 patients[20], the pediatric COVID-19 patients with these abnormalities had mostly mild or asymptomatic type disease, and there was no obvious evidence linking abnormal coagulation function with poor prognosis. Self-controlled studies of pediatric COVID-19 patients found significant increases in lymphocyte levels after treatment, suggesting that lymphocyte numbers are reduced in pediatric COVID-19 cases[10]. It has been suggested that this virus might directly infect lymphocytes, especially T lymphocytes, and initiate or promote lymphocyte cell death, resulting in lymphocyte depletion and a reduced antiviral response[21].

The Cox regression model indicated that the time from positive RT-PCR to negative is strongly influenced by the presence of SARS-CoV-2-infected individuals in their family. This finding suggests that the main transmission



For peer review only - http://bmjopen.bmj.com/site/about/guidelines.xhtml

route for COVID-19 in children is close contact with family members, which is consistent with findings from other research[22]. Besides, the finding proposed a hypothesis that a more intimate transmission scenario is somehow associated with more severe disease which was supported by the conclusion of Bielecki et al that viral inoculum during infection or mode of transmission may be key factors determining the clinical course of COVID-19[23]. We found 59 pediatric COVID-19 cases who was the first SARS-CoV-2-infected individual in their families, which indicated that pediatric COVID-19 patients were at times a primary source of infection, a potential causation pathway often ignored[8]. Because their symptoms are mainly mild and they are sometimes asymptomatic, such cases might be difficult to detect in the early stage of disease. One previous study found that the virus still actively replicates in the gastrointestinal tract in pediatric cases[24], and other evidence also indicates that PCR results for SARS-CoV-2 on feces samples from pediatric COVID-19 patients are positive for longer in children than in adults[22]. Together, these findings suggest that pediatric COVID-19 patients with gastrointestinal symptoms are less likely to turn negative fully from virus infection and have a higher risk of fecal–oral transmission after virus clearance in the respiratory tract.

This study was limited to a small number of pediatric COVID-19 patients, but the prevalence of COVID-19 cases in children is relatively low compared with that in adults, which restricted the number of available subjects. To study as large of a cohort as possible, we enrolled discharged pediatric COVID-19 patients from multiple hospitals. Caution should be exercised in extrapolating these results to other countries, as children in China may have different baseline health conditions and medical care access.

## Conclusion

In conclusion, the pediatric cases with gastrointestinal symptoms, complications or with infected family members will extend the length of duration from positive RT-PCR to negative. We suspected that the infecting viral load



For peer review only - http://bmjopen.bmj.com/site/about/guidelines.xhtml

influences disease severity that emphasized on the importance of social distancing and interventions such as masks. It's difficult to detect asymptomatic pediatric COVID-19 patients; therefore, pose a potential risk to the community.

## Footnotes

**Contributors:** Weibing Wang, Guoying Huang and Zhiping Li conceptualized and designed the study, completed the literature search, critical revised the manuscript for important intellectual content. Hua Xu, Xianfeng Wang, Jinmiao Lu, Yidie Huang, Guangfei Wang, Yanling He, Jie Chen designed the data collection instruments, collected data and are responsible for the integrity of the data, reviewed and revised the manuscript. Shuli Ma, Shangrong Zou, Yuxia Cui, Ruijie Chen, Jin Lu, Xuyuan Li coordinated and supervised data collection, conducted the initial statistical analysis of epidemiology data, reviewed and revised the manuscript. Jianbo Shao, Hong Xu, Zhixi Liu, Xiaohua Ying constructed the Cox proportional hazards regression models and conducted the further statistical analysis of the study, and drafted the manuscript.

All authors approved the final manuscript as submitted to *BMJ Open* with the signature of the corresponding author and agree to be accountable for all aspects of the work.

**Funding:** This study was funded by the Bill & Melinda Gates Foundation [grant number INV-006277]. The funder had no role in the study design, data collection, data analysis, data interpretation, or writing of the report. The corresponding author had full access to all data in the study and had final responsibility for the decision to submit for publication.

**Competing interests:** Jianbo Shao, Hong Xu, Zhixi Liu, Xiaohua Ying, Hua Xu, Xianfeng Wang, Jinmiao Lu, Yidie Huang, Guangfei Wang, Yanling He, Jie Chen, Shuli Ma, Shangrong Zou, Yuxia Cui, Ruijie Chen, Jin Lu, Xuyuan Li, Zhiping Li, Guoying Huang and Weibing Wang declare that they have no conflict of interest.



For peer review only - http://bmjopen.bmj.com/site/about/guidelines.xhtml

**Acknowledgments:** This study was sponsored by the Bill & Melinda Gates Foundation [grant number INV-006277]. We would like to extend my sincere thanks to Professor Stuart MacLeod (University of British Columbia) for his insightful comments. We thank Katie Oakley, PhD, from Liwen Bianji, Edanz Editing China (www.liwenbianji.cn/ac), for editing the English text of a draft of this manuscript.

**Data availability statement:** The data that support the findings of this study are available from the corresponding author, Weibing Wang, upon reasonable request.

For peer review only



For peer review only - http://bmjopen.bmj.com/site/about/guidelines.xhtml

# Reference


1. WHO. Novel Coronavirus (2019-nCoV) situation reports. https://www.who.int/docs/default-source/coronaviruse/situation-reports/20200517-covid-19-sitrep-118.pdf?sfvrsn=21c0dafe_6(accessed May 12, 2020.).

2. WHO. PANDEMIC https://www.who.int/news-room/detail/08-04-2020-who-timeline---covid-19(accessed May 10, 2020).

3. WHO. Fact sheet of Pneumonia. https://www.who.int/en/news-room/fact-sheets/detail/pneumonia(accessed May 10, 2020).

4. NHC P. Report on the development of maternal and child health in China (2019). http://www.nhc.gov.cn/fys/s7901/201905/bbd8e2134a7e47958c5c9ef032e1dfa2.shtml(accessed May 10, 2020; in Chinese).

5. WHO. Consensus document on the epidemiology of severe acute respiratory syndrome (SARS).2003.

6. Hon KLE, Leung CW, Cheng WTF, et al. Clinical presentations and outcome of severe acute respiratory syndrome in children. *The Lancet* 2003;361(9370):1701-03. doi: 10.1016/s0140-6736(03)13364-8

7. Peiris JSM, Lai ST, Poon LLM, et al. Coronavirus as a possible cause of severe acute respiratory syndrome. *The Lancet* 2003;361(9366):1319-25. doi: 10.1016/s0140-6736(03)13077-2

8. Peiris JS, Yuen KY, Osterhaus AD, et al. The severe acute respiratory syndrome. *N Engl J Med* 2003;349(25):2431-41. doi: 10.1056/NEJMra032498

9. Assiri A, Al-Tawfiq JA, Al-Rabeeah AA, et al. Epidemiological, demographic, and clinical characteristics of 47 cases of Middle East respiratory syndrome coronavirus disease from Saudi Arabia: a descriptive study. *Lancet Infect Dis* 2013;13(9):752-61. doi: 10.1016/s1473-3099(13)70204-4

10. Qiu H, Wu J, Hong L, et al. Clinical and epidemiological features of 36 children with coronavirus disease 2019 (COVID-19) in Zhejiang, China: an observational cohort study. *Lancet Infect Dis* 2020 doi: 10.1016/s1473-3099(20)30198-5

11. NHC PRC. Diagnosis and treatment guidelines for 2019 novel coronavirus pneumonia (draft version 5). http://www.nhc.gov.cn/jkj/s3577/202002/a5d6f7b8c48c451c87dba14889b30147/files/3514cb996ae24e2faf65953b4ecd0df4.pdf (access May 20, 2020; in Chinese)

12. WHO. Clinical management of severe acute respiratory infection when novel coronavirus (nCoV) infection is suspected: interim guidance. January 28, 2020.(accessed May 10, 2020)

13. Chen Z, Fu J, Shu Q, et al. Diagnosis and treatment recommendation for pediatric coronavirus disease-19. *Zhejiang Da Xue Xue Bao Yi Xue Ban* 2020;49(1):1-8

14. NHC PRC. National Health and Health Commission of the People's Republic of China. Diagnosis and Treatment Protocol for Novel Coronavirus Pneumonia (Trial Version 7) http://www.nhc.gov.cn/jkj/s3577/202009/318683cbfaee4191aee29cd774b19d8d/files/f9ea38ce2c2d4352bf61ab0feada439f.pdf (access October 10, 2020; in Chinese)

15. Feng F, Dongchi Z, Yu C, et al. Diagnosis and prevention recommendations of Novel Coronavirus 2019 in peidatric patients. *Chinese Journal of Pediatrics* 2020(03):169-74. doi: 10.3760/cma.j.issn.0578-1310.2020.03.001

16. Xia W, Shao J, Guo Y, et al. Clinical and CT features in pediatric patients with COVID-19 infection: Different points from adults. *Pediatr Pulmonol* 2020;55(5):1169-74. doi: 10.1002/ppul.24718

17. Wan Y, Li J, Shen L, et al. Enteric involvement in hospitalised patients with COVID-19 outside Wuhan. *Lancet Gastroenterol Hepatol* 2020 doi: 10.1016/s2468-1253(20)30118-7

18. Li H, Liu L, Zhang D, et al. SARS-CoV-2 and viral sepsis: observations and hypotheses. *The Lancet* 2020 doi:




For peer review only - http://bmjopen.bmj.com/site/about/guidelines.xhtml

10.1016/s0140-6736(20)30920-x

19. Iwasaki A, Pillai PS. Innate immunity to influenza virus infection. *Nat Rev Immunol* 2014;14(5):315-28. doi: 10.1038/nri3665

20. Zhou F, Yu T, Du R, et al. Clinical course and risk factors for mortality of adult inpatients with COVID-19 in Wuhan, China: a retrospective cohort study. *Lancet* 2020;395(10229):1054-62. doi: 10.1016/s0140-6736(20)30566-3

21. Hamming I, Timens W, Bulthuis ML, et al. Tissue distribution of ACE2 protein, the functional receptor for SARS coronavirus. A first step in understanding SARS pathogenesis. *J Pathol* 2004;203(2):631-7. doi: 10.1002/path.1570

22. Su L, Ma X, Yu H, et al. The different clinical characteristics of corona virus disease cases between children and their families in China - the character of children with COVID-19. *Emerg Microbes Infect* 2020;9(1):707-13. doi: 10.1080/22221751.2020.1744483

23. Bielecki M, Zust R, Siegrist D, et al. Social distancing alters the clinical course of COVID-19 in young adults: A comparative cohort study. *Clin Infect Dis* 2020 doi: 10.1093/cid/ciaa889

24. Wu Y, Guo C, Tang L, et al. Prolonged presence of SARS-CoV-2 viral RNA in faecal samples. *The Lancet Gastroenterology & Hepatology* 2020;5(5):434-35. doi: 10.1016/s2468-1253(20)30083-2

For peer review only



For peer review only - http://bmjopen.bmj.com/site/about/guidelines.xhtml

## Figure Legend：

**Figure 1 Comparing the clinical indicators of pediatric COVID-19 patients pre-therapy and post-treatment during hospitalization**

The effect of treatment in 233 pediatric COVID-19 patients. The red and green boxes represent the values of five clinical indicators before and after treatment, respectively. The scattered points on each box represent the values of each clinical indicator for each patient. The middle line of the box represents the medium value of the group. The height of box represents the interquartile range (IQR) of each group. The *p*-value is calculated based on the paired Student's *t*-test.

* represents a *p*-value of less than 0.05; ** represents a *p*-value of less than 0.005; *** represents a *p*-value of less than 0.001.

**Figure 2. Distribution of the time to turn negative of the 233 pediatric COVID-19 patients**

The distribution of the mean time to turn negative RT-PCR for the 233 pediatric COVID-19 patients during the period of admission, split in groups based on different classification criteria. The gradient blue bars represent the mean number of days to turn negative at age ≤5 years and age >5 years. The purple and pink bars represent mean number of days to turn negative for male and female patients, respectively. The four bars on the right represent the mean number of days to turn negative with the four clinical types of COVID-19. The scattered points on each bar represent the number of days to turn negative for each patient in the group. The error lines represent the mean value plus or minus a standard deviation. Turning negative was defined as the time at which a confirmed COVID-19 patient first received negative results for an RT-PCR test for SARS-CoV-2.

For peer review only



For peer review only - http://bmjopen.bmj.com/site/about/guidelines.xhtml

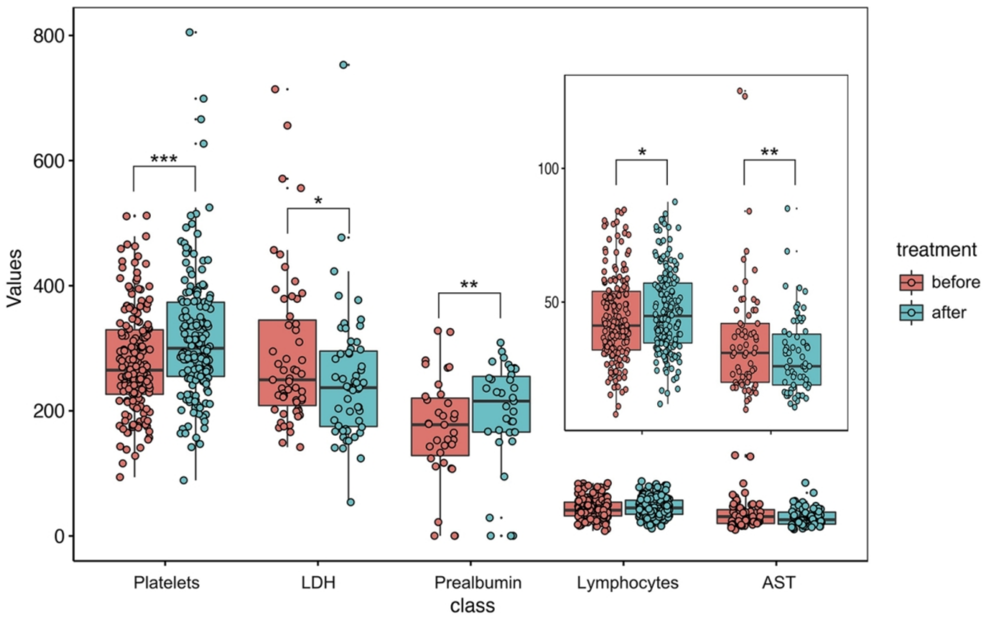


Figure 1 Comparing the clinical indicators of pediatric COVID-19 patients pre-therapy and post-treatment during hospitalization / The effect of treatment in 233 pediatric COVID-19 patients. The red and green boxes represent the values of five clinical indicators before and after treatment, respectively. The scattered points on each box represent the values of each clinical indicator for each patient. The middle line of the box represents the medium value of the group. The height of box represents the interquartile range (IQR) of each group. The p-value is calculated based on the paired Student's t-test.* represents a p-value of less than 0.05; ** represents a p-value of less than 0.005; *** represents a p-value of less than 0.001.

129x81mm (300 x 300 DPI)

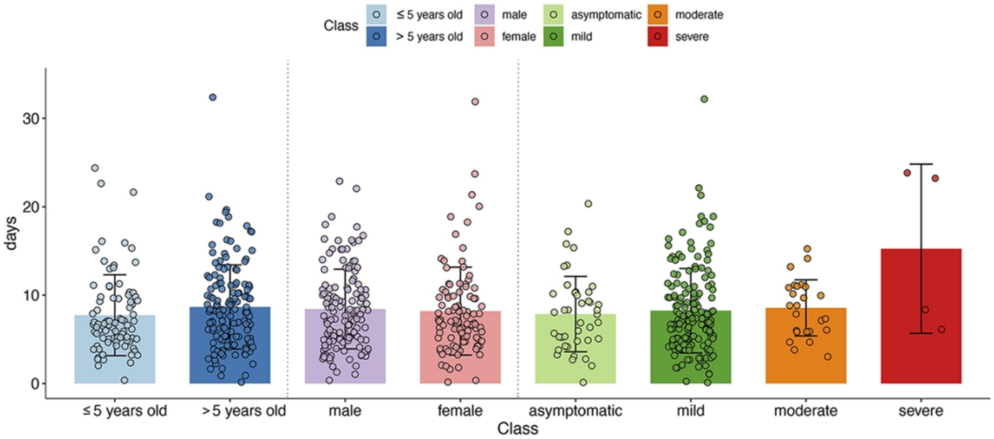


Figure 2. Distribution of the time to turn negative of the 233 pediatric COVID-19 patients / The distribution of the mean time to turn negative RT-PCR for the 233 pediatric COVID-19 patients during the period of admission, split in groups based on different classification criteria. The gradient blue bars represent the mean number of days to turn negative at age ≤5 years and age >5 years. The purple and pink bars represent mean number of days to turn negative for male and female patients, respectively. The four bars on the right represent the mean number of days to turn negative with the four clinical types of COVID-19. The scattered points on each bar represent the number of days to turn negative for each patient in the group. The error lines represent the mean value plus or minus a standard deviation. Turning negative was defined as the time at which a confirmed COVID-19 patient first received negative results for an RT-PCR test for SARS-CoV-2.

109x49mm (300 x 300 DPI)

# Title: Factors associated with the time to turn negative RT-PCR from COVID-19 in pediatric patients

## Authors and Institutions:

Jianbo Shao[a, †], Hong Xu[b, †], Zhixi Liu[c, †], Xiaohua Ying[c, †], Hua Xu[d], Xianfeng Wang[e], Jinmiao Lu[f], Yidie Huang[f], Guangfei Wang[f], Yanling He[g], Jie Chen[h], Shuli Ma[i], Shangrong Zou[j], Yuxia Cui[k], Ruijie Chen[l], Jin Lu[m], Xuyuan Li[n], Zhiping Li[f, *], Guoying Huang [f, *], Weibing Wang [c, *]

[a]Department of Radiological Medicine, Wuhan Children's Hospital (Wuhan Maternal and Child Healthcare Hospital), Tongji Medical College, Huazhong University of Science & Technology, Wuhan 430016, China; and [b]Department of Nephrology, National Children's Medical Center, Children's Hospital of Fudan University, Shanghai 201102, China; and [c]School of Public Health, Fudan University, Shanghai 200032, China; and [d]Department of Pharmacy, Wuhan Children's Hospital (Wuhan Maternal and Child Healthcare Hospital), Tongji Medical College, Huazhong University of Science & Technology, Wuhan 430016, China; and [e]Department of Pediatrics, The Third People's Hospital of Shenzhen, Shenzhen 518020, China; and [f]Department of Clinical Pharmacy, National Children's Medical Center, Children's Hospital of Fudan University, Shanghai 201102, China; and [g]Department of Pharmacy, Guangzhou Women and Children's Medical Center, Guangzhou 510623, China; and [h]Department of Pharmacy, The Third People's Hospital of Kunming, Kunming 650000, China; and [i]Department of Pharmacy, Children's Hospital Affiliated to Zhengzhou University, Zhengzhou 450000, China; and [j]Department of Pharmacy, Guangzhou Eighth People's Hospital, Guangzhou 510060, China; and [k]Department of Pediatrics, Guizhou Provincial People's Hospital, Guiyang 550002, China; and [l]Wenzhou Municipal Key Laboratory of Paediatric Pharmacy,

For peer review only

Department of Pharmacy, The Second Affiliated Hospital and Yuying Children's Hospital of Wenzhou Medical University, Wenzhou 325027, China; and [m]Department of Pharmacy, The First Affiliated Hospital of USTC, Division of Life Sciences and Medicine, University of Science and Technology of China, Anhui 230001, China; and [n]Department of Biology, University of California, Santa Barbara 93101, California, the United States

[†] Jianbo Shao, Hong Xu, Zhixi Liu, Xiaohua Ying contributed equally to this manuscript

[*] Zhiping Li, Guoying Huang, Weibing Wang contributed equally to corresponding work of this manuscript

## Corresponding author:

Dr. Zhiping Li, PhD, Department of Clinical Pharmacy, National Children's Medical Center, Children's Hospital of Fudan University, Shanghai 201102, China

Email: zpli@fudan.edu.cn

Dr. Guoying Huang*, MD, PhD, Centre of Cardiovascular, National Children's Medical Center, Children's Hospital of Fudan University, Shanghai 201102, China

Email: gyhuang@shmu.edu.cn

Dr. Weibing Wang, PhD, Department of Epidemiology, School of Public Health, Fudan University, 138 Yi Xue Yuan Road, Shanghai 200032, China, 86-21-54237811

Email: wwb@fudan.edu.cn

## Contents:

Supplementary Table 1. Definitions of clinical types for COVID-19 pediatric patients
Supplementary Figure 1. The spatial distribution of the 12 COVID-19 tertiary hospitals
Supplementary Figure 2. Comparison of clinical indicators stratified by age pre-therapy and post-treatment

For peer review only

**Supplementary Table 1. Definitions of clinical types for COVID-19 pediatric patients**

| Classification of clinical types | Based on the clinical characteristics of confirmed COVID-19 pediatric cases |
|---|---|
| Asymptomatic disease | • No clinical symptoms or signs<br>• Normal chest imaging examination<br>• Positive RT-PCR test for SARS-CoV-2 or positive result for retrospectively diagnosed sero-specific antibody |
| Mild disease | • Acute upper respiratory tract infection (*e.g.*, fever, fatigue, myalgia, cough, sore throat, runny nose and sneezing, *etc.*)<br>• Pharyngeal congestion<br>• No positive signs of lungs<br>• Some may have no fever, or only other gastrointestinal symptoms (*e.g.*, nausea, vomiting, abdominal pain and diarrhea) |
| Moderate disease | • Pneumonia<br>• Clinical symptoms such as fever, cough<br>• Wheezing, dry and (or) moist rales were heard in the lungs<br>• Sub-clinical: some without clinical symptoms or signs but with abnormal pulmonary lesions |
| Severe disease | • Early symptoms of respiratory tract (*e.g.*, fever and cough, *etc*); may be accompanied by digestive tract symptoms (e.g., diarrhea, *etc*)<br>• Progression of disease within a week or so, with dyspnea, central cyanosis, hypoxia, pulse oximetry <0.92 |
| Critical disease | • ARDS<br>• Respiratory failure<br>• Multiple organ dysfunction (*e.g.*, shock, encephalopathy, myocardial injury or failure, coagulation dysfunction and acute kidney injury) |

For peer review only

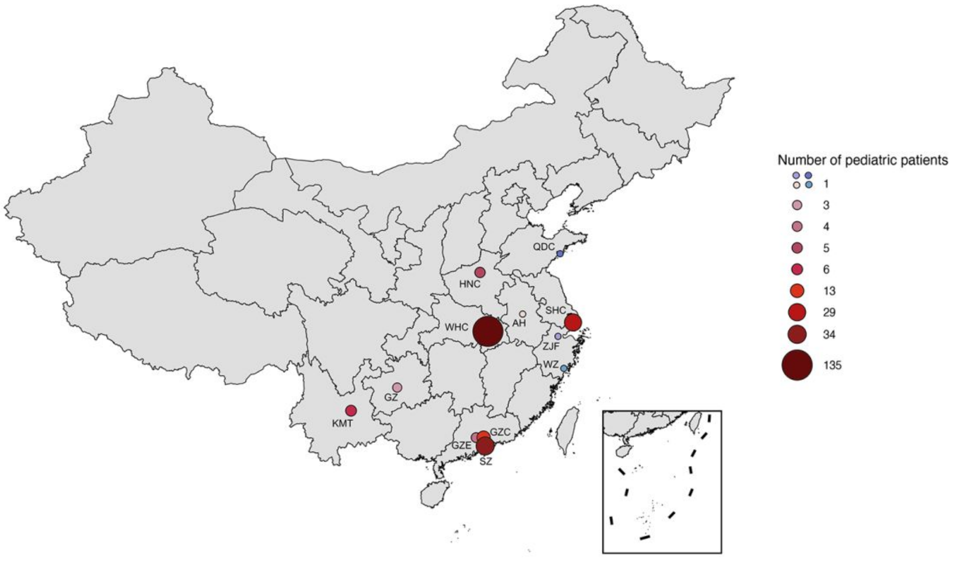


**Supplementary Figure 1. The spatial distribution of the 12 COVID-19 tertiary hospitals**

The number of patients enrolled in the study is varied by the size of the points and the hospitals varied by the color of the points. Abbreviations: WHC: Wuhan Children's Hospital (Wuhan Maternal and Child Healthcare Hospital), Tongji Medical College, Huazhong University of Science & Technology; SZ: The Third People's Hospital of Shenzhen; SHC: National Children's Medical Center, Children's Hospital of Fudan University; GZC: Guangzhou Women and Children's Medical Center; KMT: The Third People's Hospital of Kunming; HNC: Children's Hospital Affiliated to Zhengzhou University; GZE: Guangzhou Eighth People's Hospital; GZ: Guizhou Provincial People's Hospital; QDC: Qingdao Children's Hospital; AH: The First Affiliated Hospital of USTC, Division of Life Sciences and Medicine, University of Science and Technology of China; ZJF: The First Hospital of Zhejiang University and WZ: The Second Affiliated Hospital and Yuying Children's Hospital of Wenzhou Medical University.

For peer review only

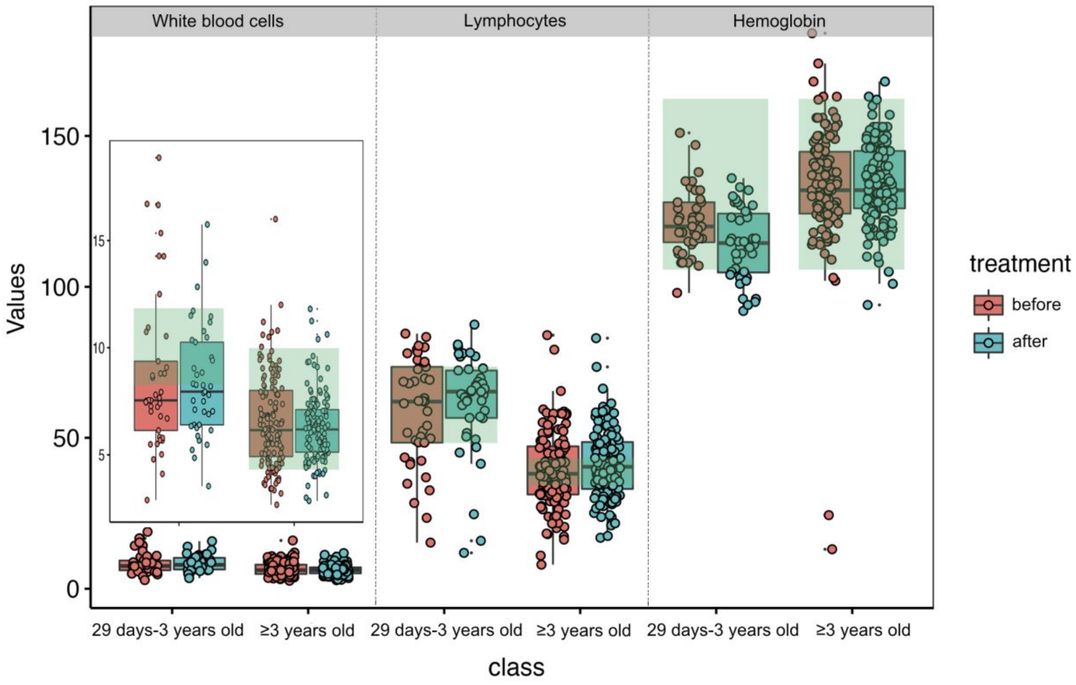


**Supplementary Figure 2. Comparison of clinical indicators stratified by age pre-therapy and post-treatment**

The red and green boxes represent the clinical indicator values before and after treatment, respectively. The scattered points on each box represent the values of each clinical indicator for each patient. The shaded green rectangle represents the normal reference range for an indicator for that age group. The middle line of the box represents the medium value of the group. The height of the box represents the interquartile range (IQR) of each group.

**STROBE 2007 (v4) Statement—Checklist of items that should be included in reports of *cohort studies***

| Section/Topic | Item # | Recommendation | Reported on page # |
|---|---|---|---|
| **Title and abstract** | 1 | (*a*) Indicate the study's design with a commonly used term in the title or the abstract | 3 |
| | | (*b*) Provide in the abstract an informative and balanced summary of what was done and what was found | 3 |
| **Introduction** | | | |
| Background/rationale | 2 | Explain the scientific background and rationale for the investigation being reported | 5 |
| Objectives | 3 | State specific objectives, including any prespecified hypotheses | 5 |
| **Methods** | | | |
| Study design | 4 | Present key elements of study design early in the paper | 5-7 |
| Setting | 5 | Describe the setting, locations, and relevant dates, including periods of recruitment, exposure, follow-up, and data collection | 5-7 |
| Participants | 6 | (*a*) Give the eligibility criteria, and the sources and methods of selection of participants. Describe methods of follow-up | 5-7 |
| | | (*b*) For matched studies, give matching criteria and number of exposed and unexposed | |
| Variables | 7 | Clearly define all outcomes, exposures, predictors, potential confounders, and effect modifiers. Give diagnostic criteria, if applicable | 5-7 |
| Data sources/ measurement | 8* | For each variable of interest, give sources of data and details of methods of assessment (measurement). Describe comparability of assessment methods if there is more than one group | 5-8 |
| Bias | 9 | Describe any efforts to address potential sources of bias | |
| Study size | 10 | Explain how the study size was arrived at | 5 |
| Quantitative variables | 11 | Explain how quantitative variables were handled in the analyses. If applicable, describe which groupings were chosen and why | 7-8 |
| Statistical methods | 12 | (*a*) Describe all statistical methods, including those used to control for confounding | 7-8 |
| | | (*b*) Describe any methods used to examine subgroups and interactions | 7-8 |
| | | (*c*) Explain how missing data were addressed | 7-8 |
| | | (*d*) If applicable, explain how loss to follow-up was addressed | 7-8 |
| | | (*e*) Describe any sensitivity analyses | |
| **Results** | | | |

For peer review only

| | | | |
|---|---|---|---|
| Participants | 13* | (a) Report numbers of individuals at each stage of study—eg numbers potentially eligible, examined for eligibility, confirmed eligible, included in the study, completing follow-up, and analysed | 8-10 |
| | | (b) Give reasons for non-participation at each stage | |
| | | (c) Consider use of a flow diagram | |
| Descriptive data | 14* | (a) Give characteristics of study participants (eg demographic, clinical, social) and information on exposures and potential confounders | 8-9 |
| | | (b) Indicate number of participants with missing data for each variable of interest | |
| | | (c) Summarise follow-up time (eg, average and total amount) | |
| Outcome data | 15* | Report numbers of outcome events or summary measures over time | 9-13 |
| Main results | 16 | (*a*) Give unadjusted estimates and, if applicable, confounder-adjusted estimates and their precision (eg, 95% confidence interval). Make clear which confounders were adjusted for and why they were included | 13-15 |
| | | (*b*) Report category boundaries when continuous variables were categorized | |
| | | (*c*) If relevant, consider translating estimates of relative risk into absolute risk for a meaningful time period | |
| Other analyses | 17 | Report other analyses done—eg analyses of subgroups and interactions, and sensitivity analyses | |
| **Discussion** | | | |
| Key results | 18 | Summarise key results with reference to study objectives | 16-18 |
| **Limitations** | | | |
| Interpretation | 20 | Give a cautious overall interpretation of results considering objectives, limitations, multiplicity of analyses, results from similar studies, and other relevant evidence | 18-19 |
| Generalisability | 21 | Discuss the generalisability (external validity) of the study results | 18-19 |
| **Other information** | | | |
| Funding | 22 | Give the source of funding and the role of the funders for the present study and, if applicable, for the original study on which the present article is based | 19-20 |

*Give information separately for cases and controls in case-control studies and, if applicable, for exposed and unexposed groups in cohort and cross-sectional studies.

**Note:** An Explanation and Elaboration article discusses each checklist item and gives methodological background and published examples of transparent reporting. The STROBE checklist is best used in conjunction with this article (freely available on the Web sites of PLoS Medicine at http://www.plosmedicine.org/, Annals of Internal Medicine at http://www.annals.org/, and Epidemiology at http://www.epidem.com/). Information on the STROBE Initiative is available at www.strobe-statement.org.

For peer review only

BMJ Open

# Factors associated with the time to return negative RT-PCR from COVID-19 in pediatric patients: a retrospective cohort study

| | |
|---|---|
| Journal: | *BMJ Open* |
| Manuscript ID | bmjopen-2021-052609.R1 |
| Article Type: | Original research |
| Date Submitted by the Author: | 14-Aug-2021 |
| Complete List of Authors: | Shao, Jianbo; Huazhong University of Science and Technology Tongji Medical College Affiliated Wuhan Children's Hospital<br>Xu, Hong; Children's Hospital of Fudan University, Pediatric Nephrology<br>Liu, Zhixi; Fudan University School of Public Health<br>YING, Xiaohua; Fudan University School of Public Health<br>Xu, Hua; Huazhong University of Science and Technology Tongji Medical College Affiliated Wuhan Children's Hospital, Department of Pharmacy<br>Wang, Xianfeng; The Third People's Hospital of Shenzhen<br>Lu, Jinmiao; Children's Hospital of Fudan University, Department of Clinical Pharmacy<br>Huang, Yidie; Children's Hospital of Fudan University, Department of Clinical Pharmacy<br>Wang, Guangfei; Children's Hospital of Fudan University, Department of Clinical Pharmacy<br>He, Yanling; Guangzhou Women and Children's Medical Center, Department of Pharmacy<br>Chen, Jie; Dali University, Affiliated Sixth People's Hospital<br>Ma, Shuli; Zhengzhou University, Affiliated Children’s Hospital<br>Zou, Shangrong; Guangzhou Eighth People's Hospital<br>Cui, Yuxia; Guizhou Provincial People's Hospital<br>Chen, Ruijie; Yuying Children's Hospital of Wenzhou Medical College<br>Lu, Jin; University of Science and Technology of China<br>Li, Xuyuan; University of California Santa Barbara<br>Li, Zhiping; Children's Hospital of Fudan University, Department of Clinical Pharmacy<br>Huang, Guoying; Children's Hospital of Fudan University, Department of Clinical Pharmacy<br>Wang, Weibing; Fudan University School of Public Health, Department of Pediatrics |
| <b>Primary Subject Heading</b>: | Paediatrics |
| Secondary Subject Heading: | Epidemiology, Infectious diseases |
| Keywords: | COVID-19, Epidemiology < INFECTIOUS DISEASES, PAEDIATRICS |
| | |

SCHOLARONE™
Manuscripts

*I, the Submitting Author has the right to grant and does grant on behalf of all authors of the Work (as defined in the below author licence), an exclusive licence and/or a non-exclusive licence for contributions from authors who are: i) UK Crown employees; ii) where BMJ has agreed a CC-BY licence shall apply, and/or iii) in accordance with the terms applicable for US Federal Government officers or employees acting as part of their official duties; on a worldwide, perpetual, irrevocable, royalty-free basis to BMJ Publishing Group Ltd ("BMJ") its licensees and where the relevant Journal is co-owned by BMJ to the co-owners of the Journal, to publish the Work in this journal and any other BMJ products and to exploit all rights, as set out in our licence.*

*The Submitting Author accepts and understands that any supply made under these terms is made by BMJ to the Submitting Author unless you are acting as an employee on behalf of your employer or a postgraduate student of an affiliated institution which is paying any applicable article publishing charge ("APC") for Open Access articles. Where the Submitting Author wishes to make the Work available on an Open Access basis (and intends to pay the relevant APC), the terms of reuse of such Open Access shall be governed by a Creative Commons licence – details of these licences and which Creative Commons licence will apply to this Work are set out in our licence referred to above.*

*Other than as permitted in any relevant BMJ Author's Self Archiving Policies, I confirm this Work has not been accepted for publication elsewhere, is not being considered for publication elsewhere and does not duplicate material already published. I confirm all authors consent to publication of this Work and authorise the granting of this licence.*

For peer review only

# Title: Factors associated with the time to return negative RT-PCR from COVID-19 in pediatric patients: a retrospective cohort study

## Authors and Institutions:

Jianbo Shao[a, †], Hong Xu[b, †], Zhixi Liu[c, †], Xiaohua Ying[c, †], Hua Xu[d], Xianfeng Wang[e], Jinmiao Lu[f], Yidie Huang[f], Guangfei Wang[f], Yanling He[g], Jie Chen[h], Shuli Ma[i], Shangrong Zou[j], Yuxia Cui[k], Ruijie Chen[l], Jin Lu[m], Xuyuan Li[n], Zhiping Li[f, *], Guoying Huang [f, *], Weibing Wang [c, *]

[a]Department of Radiological Medicine, Wuhan Children's Hospital (Wuhan Maternal and Child Healthcare Hospital), Tongji Medical College, Huazhong University of Science & Technology, Wuhan 430016, China; and [b]Department of Nephrology, National Children's Medical Center, Children's Hospital of Fudan University, Shanghai 201102, China; and [c]School of Public Health, Fudan University, Shanghai 200032, China; and [d]Department of Pharmacy, Wuhan Children's Hospital (Wuhan Maternal and Child Healthcare Hospital), Tongji Medical College, Huazhong University of Science & Technology, Wuhan 430016, China; and [e]Department of Pediatrics, The Third People's Hospital of Shenzhen, Shenzhen 518020, China; and [f]Department of Clinical Pharmacy, National Children's Medical Center, Children's Hospital of Fudan University, Shanghai 201102, China; and [g]Department of Pharmacy, Guangzhou Women and Children's Medical Center, Guangzhou 510623, China; and [h]Department of Pharmacy, The Third People's Hospital of Kunming, Kunming 650000, China; and [i]Department of Pharmacy, Children's Hospital Affiliated to Zhengzhou University, Zhengzhou 450000, China; and [j]Department of Pharmacy, Guangzhou Eighth People's Hospital, Guangzhou 510060, China; and [k]Department of Pediatrics, Guizhou Provincial People's Hospital, Guiyang 550002, China; and [l]Wenzhou Municipal Key Laboratory of Paediatric Pharmacy, Department of Pharmacy, The Second Affiliated Hospital and Yuying Children's Hospital of Wenzhou Medical University, Wenzhou 325027, China; and [m]Department of Pharmacy,

For peer review only



For peer review only - http://bmjopen.bmj.com/site/about/guidelines.xhtml

The First Affiliated Hospital of USTC, Division of Life Sciences and Medicine, University of Science and Technology of China, Anhui 230001, China; and [n]Department of Biology, University of California, Santa Barbara 93101, California, the United States

† Jianbo Shao, Hong Xu, Zhixi Liu, Xiaohua Ying contributed equally to this manuscript

* Zhiping Li, Guoying Huang, Weibing Wang contributed equally to corresponding work of this manuscript

## Corresponding author:

Dr. Zhiping Li, PhD, Department of Clinical Pharmacy, National Children's Medical Center, Children's Hospital of Fudan University, Shanghai 201102, China

Email: zpli@fudan.edu.cn

Dr. Guoying Huang*, MD, PhD, Centre of Cardiovascular, National Children's Medical Center, Children's Hospital of Fudan University, Shanghai 201102, China

Email: gyhuang@shmu.edu.cn

Dr. Weibing Wang, PhD, Department of Epidemiology, School of Public Health, Fudan University, 138 Yi Xue Yuan Road, Shanghai 200032, China, 86-21-54237811

Email: wwb@fudan.edu.cn

Word count: 3229



For peer review only - http://bmjopen.bmj.com/site/about/guidelines.xhtml

# Abstract

**Objective:** This study aimed to describe the epidemiological and clinical features and potential factors related to the time to return negative RT-PCR in discharged pediatric patients with COVID-19.

**Design:** Retrospective cohort study

**Setting:** Unscheduled admissions to 12 tertiary hospitals in China.

**Participants:** Two hundred and thirty-three clinical charts of pediatric patients with confirmed diagnosis of COVID 19 admitted from January 1, 2020 to April 17, 2020.

**Primary and secondary outcome measures:** Primary outcome measures: factors associated with the time to return negative RT-PCR from COVID-19 in pediatric patients. Secondary outcome measures: epidemiological and clinical features and laboratory results in pediatric patients.

**Results** The median age of patients in our cohort was 7·50 (IQR: 2·92,12·17) years and 133 (57·1%) patients were male. 42 (18.0%) patients were evaluated as asymptomatic, while 162 (69.5%) and 25 (10.7%) patients were classified as mild or moderate, respectively. In Cox regression analysis, In Cox regression analysis, longer time to negative RT-PCR was associated with the presence of confirmed infection in family members (Hazard Ratio (HR) [95%CI]: 0.56 [0.41–0.79]). Pediatric patients with emesis symptom had a longer time to return negative (HR [95%CI]: 0.33 [0.14–0.78]). During hospitalization, the use of traditional Chinese medicine (TCM) and antiviral drugs at the same time is not more conducive to return negative than antiviral drugs alone (HR [95%CI]: 0.85[0.64-1.13]).

**Conclusions** The mode of transmission might be a critical factor determining the disease severity of COVID-19. Patients with emesis symptom, complications or confirmed infection in family members may have longer healing times than others. However, no significant favorable effects from TCM when the patients have received antiviral treatment.




For peer review only - http://bmjopen.bmj.com/site/about/guidelines.xhtml

**Keywords** SARS-CoV-2; COVID-19; infectious disease; pediatrics

For peer review only



For peer review only - http://bmjopen.bmj.com/site/about/guidelines.xhtml

## Strengths and limitations of this study

- This study enrolled all pediatric COVID-19 patients (n = 233) discharged from 12 tertiary hospitals across 11 cities and found pediatric patients with emesis symptom had a longer time to return negative.
- We determined the in-family transmission is the main infection route for pediatric COVID-19 cases.
- Transmission mode may be key factors determining the clinical course of COVID-19.

For peer review only



For peer review only - http://bmjopen.bmj.com/site/about/guidelines.xhtml

# Introduction

Coronavirus disease 2019 (COVID-19) is an emerging infectious disease with high transmissibility. As of 11 August 2021, the cumulative number of cases worldwide has reached 203,944,144 with 121,960 of those cases in China[1]. On March 11, 2021, the World Health Organization (WHO) further announced the epidemic of COVID-19 would be classified as a global pandemic[2]. According to the data released by the WHO[3], pneumonia is the leading cause of death among children worldwide and the second among children under five in China[4]. Pediatric cases were reported during the epidemics of SARS-CoV (severe acute respiratory syndrome coronavirus)[5 6]. In pediatric SARS patients, the clinical symptoms of teenagers were similar to those of adults[7]. Furthermore, they maintained stable conditions throughout the disease course and had good prognoses[8]. Another study demonstrated that pediatric cases accounted for only 2% of all Middle East Respiratory Syndrome cases[9]. One previous study that enrolled 36 COVID-19 pediatric cases concluded that the close contacts with family members might be difficult to avoid in daily life[10].

The low proportion of pediatric patients among COVID-19 cases has perplexed clinicians, epidemiologists, and scientists. Current understanding regarding the duration of positive RT-PCR to negative for COVID-19 is limited, and this knowledge gap is particularly notable among children. Therefore, we aimed to explore and analyze the clinical and epidemiological characteristics of 233 discharged pediatric patients to reveal corresponding information for the prevention and treatment of pediatric COVID-19 patients.

# Materials and Methods

## Study population

This multi-center retrospective study was conducted in 12 tertiary hospitals in 11 Chinese cities. We collected information about the discharged pediatric COVID-19 patients treated in these hospitals from January 1, 2020 to



For peer review only - http://bmjopen.bmj.com/site/about/guidelines.xhtml

April 17, 2020. The pediatric patients with confirmed COVID-19 were diagnosed based on guidelines issued by the National Health Commission of the People's Republic of China[11].

## Procedure

Relevant medical information was collected from electronic medical records and reviewed by trained doctors to ensure the quality of the medical data. Retrieved information included the demographic information, COVID-19 contact history (e.g., history of travel to or residence in the COVID-19 epidemic areas or history of contact with SARS-CoV-2-infected individuals), complications (*e.g.*, myocardial injury[12]: the elevation of cardiac troponin, with at least one value above the 99th percentile upper reference limit; liver insufficiency[13]: the inability of the liver to perform its normal synthetic and metabolic functions, *etc.*), laboratory tests results, chest computed tomography and B-scan ultrasonography results. Laboratory tests included blood routine examination and examinations for lung, liver, myocardial, and kidney function, such as measurements of the aspartate amino-transferase (AST), alanine amino-transferase (ALT), lactate dehydrogenase (LDH), α-hydroxybutyrate dehydrogenase (α-HBDH) and the erythrocyte sedimentation rate (ESR).

The preliminary diagnosis of suspected COVID-19 was based on clinical symptoms and potential exposure via travel to COVID-19 epidemic areas or close contact with a confirmed case. The diagnosis was subsequently confirmed based on results from real-time reverse transcriptase-polymerase chain reaction (RT-PCR) tests for SARS-CoV-2 (severe acute respiratory syndrome coronavirus 2) on nasal or pharyngeal swab specimens from all suspected patients diagnosed by the previous step. RT-PCR is recommended by the Center for Disease Control and Prevention (CDC)[11] and WHO interim guidance as a solid method for confirming COVID-19 diagnosis[14]. RT-PCR testing was performed to detect infection with other viruses (i.e., adenovirus, influenza virus, parainfluenza virus, and respiratory syncytial virus) and performed sputum culture to test for potential infections with bacteria,



For peer review only - http://bmjopen.bmj.com/site/about/guidelines.xhtml

including *Legionella pneumophila*, *Mycoplasma pneumoniae*, and *Chlamydia pneumoniae*. We evaluated the clinical type of each admitted pediatric COVID-19 patient according to the recommendations issued by the pediatrics branch of the Chinese Medical Association[15]. Patients were described as having one of four clinical types of COVID-19, i.e., asymptomatic, mild, moderate and severe disease, based on their RT-PCR results, clinical symptoms and radiological results (online supplementary Table 1)[16 17]. Once admitted, these pediatric patients were given available antiviral treatments (e.g., Ribavirin aerosol, Oseltamivir granule, etc.) and oxygen if necessary (blood oxygen saturation $<0.92$). The discharge criteria were that the patient must have obtained two consecutive negative RT-PCR test results.

## Statistical analysis

Categorical variables are presented as number and frequency rate using the Fisher exact test or Chi-square test. Continuous variables with non-normal distribution are presented as the median and interquartile range (IQR) and were compared using the Kruskal-Wallis Rank Sum Test. In addition, we analyzed the first and last laboratory test results to examine the effect of treatment during hospitalization. These two test results were compared by a paired Student's t-test or Wilcoxon's signed rank test.

Cox proportional hazards regression models were used to estimate hazard ratios (HR) and corresponding 95% confidence intervals (CI) to explore associations between risk factors and the number of days to return negative RT-PCR for the 233 pediatric COVID-19 cases. In Cox regression models, the interpretation of results is that the endpoint event we observed was negative RT-PCR (benign event); if the HR value of a variable is less than 1, the variable is not conducive to the occurrence of the outcome of negative RT-PCR, and there will be a longer day to return negative, and vice versa. Time to return negative was defined as the time from positive RT-PCR results to negative RT-PCR results for SARS-CoV-2 during the hospitalization. During the analysis, sample size varied



For peer review only - http://bmjopen.bmj.com/site/about/guidelines.xhtml

because of missing data; the sample sizes for each analysis are listed in Table 1. The variables assessed in the statistical analysis were contact history, clinical symptoms, and clinical indicators. Univariate and multivariate analyses adjusted for sex, age, and weight were carried out by Cox proportional hazard ratio models to examine significant associations. These analyses conducted by R-packages 'dplyr', a data manipulation package, will provide any information about factors that contribute to a longer duration of positive RT-PCR to negative; 'survival', 'survminer' helped to determine the risk factors that contributed to a longer time of positive RT-PCR.

All analyses were conducted on the subjects who lacked missing data, and only differences with two-sided α and a p-value less than 0.05 was considered statistically significant. All analyses were conducted with the use of R software version 3.6.2.

### Patient and Public Involvement

Patients and/or the public were not involved in the design, or conduct, or reporting or dissemination plans of this research.

## Results

### Demographic and clinical characteristics

From January 1, 2020 and April 17, 2020, 233 pediatric COVID-19 cases were discharged from the 12 study hospitals (online supplementary Figure 1). These cases with a median age of 7.50 (IQR: 2.92,12.17) years ranged from 0.05 to 18.75 years old of which ~~and~~ 133 (57.1%) cases were male patients. Most patients (n = 199 [85.4%]) had a COVID-19-contact history, including contact with people from Wuhan/Hubei or travel to Wuhan/Hubei. 59 (26.5%) of the pediatric patients were the first SARS-CoV-2-infected individual of their family. Additionally, 40 (17.2%) patients had a simultaneous infection with an additional virus, and 79 (33.9%) patients had a simultaneous bacterial infection. None of the pediatric patients in our study were found to be at risk of death due to COVID-19



For peer review only - http://bmjopen.bmj.com/site/about/guidelines.xhtml

during hospitalization (Table 1).

**Table 1. Clinical Features of 233 Pediatric Patients Infected with SARS-CoV-2**

| Clinical factors | | All patients N (%) 233(100.0) | Asymptomatic N (%) 42(18.0) | Mild[c] N (%) 162(69.5) | Moderate Severe N (%) 29(12.4) | p[e] |
|---|---|---|---|---|---|---|
| Sex | Male | 133(57.1) | 29(69.0) | 88(54.3) | 16(55.2) | 0.223 |
| | Female | 100(42.9) | 13(31.0) | 74(45.7) | 13(44.8) | |
| Age, y | ≤ 5 | 84(36.1) | 6(14.3) | 61(37.7) | 17(58.6) | <0.001 |
| | > 5 | 149(63.9) | 36(85.7) | 101(62.3) | 12(41.4) | |
| Age, median (IQR), y | | 7.50(2.92,12.17) | 9.37(7.00,12.15) | 7.92(2.85,12.29) | 4.00(0.85,8.92) | 0.010[f] |
| Contact history of epidemic area | Yes | 199(85.4) | 41(97.6) | 132(81.5) | 26(89.7) | 0.015[g] |
| Number of infected family member | 0 | 59(26.5) | 4(9.8) | 46(29.5) | 9(34.6) | 0.185 |
| | 1 | 67(30.0) | 16(39.0) | 43(27.6) | 8(30.8) | |
| | 2 | 54(24.2) | 10(24.4) | 39(25.0) | 5(19.2) | |
| | ≥ 3 | 43(19.3) | 11(26.8) | 28(17.9) | 4(15.4) | |
| Combined with virus infection[a] | Yes | 40(17.2) | 2(4.8) | 31(19.1) | 7(24.1) | 0.039 |
| Combined with bacterial infection[b] | Yes | 79(33.9) | 13(31.0) | 60(37.0) | 6(20.7) | 0.142 |
| Fever | Yes | 86(36.9) | 0(0.0) | 69(42.6) | 17(58.6) | <0.001 |
| Cough | Yes | 83(35.6) | 0(0.0) | 68(42.0) | 15(51.7) | <0.001 |
| Running nose | Yes | 21(9.0) | 0(0.0) | 17(10.5) | 4(13.8) | 0.027[g] |
| Diarrhea | Yes | 12(5.2) | 0(0.0) | 9(5.6) | 3(10.3) | 0.127[g] |
| Nausea | Yes | 38(16.3) | 0(0.0) | 32(19.8) | 6(20.7) | 0.001[g] |
| Coughing of phlegm | Yes | 23(9.9) | 0(0.0) | 16(9.9) | 7(24.1) | 0.002[g] |
| Pharyngitis | Yes | 13(5.6) | 0(0.0) | 10(6.2) | 3(10.3) | 0.111[g] |
| X-ray or CT test findings | Abnormal | 139(59.7) | 17(40.5) | 94(58.0) | 28(96.6) | <0.001 |
| Complications | Yes | 31(13.3) | 3(7.1) | 22(13.6) | 6(20.7) | 0.268 |
| Days in hospital, median (IQR), d | | 12.00(9.00,17.00) | 10.50(8.25,14.00) | 12.00(9.00,17.00) | 13.00(9.75,17.00) | 0.416[f] |

a: Adenovirus, influenza virus, parainfluenza virus, respiratory syncytial virus, *etc*.

b: Legionella pneumophila, Mycoplasma pneumoniae, Chlamydia pneumoniae, *etc*.

c: Mild disease: upper respiratory symptoms for a short duration or asymptomatic infection; positive RT-PCR test for SARS-CoV-2; no abnormal radiographic and septic presentation.

d: Moderate disease: mild pneumonia; symptoms such as fever, cough, fatigue, headache, and myalgia; no complications and manifestations related to severe conditions.

e: p-values indicate the difference among four different disease types in pediatric COVID-19 patients with pneumonia.

f: p-value was obtained from a Kruskal-Wallis test; [g]: p-values were obtained from Fisher exact tests.

The most common clinical symptoms of the 233 pediatric COVID-19 patients were fever (40.8%), cough (35.6%)



For peer review only

For peer review only - http://bmjopen.bmj.com/site/about/guidelines.xhtml

and nausea (16.3%). The symptoms of running nose, diarrhea, and pharyngitis were also reported. All patients underwent a chest X-ray or CT test, and the results suggest that 139 (59.7%) patients had lung abnormalities, which include 17(40.5%) of asymptomatic patients, 94 (58.0%) of mild patients, and 28 (96.6%) of moderate or severe patients. The lung abnormalities included ground-glass opacities and patchy shadows or multiple plaques in some of the lung lobes on a CT scan. The median length of the patient hospital stay was 12 days (IQR: 9–17 days). An examination of patients' complications revealed that 31 of the 233 pediatric COVID-19 patients had complications (e.g., myocardial injury and liver insufficiency, etc.), including 3 (7.1%) asymptomatic patients, 22 (13.6%) mild patients, and 6 (20.7%) moderate or severe patients.

The clinical disease was classified as asymptomatic in 42 (18.0%) patients, mild in 162 (69.5%) patients, moderate in 25 (10.7%) patients and severe in 4 (1.7%) patients. As the severity of disease increased, the median age (p = 0.010) decreased and the proportion of children under 5 years old (p < 0.001) increased. Notably, there was no significant sex difference in severity of disease (p = 0.223). When comparing between children with or without infected family members, the severity of illness exhibited no significant correlation. Among the patients with moderate or severe disease, 17 (58.6%) had fever, 15 (51.7%) had cough and 7 (24.1%) had coughing of phlegm. Only 13.8% and 10.3% had the symptoms of running nose and diarrhea, respectively. Of the mild cases, 69 (42.6%), 68 (42.0%), and 32 (19.8%) patients had fever, cough, and nausea, respectively. In contrast, none of the asymptomatic patients had any of the clinical symptoms that were common in the patients with mild or moderate disease.

## Laboratory indices at hospital admission for the 233 pediatric COVID-19 patients

Here are the results of laboratory indices obtained in the first laboratory test after hospital admission (Table 2). Of the 229 cases, fifty had leukopenia. More than half of the patients (n = 150 [65.50%]) had an abnormal lymphocyte



For peer review only - http://bmjopen.bmj.com/site/about/guidelines.xhtml

percentage; 52 (22.70%) had lymphopenia. In addition, some patients were diagnosed with abnormal liver function, as indicated by 69 (30.70%) patients with elevated AST levels, while 20 (8.80%) patients had elevated ALT levels and 41 (20.20%) had increased procalcitonin levels. We also detected renal damage in some patients, as indicated by elevated levels of creatinine (n = 12 [5.40%]) and urea (n = 11 [5.00%]). Some patients exhibited elevated levels of LDH (n = 36 [18.30%]) and α-HBDH (n = 13 [59.10%]). Levels of infection-related indices, such as IL-6 (35 [24.10%]), CRP (40 [19.00%]), and ESR (19 [17.00%]), were also elevated in some patients.

For peer review only



For peer review only - http://bmjopen.bmj.com/site/about/guidelines.xhtml

**Table 2. Laboratory Indices at Hospital Admission of Pediatric Patients with COVID-19 Pneumonia**

| Tests in study population | Reference values | No. of patients tested | Median (IQR[l]) | No. of patients with value deviation from reference (%) | |
|---|---|---|---|---|---|
| | | | | Below reference | Above reference |
| **Hematologic** | | | | | |
| White blood cells, $\times10^9$/mL | Varied with age[j] | 229 | 6.64(5.26-8.18) | 50 (22.0) | 17 (7.4) |
| Lymphocytes, % | Varied with age[j] | 229 | 42.40(33.00-55.10) | 52 (22.7) | 98 (42.8) |
| Platelets, $\times10^9$/L | 100-400 | 224 | 266.00(225.00-325.00) | 1 (0.4) | 20 (8.9) |
| Hemoglobin, g/L | Varied with age[j] | 224 | 129.00(119.80-138.20) | 16 (7.2) | 7 (3.2) |
| **Biochemical** | | | | | |
| AST[a], IU/L | 13-35 | 225 | 29.00(20.00-41.00) | 10 (4.4) | 69 (30.7) |
| ALT[b], IU/L | 7-40 | 227 | 15.50(11.00-24.75) | 16 (7.0) | 20 (8.8) |
| Albumin, g/L | 40-55 | 223 | 45.60(43.10-48.05) | 16 (7.2) | 3 (1.3) |
| Prealbumin, mg/L | 200-400 | 142 | 194.30(153.85-229.68) | 76 (53.5) | - |
| Urea, mmol/L | 2.8-7.6 | 218 | 3.97(3.23-5.06) | 28 (12.8) | 11 (5.0) |
| Creatinine, μmol/L | Varied with age[j] and sex | 225 | 36.60(26.80-48.90) | 8 (3.6) | 12 (5.4) |
| Cholinesterase, U/L | 5300-11300 | 40 | 8914.00(7690.00-10022.00) | - | 4 (10.0) |
| LDH[c], U/L | Varied with age[j] | 196 | 238.00(194.80-311.80) | 2 (1.0) | 36 (18.3) |
| α-HBDH[d], U/L | 72-182 | 22 | 185.50(162.50-231.50) | - | 13 (59.1) |
| Procalcitonin, ng/ml | < 0.1 | 186 | 0.05(0.03-0.08) | - | 41 (20.2) |
| **Infection-related indices** | | | | | |
| IL-6[e], pg/ml | < 7 | 145 | 3.63(2.36-6.85) | - | 35 (24.1) |
| CRP[f], mg/L | < 8 | 211 | - | - | 40 (19.0) |
| ESR[g], mm/h | Varied with age and sex[k] | 112 | 10.00(5.75-19.00) | - | 19 (17.0) |
| **Coagulation function** | | | | | |
| PT[h], s | 11-14.5 | 191 | 11.40(10.80-12.50) | 59 (30.9) | 6 (3.1) |
| APTT[i], s | 26-40 | 191 | 32.8(29.70-35.35) | 12 (6.3) | 25 (13.1) |
| D-dimer, ug/ml | 0-0.5 | 150 | 0.26(0.15-0.42) | - | 28 (18.7) |
| Serum troponin, ug/L | < 0.01 | 55 | - | - | 18 (30.0) |

a: aspartate aminotransferase; b: alanine aminotransferase; c: lactate dehydrogenase; d: α-hydroxybutyric dehydrogenase; e: interleukin-6; f: C-reactive protein; g: erythrocyte sedimentation rate; h: prothrombin time; i: activated partial thromboplastin time; l: interquartile range.

j: White blood cells ($\times10^9$ cells/L): <28 days old: 10.0–24.0, 29 days–3 years old, 8.0–12.0, >3 years old: 4.0–10.0; Lymphocytes (%): <28 days old: 30–40, 29 days–3 years old: 50–70, >3 years old: 30–40; Hemoglobin ($\times10^9$/L): <28 days old: 10.0–24.0, 29 days–3 years: 8.0–12.0, >3 years old: 4.0–10.0; LDH: 0–29 days old: 290–2000, 30 days–23 months old: 180–430, >23 months old: 110–290.

k: Creatinine (μmol/L): ≤2 months old: 22–90, 2 months–3 years old: 11–34, 3–15 years old: 21–65, >15 years old:



For peer review only - http://bmjopen.bmj.com/site/about/guidelines.xhtml

male: 64–104, female: 49–90; ESR (mm/h): male: 0–21, female: 0–26.

Figure 1 shows the difference between the results of the first and last laboratory tests for the 233 pediatric COVID-19 cases. The value of liver damage indices (AST [difference: 6.05 IU/L; 95%CI: 1.96–10.14 IU/L; p = 0.004]) and myocardial indices (LDH [difference, 40.33 IU/L; 95%CI, 5.11– 75.55 IU/L; p = 0.026]) were significantly decreased over the disease course. Additionally, the lymphocyte percentage (difference, -2.82%; 95%CI, -4.79% to −0.85%; p < 0.006), platelets count (difference, $-38.74\times10^9$ cells/L; 95%CI, −53.46 to $-24.02\times10^9$ cells/L; p < 0.0001), and prealbumin levels (difference: −25.15 mg/L; 95%CI: −39.41 to –10.89 mg/L; p = 0.001) were significantly increased by the final laboratory test. In order to remove age effects, the study examined the trends of different clinical indicators before and after treatment by age stratification (online supplementary Figure 2).

## Factors impacting the duration to return negative RT-PCR

Figure 2 shows the distribution in the number of days to return negative (i.e., the number of days from positive RT-PCR to negative RT-PCR results for SARS-CoV-2) in the 233 pediatric COVID-19 cases based on different classification criteria, including age, sex, and clinical type. This length of duration time from positive RT-PCR to negative for individual patients ranged from 0 to 32 days. The mean lengths of time to return negative RT-PCR for asymptomatic, mild, moderate, and severe cases were 7.86±4.26, 8.24±4.77, 8.56±3.18, and 15.25±9.57 days, respectively. Regarding patient age, the mean number of days to return negative was 7.73±7.73 and 8.66±4.76 for the groups aged less than five years old and more than five years old, respectively. Regarding patient sex, the mean length of time to return negative was 8.43±4.51 and 8.19±4.97 days in male and female patients, respectively.

We constructed Cox proportional hazards regression models to explore the factors that influenced the time to return negative. The results are shown in Table 3. After controlling for the factors of sex, age, and weight,



For peer review only - http://bmjopen.bmj.com/site/about/guidelines.xhtml

comparing the HRs for the time from positive RT-PCR to negative RT-PCR between patients who had infected family members and those who did not have infected family members yielded an HR and corresponding 95% confidence interval of 0.56 (0.41–0.79). Furthermore, comparisons of patients who had zero infected family members with those who had one, two, or at least three infected family members yielded HRs (95%CI) of 0.47 (0.32–0.69), 0.51 (0.34–0.76), and 0.56 (0.36–0.86), respectively. And a lower HR means a longer time to return negative. Regarding the clinical disease types, we found the group with severe disease had a significantly longer time to return negative RT-PCR (HR [95%CI]: 0.14 [0.03–0.59]), after controlling for demographic factors (p = 0.008), while the time from positive RT-PCR to negative of mild and moderate patients was not significantly different from that of asymptomatic patients. The symptom of the occurrence of emesis was significantly associated with the time to return negative (HR [95%CI]: 0.33 [0.14–0.78]), as was having complications (HR [95%CI]: 0.57 [0.37–0.89]). There was no significant association between use of traditional Chinese medicine (e.g, Forsythia, Honeysuckle, Isatis root) and antiviral drug and time to return negative (HR [95% CI]: 0.85 [0.64–1.13]).

For peer review only

**Table 3. Hazard Ratios of Time to Return Negative RT-PCR from COVID-19 in Cox Proportional Hazards Regression Model.**

| Risk factors | Univariate Cox regression[a] | | Multivariate Cox regression[a, b] | |
|---|---|---|---|---|
| | HR (95% CI) | *p* | HR (95% CI) | *p* |
| **Clinical characteristics** | | | | |
| Sex (versus male) | 1.10 (0.81-1.37) | 0.702 | - | - |
| Age | 0.82 (0.63-1.08) | 0.160 | - | - |
| Contact history | 1.40 (0.96-2.02) | 0.079 | 1.02(0.67-1.57) | 0.918 |
| **Clinical symptoms** | | | | |
| Fever | 0.94 (0.72-1.23) | 0.655 | 0.96(0.72-1.28) | 0.789 |
| Cold | 1.10 (0.27-4.45) | 0.890 | 1.15(0.28-4.67) | 0.844 |
| Diarrhea | 1.70 (0.97-3.13) | 0.063 | 1.56(0.85-2.90) | 0.155 |
| Emesis | 0.51 (0.24-1.11) | 0.089 | 0.33(0.14-0.78) | 0.012 |
| Nausea | 0.85 (0.60-1.20) | 0.348 | 0.76(0.53-1.10) | 0.147 |
| Running nose | 0.98 (0.63-1.54) | 0.934 | 0.94(0.58-1.53) | 0.814 |
| Coughing of phlegm | 0.94 (0.61-1.45) | 0.778 | 0.90(0.57-1.43) | 0.658 |
| Complications | 0.74 (0.50-1.08) | 0.12 | 0.57(0.37-0.89) | 0.013 |
| **Clinical type (versus asymptomatic)** | | | | |
| Mild | 0.91(0.65-1.28) | 0.596 | 0.92(0.65-1.31) | 0.663 |
| Moderate | 0.92(0.56-1.51) | 0.743 | 0.86(0.50-1.48) | 0.591 |
| Severe | 0.29(0.10-0.86) | 0.025 | 0.14(0.03-0.59) | 0.008 |
| **Household contact** | | | | |
| Family member infection | 0.65(0.48-0.89) | 0.006 | 0.56(0.41-0.79) | 0.001 |
| **Number of infectious family member** | | | | |
| 1 | 0.56(0.39-0.80) | 0.002 | 0.47(0.32-0.69) | <0.001 |
| 2 | 0.62(0.43-0.90) | 0.012 | 0.51(0.34-0.76) | 0.001 |
| ≥3 | 0.61(0.41-0.92) | 0.019 | 0.56(0.36-0.86) | 0.008 |
| **Other respiratory pathogens infection** | | | | |
| Combined with virus[c] infection | 0.92 (0.65-1.30) | 0.621 | 0.84(0.59-1.21) | 0.348 |
| Combined with bacterial infection[d] | 1.10 (0.82-1.44) | 0.574 | 1.04(0.78-1.40) | 0.771 |
| **Treatment (versus ATD)** | | | | |
| TCM[e]+ATD[f] | 0.80 (0.61-1.04) | 0.093 | 0.85(0.64-1.13) | 0.259 |

a: Results of Cox proportional hazards regression models are represented as hazard ratios and 95% confidence intervals. Wald tests were performed to calculate the *p*-values for trend.

b: Adjusted for sex, age, and weight.

c: Other detected viruses included adenovirus, influenza virus, parainfluenza virus, and respiratory syncytial virus.

d: Detected bacteria included Legionella pneumophila, Mycoplasma pneumoniae, and Chlamydia pneumoniae.

e: Traditional Chinese Medicine; f: Antiviral Drug



For peer review only - http://bmjopen.bmj.com/site/about/guidelines.xhtml

# Discussion

We found that nearly 70.4% of the pediatric COVID-19 cases were infected through close contact with family members. The pediatric COVID-19 patients in our cohort were diagnosed with mainly mild disease, followed by asymptomatic and moderate disease. The proportion of severe patients was lower compared with that generally observed in adult patients, a pattern which is consistent with other studies[18 19]. The median length of hospital stay in our pediatric COVID-19 patients was 12 days, which is similar to that reported for a pediatric COVID-19 case study in Wuhan[20]. Pediatric COVID-19 patients generally exhibited the clinical symptoms of fever, cough, nausea, and coughing of phlegm, which was consistent with other studies[21]. It has been reported that adult patients with gastrointestinal symptoms are more likely to develop severe conditions[22] which is consistent with the finding in pediatric patients. Notably, the pediatric COVID-19 cases who exhibited emesis symptom took a longer time to be clear from detectable SARS-CoV-2 (HR [95%CI]: 0.33 [0.14–0.78]).

In contrast with a similar study[10], we did find 17 asymptomatic pediatric COVID-19 patients who had abnormal X-ray or CT test results. Although the asymptomatic pediatric COVID-19 patients did not present the typical symptoms of pneumonia, the radiographic examinations were still able to provide some supportive evidence for diagnosis. Since most pediatric COVID-19 patients have mild type disease, routine CT examination is necessary to detect it because a plain X-ray examination may fail to show some lesion details, leading to a misdiagnosis or missed diagnosis[23]. We observed a tendency toward a higher detection of abnormalities in the lungs by X-ray or CT with increasingly more severe disease, evidenced by the detection of abnormalities in radiographic examinations for all the patients with moderate or severe type disease in our study ($p = <0.001$). Additionally, younger pediatric COVID-19 patients were found to be more prone to developing severe conditions ($p < 0.001$). Therefore, if they become infected with SARS-CoV-2, they would be more prone to developing a more severe condition with a more fragile immune system.



For peer review only - http://bmjopen.bmj.com/site/about/guidelines.xhtml

A small proportion of pediatric COVID-19 patients were found to have complications, although the incidence of complications in children with COVID-19 was lower than that observed in adults. Pediatric COVID-19 patients with underlying medical conditions were detected to have some abnormal clinical indices (e.g., elevated AST and procalcitonin levels and longer prothrombin time), and they took longer to return negative RT-PCR than did those without any complications (HR [95%CI]: 0.57 [0.37–0.89]). We suspected that pediatric COVID-19 patients whose physical functionality may be impaired before infection with SARS-CoV-2, e.g., by an underlying infection or other complications, will need more time to return negative from COVID-19.

Elevated levels of inflammatory factors such as IL-6 and CRP were observed in the pediatric COVID-19 patients. The reason for this finding could be that cytokine storm plays a role in the immune pathophysiology of COVID-19[23]. Similar immune mechanisms have been reported in previous influenza studies[24]. However, it would need more data to determine if cytokine storms are a major determinant of severity and probably of fatal outcome. Furthermore, some pediatric COVID-19 patients presented with abnormal coagulation function (elevated d-dimer and APTT levels). In contrast with the findings from a study of adult COVID-19 patients[25], the pediatric COVID-19 patients with these abnormalities had mostly mild or asymptomatic type disease, and there was no obvious evidence linking abnormal coagulation function with poor prognosis. Self-controlled studies of pediatric COVID-19 patients found significant increases in lymphocyte levels after treatment, suggesting that lymphocyte numbers are reduced in pediatric COVID-19 cases[10]. It has been suggested that this virus might directly infect lymphocytes, especially T lymphocytes, and initiate or promote lymphocyte cell death, resulting in lymphocyte depletion and a reduced antiviral response[26].

The Cox regression model indicated that the time from positive RT-PCR to negative is strongly influenced by the presence of SARS-CoV-2-infected individuals in their family. This finding suggests that the main transmission



For peer review only - http://bmjopen.bmj.com/site/about/guidelines.xhtml

route for COVID-19 in children is close contact with family members, which is consistent with findings from another research[27]. Therefore, social distancing and hygiene measures should be exercised in an ~~more~~ intimate transmission scenario (e.g., family settings[28]) containing a risk of higher viral load and exposure, which is also supported by the conclusions of Bielecki et al.[29] and Dalton CB et al.[30] that non-pharmaceutical measures can help reduce the probability of infection or prevent developing to more severe disease by quantitatively reduce the viral inoculum. We found 59 pediatric COVID-19 cases who was the first SARS-CoV-2-infected individual in their families, which indicated that pediatric COVID-19 patients were at times a primary source of infection, a potential causation pathway often ignored[8 31]. Because their symptoms are mainly mild and they are sometimes asymptomatic, such cases might be difficult to detect in the early stage of disease. In addition, we found that children with symptoms of emesis, the vast majority of age are below 5 years old which is a very fragile and vulnerable stage of life. On the one hand, these patients need longer time to return negative RT-PCR may be due to that they are weaker to clear the virus and their immunity system is still immature. On the other hand, the literature has also shown that the occurrence of virus infection can affect gastrointestinal function and cline to gastrointestinal symptoms[32-34] (e.g., emesis[19]).

For peer review only

This study had some limitations. Firstly, we failed to capture all asymptomatic patients as the nature of a hospital-based study. However, this failure had limited impact on the interpretation of our study because it mainly caused an underestimation of the true prevalence of asymptomatic patients which is beyond the objective of our research. Secondly, to study as large of a cohort as possible, we enrolled discharged pediatric COVID-19 patients from multiple hospitals, which can be very heterogeneous in terms of treatment and management characteristics. Possible limits due to the aspects were the use of traditional Chinese medicine and international normalize ration of PT which made the data hardly comparable with those of other nations; and caution should be also exercised in extrapolating these results to other countries, as children in China may have different baseline health conditions



For peer review only - http://bmjopen.bmj.com/site/about/guidelines.xhtml

and medical care access.

## Conclusion

In conclusion, the pediatric cases with emesis symptom, complications or with infected family members will extend the length of duration from positive RT-PCR to negative. We suspected that the infecting viral load influences disease severity that emphasized on the importance of social distancing and interventions such as masks. It's difficult to detect asymptomatic pediatric COVID-19 patients; therefore, pose a potential risk to the community.

## Footnotes

**Contributors:** Weibing Wang, Guoying Huang and Zhiping Li conceptualized and designed the study, completed the literature search, critical revised the manuscript for important intellectual content. Hua Xu, Xianfeng Wang, Jinmiao Lu, Yidie Huang, Guangfei Wang, Yanling He, Jie Chen designed the data collection instruments, collected data and are responsible for the integrity of the data, reviewed and revised the manuscript. Shuli Ma, Shangrong Zou, Yuxia Cui, Ruijie Chen, Jin Lu, Xuyuan Li coordinated and supervised data collection, conducted the initial statistical analysis of epidemiology data, reviewed and revised the manuscript. Jianbo Shao, Hong Xu, Zhixi Liu, Xiaohua Ying constructed the Cox proportional hazards regression models and conducted the further statistical analysis of the study, and drafted the manuscript.

All authors approved the final manuscript as submitted to *BMJ Open* with the signature of the corresponding author and agree to be accountable for all aspects of the work.

**Funding:** This study was funded by the Bill & Melinda Gates Foundation [grant number INV-006277]. The funder had no role in the study design, data collection, data analysis, data interpretation, or writing of the report. The corresponding author had full access to all data in the study and had final responsibility for the decision to submit



For peer review only - http://bmjopen.bmj.com/site/about/guidelines.xhtml

for publication.

**Competing interests:** Jianbo Shao, Hong Xu, Zhixi Liu, Xiaohua Ying, Hua Xu, Xianfeng Wang, Jinmiao Lu, Yidie Huang, Guangfei Wang, Yanling He, Jie Chen, Shuli Ma, Shangrong Zou, Yuxia Cui, Ruijie Chen, Jin Lu, Xuyuan Li, Zhiping Li, Guoying Huang and Weibing Wang declare that they have no conflict of interest.

**Acknowledgments:** This study was sponsored by the Bill & Melinda Gates Foundation [grant number INV-006277]. We would like to extend my sincere thanks to Professor Stuart MacLeod (University of British Columbia) for his insightful comments. We thank Katie Oakley, PhD, from Liwen Bianji, Edanz Editing China (www.liwenbianji.cn/ac), for editing the English text of a draft of this manuscript.

**Data availability statement:** The data that support the findings of this study are available from the corresponding author, Weibing Wang, upon reasonable request.

**Ethics approval**

This study was approved by the ethics committee of the National Children's Medical Center, Children's Hospital of Fudan University [No.(2020) 187], and followed the Declaration of Helsinki. Written informed consents were obtained from the guardians of the patients prior to conducting the study.

For peer review only



For peer review only - http://bmjopen.bmj.com/site/about/guidelines.xhtml

# Reference

1. WHO. WHO Coronavirus (COVID-19) Dashboard. https://covid19.who.int/ (accessed August 12, 2021).

2. WHO. PANDEMIC https://www.who.int/news-room/detail/08-04-2020-who-timeline---covid-19(accessed May 10, 2020).

3. WHO. Fact sheet of Pneumonia. https://www.who.int/en/news-room/fact-sheets/detail/pneumonia(accessed May 10, 2020).

4. NHC P. Report on the development of maternal and child health in China (2019). http://www.nhc.gov.cn/fys/s7901/201905/bbd8e2134a7e47958c5c9ef032e1dfa2.shtml(accessed May 10, 2020; in Chinese).

5. WHO. Consensus document on the epidemiology of severe acute respiratory syndrome (SARS).2003.

6. Hon KLE, Leung CW, Cheng WTF, et al. Clinical presentations and outcome of severe acute respiratory syndrome in children. *The Lancet* 2003;361(9370):1701-03. doi: 10.1016/s0140-6736(03)13364-8

7. Peiris JSM, Lai ST, Poon LLM, et al. Coronavirus as a possible cause of severe acute respiratory syndrome. *The Lancet* 2003;361(9366):1319-25. doi: 10.1016/s0140-6736(03)13077-2

8. Peiris JS, Yuen KY, Osterhaus AD, et al. The severe acute respiratory syndrome. *N Engl J Med* 2003;349(25):2431-41. doi: 10.1056/NEJMra032498 [published Online First: 2003/12/19]

9. Assiri A, Al-Tawfiq JA, Al-Rabeeah AA, et al. Epidemiological, demographic, and clinical characteristics of 47 cases of Middle East respiratory syndrome coronavirus disease from Saudi Arabia: a descriptive study. *Lancet Infect Dis* 2013;13(9):752-61. doi: 10.1016/s1473-3099(13)70204-4 [published Online First: 2013/07/31]

10. Qiu H, Wu J, Hong L, et al. Clinical and epidemiological features of 36 children with coronavirus disease 2019 (COVID-19) in Zhejiang, China: an observational cohort study. *Lancet Infect Dis* 2020 doi: 10.1016/s1473-3099(20)30198-5 [published Online First: 2020/03/30]

11. NHC P. Diagnosis and treatment guidelines for 2019 novel coronavirus pneumonia (draft version 5). http://www.nhc.gov.cn/jkj/s3577/202002/a5d6f7b8c48c451c87dba14889b30147/files/3514cb996ae24e2faf65953b4ecd0df4.pdf (access May 20, 2020; in Chinese).

12. Thygesen K, Alpert JS, Jaffe AS, et al. Fourth Universal Definition of Myocardial Infarction (2018). *Circulation* 2018;138(20):e618-e51. doi: 10.1161/CIR.0000000000000617 [published Online First: 2018/12/21]

13. MedGen. Hepatic insufficiency. https://www.ncbi.nlm.nih.gov/medgen/227013 (accessed August 1, 2021). *National Center for Biotechnology information*

14. WHO. Clinical management of severe acute respiratory infection when novel coronavirus (nCoV) infection is suspected: interim guidance. January 28, 2020.(accessed May 10, 2020).

15. Chen Z, Fu J, Shu Q, et al. Diagnosis and treatment recommendation for pediatric coronavirus disease-19. *Zhejiang Da Xue Xue Bao Yi Xue Ban* 2020;49(1):1-8. [published Online First: 2020/02/18]

16. NHC P. National Health and Health Commission of the People’s Republic of China. Diagnosis and Treatment Protocol for Novel Coronavirus Pneumonia (Trial Version 7).

17. Feng F, Dongchi Z, Yu C, et al. Diagnosis and prevention recommendations of Novel Coronavirus 2019 in peidatric patients. *Chinese Journal of Pediatrics* 2020(03):169-74. doi: 10.3760/cma.j.issn.0578-1310.2020.03.001

18. Davies NG, Klepac P, Liu Y, et al. Age-dependent effects in the transmission and control of COVID-19 epidemics. *Nat Med* 2020;26(8):1205-11. doi: 10.1038/s41591-020-0962-9 [published Online First: 2020/06/18]



For peer review only - http://bmjopen.bmj.com/site/about/guidelines.xhtml

19. Cui X, Zhao Z, Zhang T, et al. A systematic review and meta-analysis of children with coronavirus disease 2019 (COVID-19). *J Med Virol* 2021;93(2):1057-69. doi: 10.1002/jmv.26398 [published Online First: 2020/08/08]
20. Xia W, Shao J, Guo Y, et al. Clinical and CT features in pediatric patients with COVID-19 infection: Different points from adults. *Pediatr Pulmonol* 2020;55(5):1169-74. doi: 10.1002/ppul.24718 [published Online First: 2020/03/07]
21. Ng DC, Tan KK, Chin L, et al. Clinical and epidemiological characteristics of children with COVID-19 in Negeri Sembilan, Malaysia. *Int J Infect Dis* 2021;108:347-52. doi: 10.1016/j.ijid.2021.05.073 [published Online First: 2021/06/05]
22. Wan Y, Li J, Shen L, et al. Enteric involvement in hospitalised patients with COVID-19 outside Wuhan. *Lancet Gastroenterol Hepatol* 2020 doi: 10.1016/s2468-1253(20)30118-7 [published Online First: 2020/04/19]
23. Li H, Liu L, Zhang D, et al. SARS-CoV-2 and viral sepsis: observations and hypotheses. *The Lancet* 2020 doi: 10.1016/s0140-6736(20)30920-x
24. Iwasaki A, Pillai PS. Innate immunity to influenza virus infection. *Nat Rev Immunol* 2014;14(5):315-28. doi: 10.1038/nri3665 [published Online First: 2014/04/26]
25. Zhou F, Yu T, Du R, et al. Clinical course and risk factors for mortality of adult inpatients with COVID-19 in Wuhan, China: a retrospective cohort study. *Lancet* 2020;395(10229):1054-62. doi: 10.1016/s0140-6736(20)30566-3 [published Online First: 2020/03/15]
26. Hamming I, Timens W, Bulthuis ML, et al. Tissue distribution of ACE2 protein, the functional receptor for SARS coronavirus. A first step in understanding SARS pathogenesis. *J Pathol* 2004;203(2):631-7. doi: 10.1002/path.1570 [published Online First: 2004/05/14]
27. Su L, Ma X, Yu H, et al. The different clinical characteristics of corona virus disease cases between children and their families in China - the character of children with COVID-19. *Emerg Microbes Infect* 2020;9(1):707-13. doi: 10.1080/22221751.2020.1744483 [published Online First: 2020/03/27]
28. Kim J, Choe YJ, Lee J, et al. Role of children in household transmission of COVID-19. *Arch Dis Child* 2021;106(7):709-11. doi: 10.1136/archdischild-2020-319910 [published Online First: 2020/08/10]
29. Bielecki M, Zust R, Siegrist D, et al. Social distancing alters the clinical course of COVID-19 in young adults: A comparative cohort study. *Clin Infect Dis* 2020 doi: 10.1093/cid/ciaa889 [published Online First: 2020/07/01]
30. Dalton CB, Corbett SJ, Katelaris AL. COVID-19: implementing sustainable low cost physical distancing and enhanced hygiene. *Med J Aust* 2020;212(10):443-46 e1. doi: 10.5694/mja2.50602 [published Online First: 2020/05/02]
31. Jiehao C, Jin X, Daojiong L, et al. A Case Series of Children With 2019 Novel Coronavirus Infection: Clinical and Epidemiological Features. *Clin Infect Dis* 2020;71(6):1547-51. doi: 10.1093/cid/ciaa198 [published Online First: 2020/03/01]
32. Chiappini E, Licari A, Motisi MA, et al. Gastrointestinal involvement in children with SARS-COV-2 infection: An overview for the pediatrician. *Pediatr Allergy Immunol* 2020;31 Suppl 26:92-95. doi: 10.1111/pai.13373 [published Online First: 2020/11/26]
33. Parasa S, Desai M, Thoguluva Chandrasekar V, et al. Prevalence of Gastrointestinal Symptoms and Fecal Viral Shedding in Patients With Coronavirus Disease 2019: A Systematic Review and Meta-analysis. *JAMA Netw Open* 2020;3(6):e2011335. doi: 10.1001/jamanetworkopen.2020.11335 [published Online First: 2020/06/12]
34. Andrews PLR, Cai W, Rudd JA, et al. COVID-19, nausea, and vomiting. *J Gastroenterol Hepatol*



For peer review only - http://bmjopen.bmj.com/site/about/guidelines.xhtml

2021;36(3):646-56. doi: 10.1111/jgh.15261 [published Online First: 2020/09/22]

## Figure Legend：

**Figure 1 Comparing the clinical indicators of pediatric COVID-19 patients pre-therapy and post-treatment during hospitalization**

The effect of treatment in 233 pediatric COVID-19 patients. The red and green boxes represent the values of five clinical indicators before and after treatment, respectively. The scattered points on each box represent the values of each clinical indicator for each patient. The middle line of the box represents the medium value of the group. The height of box represents the interquartile range (IQR) of each group. The *p*-value is calculated based on the paired Student's *t*-test.

* represents a *p*-value of less than 0.05; ** represents a *p*-value of less than 0.005; *** represents a *p*-value of less than 0.001.

**Figure 2. Distribution of the time to return negative of the 233 pediatric COVID-19 patients**

The distribution of the mean time to return negative RT-PCR for the 233 pediatric COVID-19 patients during the period of admission, split in groups based on different classification criteria. The gradient blue bars represent the mean number of days to return negative at age ≤5 years and age >5 years. The purple and pink bars represent mean number of days to return negative for male and female patients, respectively. The four bars on the right represent the mean number of days to return negative with the four clinical types of COVID-19. The scattered points on each bar represent the number of days to return negative for each patient in the group. The error lines represent the mean value plus or minus a standard deviation. Turning negative was defined as the time at which a confirmed COVID-19 patient first received negative results for an RT-PCR test for SARS-CoV-2.

For peer review only



For peer review only - http://bmjopen.bmj.com/site/about/guidelines.xhtml

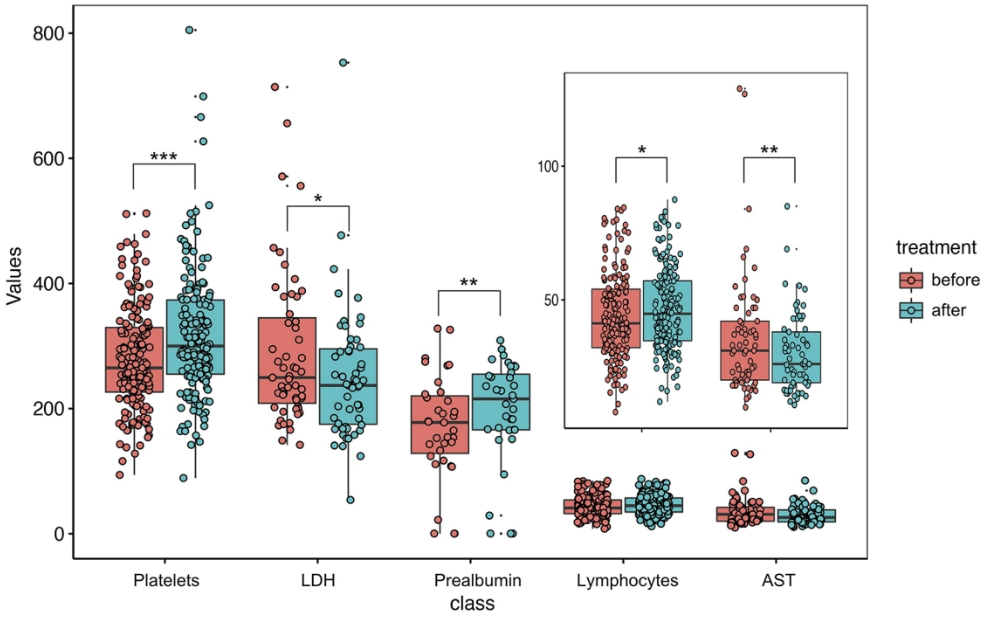


Figure 1 Comparing the clinical indicators of pediatric COVID-19 patients pre-therapy and post-treatment during hospitalization / The effect of treatment in 233 pediatric COVID-19 patients. The red and green boxes represent the values of five clinical indicators before and after treatment, respectively. The scattered points on each box represent the values of each clinical indicator for each patient. The middle line of the box represents the medium value of the group. The height of box represents the interquartile range (IQR) of each group. The p-value is calculated based on the paired Student's t-test.* represents a p-value of less than 0.05; ** represents a p-value of less than 0.005; *** represents a p-value of less than 0.001.

129x81mm (350 x 350 DPI)

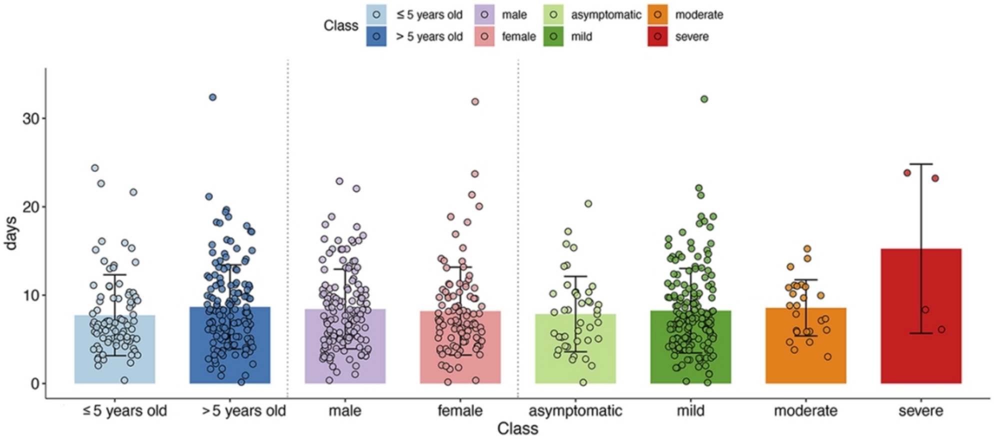


Figure 2. Distribution of the time to return negative of the 233 pediatric COVID-19 patients
The distribution of the mean time to return negative RT-PCR for the 233 pediatric COVID-19 patients during the period of admission, split in groups based on different classification criteria. The gradient blue bars represent the mean number of days to return negative at age ≤5 years and age >5 years. The purple and pink bars represent mean number of days to return negative for male and female patients, respectively. The four bars on the right represent the mean number of days to return negative with the four clinical types of COVID-19. The scattered points on each bar represent the number of days to return negative for each patient in the group. The error lines represent the mean value plus or minus a standard deviation. Turning negative was defined as the time at which a confirmed COVID-19 patient first received negative results for an RT-PCR test for SARS-CoV-2.

109x49mm (300 x 300 DPI)

# Title: Factors associated with the time to return negative RT-PCR from COVID-19 in pediatric patients: a retrospective cohort study

## Authors and Institutions:

Jianbo Shao[a, †], Hong Xu[b, †], Zhixi Liu[c, †], Xiaohua Ying[c, †], Hua Xu[d], Xianfeng Wang[e], Jinmiao Lu[f], Yidie Huang[f], Guangfei Wang[f], Yanling He[g], Jie Chen[h], Shuli Ma[i], Shangrong Zou[j], Yuxia Cui[k], Ruijie Chen[l], Jin Lu[m], Xuyuan Li[n], Zhiping Li[f, *], Guoying Huang [f, *], Weibing Wang [c, *]

[a]Department of Radiological Medicine, Wuhan Children's Hospital (Wuhan Maternal and Child Healthcare Hospital), Tongji Medical College, Huazhong University of Science & Technology, Wuhan 430016, China; and [b]Department of Nephrology, National Children's Medical Center, Children's Hospital of Fudan University, Shanghai 201102, China; and [c]School of Public Health, Fudan University, Shanghai 200032, China; and [d]Department of Pharmacy, Wuhan Children's Hospital (Wuhan Maternal and Child Healthcare Hospital), Tongji Medical College, Huazhong University of Science & Technology, Wuhan 430016, China; and [e]Department of Pediatrics, The Third People's Hospital of Shenzhen, Shenzhen 518020, China; and [f]Department of Clinical Pharmacy, National Children's Medical Center, Children's Hospital of Fudan University, Shanghai 201102, China; and [g]Department of Pharmacy, Guangzhou Women and Children's Medical Center, Guangzhou 510623, China; and [h]Department of Pharmacy, The Third People's Hospital of Kunming, Kunming 650000, China; and [i]Department of Pharmacy, Children's Hospital Affiliated to Zhengzhou University, Zhengzhou 450000, China; and [j]Department of Pharmacy, Guangzhou Eighth People's Hospital, Guangzhou 510060, China; and [k]Department of Pediatrics, Guizhou Provincial People's Hospital, Guiyang 550002, China; and [l]Wenzhou Municipal Key Laboratory of Paediatric Pharmacy, Department of Pharmacy, The Second

For peer review only

Affiliated Hospital and Yuying Children's Hospital of Wenzhou Medical University, Wenzhou 325027, China; and [m]Department of Pharmacy, The First Affiliated Hospital of USTC, Division of Life Sciences and Medicine, University of Science and Technology of China, Anhui 230001, China; and [n]Department of Biology, University of California, Santa Barbara 93101, California, the United States

[†] Jianbo Shao, Hong Xu, Zhixi Liu, Xiaohua Ying contributed equally to this manuscript

[*] Zhiping Li, Guoying Huang, Weibing Wang contributed equally to corresponding work of this manuscript

## Corresponding author:

Dr. Zhiping Li, PhD, Department of Clinical Pharmacy, National Children's Medical Center, Children's Hospital of Fudan University, Shanghai 201102, China

Email: zpli@fudan.edu.cn

Dr. Guoying Huang*, MD, PhD, Centre of Cardiovascular, National Children's Medical Center, Children's Hospital of Fudan University, Shanghai 201102, China

Email: gyhuang@shmu.edu.cn

Dr. Weibing Wang, PhD, Department of Epidemiology, School of Public Health, Fudan University, 138 Yi Xue Yuan Road, Shanghai 200032, China, 86-21-54237811

Email: wwb@fudan.edu.cn

## Contents:

Supplementary Table 1. Definitions of clinical types for COVID-19 pediatric patients
Supplementary Figure 1. The spatial distribution of the 12 COVID-19 tertiary hospitals
Supplementary Figure 2. Comparison of clinical indicators stratified by age pre-therapy and post-treatment

For peer review only

**Supplementary Table 1. Definitions of clinical types for COVID-19 pediatric patients**

| Classification of clinical types | Based on the clinical characteristics of confirmed COVID-19 pediatric cases |
|---|---|
| Asymptomatic disease | • No clinical symptoms or signs<br>• Normal chest imaging examination<br>• Positive RT-PCR test for SARS-CoV-2 or positive result for retrospectively diagnosed sero-specific antibody |
| Mild disease | • Acute upper respiratory tract infection (*e.g.*, fever, fatigue, myalgia, cough, sore throat, runny nose and sneezing, *etc.*)<br>• Pharyngeal congestion<br>• No positive signs of lungs<br>• Some may have no fever, or only other gastrointestinal symptoms (*e.g.*, nausea, vomiting, abdominal pain and diarrhea) |
| Moderate disease | • Pneumonia<br>• Clinical symptoms such as fever, cough<br>• Wheezing, dry and (or) moist rales were heard in the lungs<br>• Sub-clinical: some without clinical symptoms or signs but with abnormal pulmonary lesions |
| Severe disease | • Early symptoms of respiratory tract (*e.g.*, fever and cough, *etc*); may be accompanied by digestive tract symptoms (e.g., diarrhea, *etc*)<br>• Progression of disease within a week or so, with dyspnea, central cyanosis, hypoxia, pulse oximetry <0.92 |
| Critical disease | • ARDS<br>• Respiratory failure<br>• Multiple organ dysfunction (*e.g.*, shock, encephalopathy, myocardial injury or failure, coagulation dysfunction and acute kidney injury) |

For peer review only

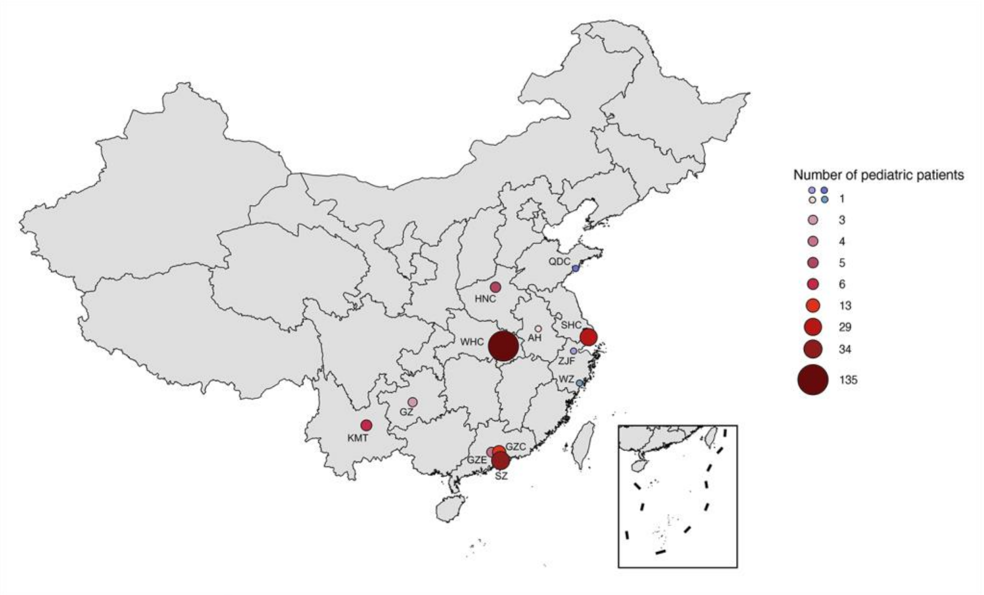


**Supplementary Figure 1. The spatial distribution of the 12 COVID-19 tertiary hospitals**

The number of patients enrolled in the study is varied by the size of the points and the hospitals varied by the color of the points. Abbreviations: WHC: Wuhan Children's Hospital (Wuhan Maternal and Child Healthcare Hospital), Tongji Medical College, Huazhong University of Science & Technology; SZ: The Third People's Hospital of Shenzhen; SHC: National Children's Medical Center, Children's Hospital of Fudan University; GZC: Guangzhou Women and Children's Medical Center; KMT: The Third People's Hospital of Kunming; HNC: Children's Hospital Affiliated to Zhengzhou University; GZE: Guangzhou Eighth People's Hospital; GZ: Guizhou Provincial People's Hospital; QDC: Qingdao Children's Hospital; AH: The First Affiliated Hospital of USTC, Division of Life Sciences and Medicine, University of Science and Technology of China; ZJF: The First Hospital of Zhejiang University and WZ: The Second Affiliated Hospital and Yuying Children's Hospital of Wenzhou Medical University.

For peer review only

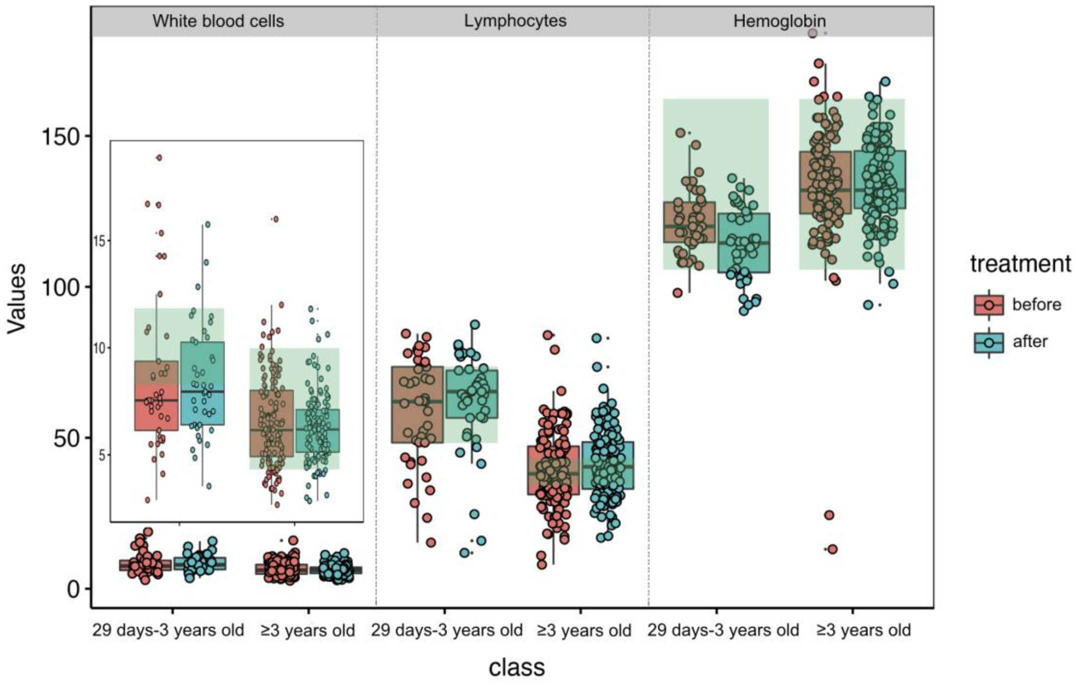


**Supplementary Figure 2. Comparison of clinical indicators stratified by age pre-therapy and post-treatment**

The red and green boxes represent the clinical indicator values before and after treatment, respectively. The scattered points on each box represent the values of each clinical indicator for each patient. The shaded green rectangle represents the normal reference range for an indicator for that age group. The middle line of the box represents the medium value of the group. The height of the box represents the interquartile range (IQR) of each group.

**STROBE 2007 (v4) Statement—Checklist of items that should be included in reports of *cohort studies***

| Section/Topic | Item # | Recommendation | Reported on page # |
|---|---|---|---|
| **Title and abstract** | 1 | (*a*) Indicate the study's design with a commonly used term in the title or the abstract | 1, 3 |
| | | (*b*) Provide in the abstract an informative and balanced summary of what was done and what was found | 3-5 |
| **Introduction** | | | 6 |
| Background/rationale | 2 | Explain the scientific background and rationale for the investigation being reported | 6 |
| Objectives | 3 | State specific objectives, including any prespecified hypotheses | 6 |
| **Methods** | | | 6-9 |
| Study design | 4 | Present key elements of study design early in the paper | 6-7 |
| Setting | 5 | Describe the setting, locations, and relevant dates, including periods of recruitment, exposure, follow-up, and data collection | 6-7 |
| Participants | 6 | (*a*) Give the eligibility criteria, and the sources and methods of selection of participants. Describe methods of follow-up | 6-7 |
| | | (*b*) For matched studies, give matching criteria and number of exposed and unexposed | Not Applicable |
| Variables | 7 | Clearly define all outcomes, exposures, predictors, potential confounders, and effect modifiers. Give diagnostic criteria, if applicable | 7-8 |
| Data sources/ measurement | 8* | For each variable of interest, give sources of data and details of methods of assessment (measurement). Describe comparability of assessment methods if there is more than one group | 7-8 |
| Bias | 9 | Describe any efforts to address potential sources of bias | Not Applicable |
| Study size | 10 | Explain how the study size was arrived at | 6-7 |
| Quantitative variables | 11 | Explain how quantitative variables were handled in the analyses. If applicable, describe which groupings were chosen and why | 8 |
| Statistical methods | 12 | (*a*) Describe all statistical methods, including those used to control for confounding | 8-9 |
| | | (*b*) Describe any methods used to examine subgroups and interactions | 8-9 |
| | | (*c*) Explain how missing data were addressed | 9 |
| | | (*d*) If applicable, explain how loss to follow-up was addressed | Not Applicable |
| | | (*e*) Describe any sensitivity analyses | Not Applicable |
| **Results** | | | 9-16 |

For peer review only

| | | | |
|---|---|---|---|
| Participants | 13* | (a) Report numbers of individuals at each stage of study—eg numbers potentially eligible, examined for eligibility, confirmed eligible, included in the study, completing follow-up, and analysed | 9-11 |
| | | (b) Give reasons for non-participation at each stage | Not Applicable |
| | | (c) Consider use of a flow diagram | Not Applicable |
| Descriptive data | 14* | (a) Give characteristics of study participants (eg demographic, clinical, social) and information on exposures and potential confounders | 9-11 |
| | | (b) Indicate number of participants with missing data for each variable of interest | 9-14 |
| | | (c) Summarise follow-up time (eg, average and total amount) | 9 |
| Outcome data | 15* | Report numbers of outcome events or summary measures over time | 9-16 |
| Main results | 16 | (*a*) Give unadjusted estimates and, if applicable, confounder-adjusted estimates and their precision (eg, 95% confidence interval). Make clear which confounders were adjusted for and why they were included | 9-16 |
| | | (*b*) Report category boundaries when continuous variables were categorized | 9-10 |
| | | (*c*) If relevant, consider translating estimates of relative risk into absolute risk for a meaningful time period | Not Applicable |
| Other analyses | 17 | Report other analyses done—eg analyses of subgroups and interactions, and sensitivity analyses | Not Applicable |
| **Discussion** | | | 17-20 |
| Key results | 18 | Summarise key results with reference to study objectives | 17 |
| **Limitations** | | | 20 |
| Interpretation | 20 | Give a cautious overall interpretation of results considering objectives, limitations, multiplicity of analyses, results from similar studies, and other relevant evidence | 20 |
| Generalisability | 21 | Discuss the generalisability (external validity) of the study results | 20 |
| **Other information** | | | 20-22 |
| Funding | 22 | Give the source of funding and the role of the funders for the present study and, if applicable, for the original study on which the present article is based | 21 |

*Give information separately for cases and controls in case-control studies and, if applicable, for exposed and unexposed groups in cohort and cross-sectional studies.

**Note:** An Explanation and Elaboration article discusses each checklist item and gives methodological background and published examples of transparent reporting. The STROBE checklist is best used in conjunction with this article (freely available on the Web sites of PLoS Medicine at http://www.plosmedicine.org/, Annals of Internal Medicine at http://www.annals.org/, and Epidemiology at http://www.epidem.com/). Information on the STROBE Initiative is available at www.strobe-statement.org.

For peer review only

BMJ Open

# Factors associated with the time to return negative RT-PCR from COVID-19 in pediatric patients: a retrospective cohort study

| | |
|---|---|
| Journal: | *BMJ Open* |
| Manuscript ID | bmjopen-2021-052609.R2 |
| Article Type: | Original research |
| Date Submitted by the Author: | 17-Sep-2021 |
| Complete List of Authors: | Shao, Jianbo; Huazhong University of Science and Technology Tongji Medical College Affiliated Wuhan Children's Hospital<br>Xu, Hong; Children's Hospital of Fudan University, Pediatric Nephrology<br>Liu, Zhixi; Fudan University School of Public Health<br>YING, Xiaohua; Fudan University School of Public Health<br>Xu, Hua; Huazhong University of Science and Technology Tongji Medical College Affiliated Wuhan Children's Hospital, Department of Pharmacy<br>Wang, Xianfeng; The Third People's Hospital of Shenzhen<br>Lu, Jinmiao; Children's Hospital of Fudan University, Department of Clinical Pharmacy<br>Huang, Yidie; Children's Hospital of Fudan University, Department of Clinical Pharmacy<br>Wang, Guangfei; Children's Hospital of Fudan University, Department of Clinical Pharmacy<br>He, Yanling; Guangzhou Women and Children's Medical Center, Department of Pharmacy<br>Chen, Jie; Dali University, Affiliated Sixth People's Hospital<br>Ma, Shuli; Zhengzhou University, Affiliated Children’s Hospital<br>Zou, Shangrong; Guangzhou Eighth People's Hospital<br>Cui, Yuxia; Guizhou Provincial People's Hospital<br>Chen, Ruijie; Yuying Children's Hospital of Wenzhou Medical College<br>Lu, Jin; University of Science and Technology of China<br>Li, Xuyuan; University of California Santa Barbara<br>Li, Zhiping; Children's Hospital of Fudan University, Department of Clinical Pharmacy<br>Huang, Guoying; Children's Hospital of Fudan University, Department of Clinical Pharmacy<br>Wang, Weibing; Fudan University School of Public Health, Department of Pediatrics |
| <b>Primary Subject Heading</b>: | Paediatrics |
| Secondary Subject Heading: | Epidemiology, Infectious diseases |
| Keywords: | COVID-19, Epidemiology < INFECTIOUS DISEASES, PAEDIATRICS |
| | |

SCHOLARONE™
Manuscripts

*I, the Submitting Author has the right to grant and does grant on behalf of all authors of the Work (as defined in the below author licence), an exclusive licence and/or a non-exclusive licence for contributions from authors who are: i) UK Crown employees; ii) where BMJ has agreed a CC-BY licence shall apply, and/or iii) in accordance with the terms applicable for US Federal Government officers or employees acting as part of their official duties; on a worldwide, perpetual, irrevocable, royalty-free basis to BMJ Publishing Group Ltd ("BMJ") its licensees and where the relevant Journal is co-owned by BMJ to the co-owners of the Journal, to publish the Work in this journal and any other BMJ products and to exploit all rights, as set out in our licence.*

*The Submitting Author accepts and understands that any supply made under these terms is made by BMJ to the Submitting Author unless you are acting as an employee on behalf of your employer or a postgraduate student of an affiliated institution which is paying any applicable article publishing charge ("APC") for Open Access articles. Where the Submitting Author wishes to make the Work available on an Open Access basis (and intends to pay the relevant APC), the terms of reuse of such Open Access shall be governed by a Creative Commons licence – details of these licences and which Creative Commons licence will apply to this Work are set out in our licence referred to above.*

*Other than as permitted in any relevant BMJ Author's Self Archiving Policies, I confirm this Work has not been accepted for publication elsewhere, is not being considered for publication elsewhere and does not duplicate material already published. I confirm all authors consent to publication of this Work and authorise the granting of this licence.*

For peer review only

# Title: Factors associated with the time to return negative RT-PCR from COVID-19 in pediatric patients: a retrospective cohort study

## Authors and Institutions:

Jianbo Shao[a, †], Hong Xu[b, †], Zhixi Liu[c, †], Xiaohua Ying[c, †], Hua Xu[d], Xianfeng Wang[e], Jinmiao Lu[f], Yidie Huang[f], Guangfei Wang[f], Yanling He[g], Jie Chen[h], Shuli Ma[i], Shangrong Zou[j], Yuxia Cui[k], Ruijie Chen[l], Jin Lu[m], Xuyuan Li[n], Zhiping Li[f, *], Guoying Huang[f, *], Weibing Wang[c, *]

[a]Department of Radiological Medicine, Wuhan Children's Hospital (Wuhan Maternal and Child Healthcare Hospital), Tongji Medical College, Huazhong University of Science & Technology, Wuhan 430016, China; and [b]Department of Nephrology, National Children's Medical Center, Children's Hospital of Fudan University, Shanghai 201102, China; and [c]School of Public Health, Fudan University, Shanghai 200032, China; and [d]Department of Pharmacy, Wuhan Children's Hospital (Wuhan Maternal and Child Healthcare Hospital), Tongji Medical College, Huazhong University of Science & Technology, Wuhan 430016, China; and [e]Department of Pediatrics, The Third People's Hospital of Shenzhen, Shenzhen 518020, China; and [f]Department of Clinical Pharmacy, National Children's Medical Center, Children's Hospital of Fudan University, Shanghai 201102, China; and [g]Department of Pharmacy, Guangzhou Women and Children's Medical Center, Guangzhou 510623, China; and [h]Department of Pharmacy, The Third People's Hospital of Kunming, Kunming 650000, China; and [i]Department of Pharmacy, Children's Hospital Affiliated to Zhengzhou University, Zhengzhou 450000, China; and [j]Department of Pharmacy, Guangzhou Eighth People's Hospital, Guangzhou 510060, China; and [k]Department of Pediatrics, Guizhou Provincial People's Hospital, Guiyang 550002, China; and [l]Wenzhou Municipal Key Laboratory of Paediatric Pharmacy, Department of Pharmacy, The Second Affiliated Hospital and Yuying Children's Hospital of Wenzhou Medical University, Wenzhou 325027, China; and [m]Department of Pharmacy,



For peer review only - http://bmjopen.bmj.com/site/about/guidelines.xhtml

For peer review only

The First Affiliated Hospital of USTC, Division of Life Sciences and Medicine, University of Science and Technology of China, Anhui 230001, China; and [n]Department of Biology, University of California, Santa Barbara 93101, California, the United States

† Jianbo Shao, Hong Xu, Zhixi Liu, Xiaohua Ying contributed equally to this manuscript

* Zhiping Li, Guoying Huang, Weibing Wang contributed equally to corresponding work of this manuscript

## Corresponding author:

Dr. Zhiping Li, PhD, Department of Clinical Pharmacy, National Children's Medical Center, Children's Hospital of Fudan University, Shanghai 201102, China

Email: zpli@fudan.edu.cn

Dr. Guoying Huang*, MD, PhD, Centre of Cardiovascular, National Children's Medical Center, Children's Hospital of Fudan University, Shanghai 201102, China

Email: gyhuang@shmu.edu.cn

Dr. Weibing Wang, PhD, Department of Epidemiology, School of Public Health, Fudan University, 138 Yi Xue Yuan Road, Shanghai 200032, China, 86-21-54237811

Email: wwb@fudan.edu.cn

Word count: 3294

For peer review only



For peer review only - http://bmjopen.bmj.com/site/about/guidelines.xhtml

# Abstract

**Objective:** This study aimed to describe the epidemiological and clinical features and potential factors related to the time to return negative RT-PCR in discharged pediatric patients with COVID-19.

**Design:** Retrospective cohort study

**Setting:** Unscheduled admissions to 12 tertiary hospitals in China.

**Participants:** Two hundred and thirty-three clinical charts of pediatric patients with confirmed diagnosis of COVID 19 admitted from January 1, 2020 to April 17, 2020.

**Primary and secondary outcome measures:** Primary outcome measures: factors associated with the time to return negative RT-PCR from COVID-19 in pediatric patients. Secondary outcome measures: epidemiological and clinical features and laboratory results in pediatric patients.

**Results** The median age of patients in our cohort was 7·50 (IQR: 2·92,12·17) years and 133 (57·1%) patients were male. 42 (18.0%) patients were evaluated as asymptomatic, while 162 (69.5%) and 25 (10.7%) patients were classified as mild or moderate, respectively. In Cox regression analysis, In Cox regression analysis, longer time to negative RT-PCR was associated with the presence of confirmed infection in family members (Hazard Ratio (HR) [95%CI]: 0.56 [0.41–0.79]). Pediatric patients with emesis symptom had a longer time to return negative (HR [95%CI]: 0.33 [0.14–0.78]). During hospitalization, the use of traditional Chinese medicine (TCM) and antiviral drugs at the same time is not more conducive to return negative than antiviral drugs alone (HR [95%CI]: 0.85[0.64-1.13]).

**Conclusions** The mode of transmission might be a critical factor determining the disease severity of COVID-19. Patients with emesis symptom, complications or confirmed infection in family members may have longer healing times than others. However, no significant favorable effects from TCM when the patients have received antiviral treatment.




For peer review only - http://bmjopen.bmj.com/site/about/guidelines.xhtml

**Keywords** SARS-CoV-2; COVID-19; infectious disease; pediatrics

For peer review only



For peer review only - http://bmjopen.bmj.com/site/about/guidelines.xhtml

## Strengths and limitations of this study

- This study enrolled all pediatric COVID-19 patients (n = 233) discharged from 12 tertiary hospitals across 11 cities and found pediatric patients with emesis symptom had a longer time to return negative.
- We determined the in-family transmission is the main infection route for pediatric COVID-19 cases.
- Transmission mode may be key factors determining the clinical course of COVID-19.

For peer review only



For peer review only - http://bmjopen.bmj.com/site/about/guidelines.xhtml

# Introduction

Coronavirus disease 2019 (COVID-19) is an emerging infectious disease with high transmissibility. As of 11 August 2021, the cumulative number of cases worldwide has reached 203,944,144 with 121,960 of those cases in China[1]. On March 11, 2021, the World Health Organization (WHO) further announced the epidemic of COVID-19 would be classified as a global pandemic[2]. According to the data released by the WHO[3], pneumonia is the leading cause of death among children worldwide and the second among children under five in China[4]. Pediatric cases were reported during the epidemics of severe acute respiratory syndrome coronavirus (SARS-CoV)[5 6]. In pediatric SARS patients, the clinical symptoms of teenagers were similar to those of adults[7]. Furthermore, they maintained stable conditions throughout the disease course and had good prognoses[8]. Another study demonstrated that pediatric cases accounted for only 2% of all Middle East Respiratory Syndrome cases[9]. One previous study that enrolled 36 COVID-19 pediatric cases concluded that the close contacts with family members might be difficult to avoid in daily life[10].

The low proportion of pediatric patients among COVID-19 cases has perplexed clinicians, epidemiologists, and scientists. Current understanding regarding the duration of positive RT-PCR to negative for COVID-19 is limited, and this knowledge gap is particularly notable among children. Therefore, we aimed to explore and analyze the clinical and epidemiological characteristics of 233 discharged pediatric patients to reveal corresponding information for the prevention and treatment of pediatric COVID-19 patients.

# Materials and Methods

## Study population

This multi-center retrospective study was conducted in 12 tertiary hospitals in 11 Chinese cities. We collected information about the discharged pediatric COVID-19 patients treated in these hospitals from January 1, 2020 to



For peer review only - http://bmjopen.bmj.com/site/about/guidelines.xhtml

April 17, 2020. The pediatric patients with confirmed COVID-19 were diagnosed based on guidelines issued by the National Health Commission of the People's Republic of China[11].

## Procedure

Relevant medical information was collected from electronic medical records and reviewed by trained doctors to ensure the quality of the medical data. Retrieved information included the demographic information, COVID-19 contact history (e.g., history of travel to or residence in the COVID-19 epidemic areas or history of contact with SARS-CoV-2-infected individuals), complications (*e.g.*, myocardial injury[12]: the elevation of cardiac troponin, with at least one value above the 99th percentile upper reference limit; liver insufficiency[13]: the inability of the liver to perform its normal synthetic and metabolic functions with abnormal values of biochemical indicators aspartate aminotransferase (AST), alanine aminotransferase (ALT), *etc.* The ranges of normal values show in Table 2.), laboratory tests results, chest computed tomography and B-scan ultrasonography results. Laboratory tests included blood routine examination and examinations for lung, liver, myocardial, and kidney function, such as measurements of the AST, ALT, lactate dehydrogenase (LDH), α-hydroxybutyrate dehydrogenase (α-HBDH) and the erythrocyte sedimentation rate (ESR).

The preliminary diagnosis of suspected COVID-19 was based on clinical symptoms and potential exposure via travel to COVID-19 epidemic areas or close contact with a confirmed case. The diagnosis was subsequently confirmed based on results from real-time reverse transcriptase-polymerase chain reaction (RT-PCR) tests for SARS-CoV-2 (severe acute respiratory syndrome coronavirus 2) on nasal or pharyngeal swab specimens from all suspected patients diagnosed by the previous step. RT-PCR is recommended by the Center for Disease Control and Prevention (CDC)[11] and WHO interim guidance as a solid method for confirming COVID-19 diagnosis[14]. RT-PCR testing was performed to detect infection with other viruses (i.e., adenovirus, influenza virus, parainfluenza virus,



For peer review only - http://bmjopen.bmj.com/site/about/guidelines.xhtml

and respiratory syncytial virus) and performed sputum culture to test for potential infections with bacteria, including *Legionella pneumophila*, *Mycoplasma pneumoniae*, and *Chlamydia pneumoniae*. We evaluated the clinical type of each admitted pediatric COVID-19 patient according to the recommendations issued by the pediatrics branch of the Chinese Medical Association[15]. Patients were described as having one of four clinical types of COVID-19, i.e., asymptomatic, mild, moderate and severe disease, based on their RT-PCR results, clinical symptoms and radiological results (online supplementary Table 1)[16 17]. Once admitted, these pediatric patients were given available antiviral treatments (e.g., Ribavirin aerosol, Oseltamivir granule, etc.) and oxygen if necessary (blood oxygen saturation <0.92). The discharge criteria were that the patient must have obtained two consecutive negative RT-PCR test results.

## Statistical analysis

Categorical variables are presented as number and frequency rate using the Fisher exact test or Chi-square test. Continuous variables with non-normal distribution are presented as the median and interquartile range (IQR) and were compared using the Kruskal-Wallis Rank Sum Test. In addition, we analyzed the first and last laboratory test results to examine the effect of treatment during hospitalization. These two test results were compared by a paired Student's t-test or Wilcoxon's signed rank test.

Cox proportional hazards regression models were used to estimate hazard ratios (HR) and corresponding 95% confidence intervals (CI) to explore associations between risk factors and the number of days to return negative RT-PCR for the 233 pediatric COVID-19 cases. In Cox regression models, the interpretation of results is that the endpoint event we observed was negative RT-PCR (benign event); if the HR value of a variable is less than 1, the variable is not conducive to the occurrence of the outcome of negative RT-PCR, and there will be a longer day to return negative, and vice versa. Time to return negative was defined as the time from positive RT-PCR results to



For peer review only - http://bmjopen.bmj.com/site/about/guidelines.xhtml

negative RT-PCR results for SARS-CoV-2 during the hospitalization. During the analysis, sample size varied because of missing data; the sample sizes for each analysis are listed in Table 1. The variables assessed in the statistical analysis were contact history, clinical symptoms, and clinical indicators. Univariate and multivariate analyses adjusted for sex, age, and weight were carried out by Cox proportional hazard ratio models to examine significant associations. These analyses conducted by R-packages 'dplyr', a data manipulation package, will provide any information about factors that contribute to a longer duration of positive RT-PCR to negative; 'survival', 'survminer' helped to determine the risk factors that contributed to a longer time of positive RT-PCR.

All analyses were conducted on the subjects who lacked missing data, and only differences with two-sided α and a p-value less than 0.05 was considered statistically significant. All analyses were conducted with the use of R software version 3.6.2.

### Patient and Public Involvement

Patients and/or the public were not involved in the design, or conduct, or reporting or dissemination plans of this research.

## Results

### Demographic and clinical characteristics

From January 1, 2020 and April 17, 2020, 233 pediatric COVID-19 cases were discharged from the 12 study hospitals (online supplementary Figure 1). These cases with a median age of 7.50 (IQR: 2.92,12.17) years ranged from 0.05 to 18.75 years old of which ~~and~~ 133 (57.1%) cases were male patients. Most patients (n = 199 [85.4%]) had a COVID-19-contact history, including contact with people from Wuhan/Hubei or travel to Wuhan/Hubei. 59 (26.5%) of the pediatric patients were the first SARS-CoV-2-infected individual of their family. Additionally, 40 (17.2%) patients had a simultaneous infection with an additional virus, and 79 (33.9%) patients had a simultaneous



For peer review only

For peer review only - http://bmjopen.bmj.com/site/about/guidelines.xhtml

bacterial infection. None of the pediatric patients in our study were found to be at risk of death due to COVID-19 during hospitalization (Table 1).

**Table 1. Clinical Features of 233 Pediatric Patients Infected with SARS-CoV-2**

| | | All patients N (%) | Asymptomatic N (%) | Mild[c] N (%) | Moderate Severe N (%) | p[e] |
|---|---|---|---|---|---|---|
| Clinical factors | | 233(100.0) | 42(18.0) | 162(69.5) | 29(12.4) | |
| Sex | Male | 133(57.1) | 29(69.0) | 88(54.3) | 16(55.2) | 0.223 |
| | Female | 100(42.9) | 13(31.0) | 74(45.7) | 13(44.8) | |
| Age, y | ≤ 5 | 84(36.1) | 6(14.3) | 61(37.7) | 17(58.6) | <0.001 |
| | > 5 | 149(63.9) | 36(85.7) | 101(62.3) | 12(41.4) | |
| Age, median (IQR), y | | 7.50(2.92,12.17) | 9.37(7.00,12.15) | 7.92(2.85,12.29) | 4.00(0.85,8.92) | 0.010[f] |
| Contact history of epidemic area | Yes | 199(85.4) | 41(97.6) | 132(81.5) | 26(89.7) | 0.015[g] |
| Number of infected family member | 0 | 59(26.5) | 4(9.8) | 46(29.5) | 9(34.6) | 0.185 |
| | 1 | 67(30.0) | 16(39.0) | 43(27.6) | 8(30.8) | |
| | 2 | 54(24.2) | 10(24.4) | 39(25.0) | 5(19.2) | |
| | ≥ 3 | 43(19.3) | 11(26.8) | 28(17.9) | 4(15.4) | |
| Combined with virus infection[a] | Yes | 40(17.2) | 2(4.8) | 31(19.1) | 7(24.1) | 0.039 |
| Combined with bacterial infection[b] | Yes | 79(33.9) | 13(31.0) | 60(37.0) | 6(20.7) | 0.142 |
| Fever | Yes | 86(36.9) | 0(0.0) | 69(42.6) | 17(58.6) | <0.001 |
| Cough | Yes | 83(35.6) | 0(0.0) | 68(42.0) | 15(51.7) | <0.001 |
| Running nose | Yes | 21(9.0) | 0(0.0) | 17(10.5) | 4(13.8) | 0.027[g] |
| Diarrhea | Yes | 12(5.2) | 0(0.0) | 9(5.6) | 3(10.3) | 0.127[g] |
| Nausea | Yes | 38(16.3) | 0(0.0) | 32(19.8) | 6(20.7) | 0.001[g] |
| Coughing of phlegm | Yes | 23(9.9) | 0(0.0) | 16(9.9) | 7(24.1) | 0.002[g] |
| Pharyngitis | Yes | 13(5.6) | 0(0.0) | 10(6.2) | 3(10.3) | 0.111[g] |
| X-ray or CT test findings | Abnormal | 139(59.7) | 17(40.5) | 94(58.0) | 28(96.6) | <0.001 |
| Complications | Yes | 31(13.3) | 3(7.1) | 22(13.6) | 6(20.7) | 0.268 |
| Days in hospital, median (IQR), d | | 12.00(9.00,17.00) | 10.50(8.25,14.00) | 12.00(9.00,17.00) | 13.00(9.75,17.00) | 0.416[f] |

a: Adenovirus, influenza virus, parainfluenza virus, respiratory syncytial virus, *etc*.

b: Legionella pneumophila, Mycoplasma pneumoniae, Chlamydia pneumoniae, *etc*.

c: Mild disease: upper respiratory symptoms for a short duration or asymptomatic infection; positive RT-PCR test for SARS-CoV-2; no abnormal radiographic and septic presentation.

d: Moderate disease: mild pneumonia; symptoms such as fever, cough, fatigue, headache, and myalgia; no complications and manifestations related to severe conditions.

e: p-values indicate the difference among four different disease types in pediatric COVID-19 patients with pneumonia.

f: p-value was obtained from a Kruskal-Wallis test; [g]: p-values were obtained from Fisher exact tests.

For peer review only



For peer review only - http://bmjopen.bmj.com/site/about/guidelines.xhtml

The most common clinical symptoms of the 233 pediatric COVID-19 patients were fever (40.8%), cough (35.6%) and nausea (16.3%). The symptoms of running nose, diarrhea, and pharyngitis were also reported. All patients underwent a chest X-ray or CT test, and the results suggest that 139 (59.7%) patients had lung abnormalities, which include 17(40.5%) of asymptomatic patients, 94 (58.0%) of mild patients, and 28 (96.6%) of moderate or severe patients. The lung abnormalities included ground-glass opacities and patchy shadows or multiple plaques in some of the lung lobes on a CT scan. The median length of the patient hospital stay was 12 days (IQR: 9–17 days). An examination of patients' complications revealed that 31 of the 233 pediatric COVID-19 patients had complications (e.g., myocardial injury and liver insufficiency, etc.), including 3 (7.1%) asymptomatic patients, 22 (13.6%) mild patients, and 6 (20.7%) moderate or severe patients.

The clinical disease was classified as asymptomatic in 42 (18.0%) patients, mild in 162 (69.5%) patients, moderate in 25 (10.7%) patients and severe in 4 (1.7%) patients. As the severity of disease increased, the median age ($p = 0.010$) decreased and the proportion of children under 5 years old ($p < 0.001$) increased. Notably, there was no significant sex difference in severity of disease ($p = 0.223$). When comparing between children with or without infected family members, the severity of illness exhibited no significant correlation. Among the patients with moderate or severe disease, 17 (58.6%) had fever, 15 (51.7%) had cough and 7 (24.1%) had coughing of phlegm. Only 13.8% and 10.3% had the symptoms of running nose and diarrhea, respectively. Of the mild cases, 69 (42.6%), 68 (42.0%), and 32 (19.8%) patients had fever, cough, and nausea, respectively. In contrast, none of the asymptomatic patients had any of the clinical symptoms that were common in the patients with mild or moderate disease.

## Laboratory indices at hospital admission for the 233 pediatric COVID-19 patients

Here are the results of laboratory indices obtained in the first laboratory test after hospital admission (Table 2). Of



For peer review only - http://bmjopen.bmj.com/site/about/guidelines.xhtml

the 229 cases, fifty had leukopenia. More than half of the patients (n = 150 [65.50%]) had an abnormal lymphocyte percentage; 52 (22.70%) had lymphopenia. In addition, some patients were diagnosed with abnormal liver function, as indicated by 69 (30.70%) patients with elevated AST levels, while 20 (8.80%) patients had elevated ALT levels and 41 (20.20%) had increased procalcitonin levels. We also detected renal damage in some patients, as indicated by elevated levels of creatinine (n = 12 [5.40%]) and urea (n = 11 [5.00%]). Some patients exhibited elevated levels of LDH (n = 36 [18.30%]) and α-HBDH (n = 13 [59.10%]). Levels of infection-related indices, such as IL-6 (35 [24.10%]), CRP (40 [19.00%]), and ESR (19 [17.00%]), were also elevated in some patients.

For peer review only



For peer review only - http://bmjopen.bmj.com/site/about/guidelines.xhtml

**Table 2. Laboratory Indices at Hospital Admission of Pediatric Patients with COVID-19 Pneumonia**

| Tests in study population | Reference values | No. of patients tested | Median (IQR[l]) | No. of patients with value deviation from reference (%) | |
|---|---|---|---|---|---|
| | | | | Below reference | Above reference |
| **Hematologic** | | | | | |
| White blood cells, $\times 10^9$/mL | Varied with age[j] | 229 | 6.64(5.26-8.18) | 50 (22.0) | 17 (7.4) |
| Lymphocytes, % | Varied with age[j] | 229 | 42.40(33.00-55.10) | 52 (22.7) | 98 (42.8) |
| Platelets, $\times 10^9$/L | 100-400 | 224 | 266.00(225.00-325.00) | 1 (0.4) | 20 (8.9) |
| Hemoglobin, g/L | Varied with age[j] | 224 | 129.00(119.80-138.20) | 16 (7.2) | 7 (3.2) |
| **Biochemical** | | | | | |
| AST[a], IU/L | 13-35 | 225 | 29.00(20.00-41.00) | 10 (4.4) | 69 (30.7) |
| ALT[b], IU/L | 7-40 | 227 | 15.50(11.00-24.75) | 16 (7.0) | 20 (8.8) |
| Albumin, g/L | 40-55 | 223 | 45.60(43.10-48.05) | 16 (7.2) | 3 (1.3) |
| Prealbumin, mg/L | 200-400 | 142 | 194.30(153.85-229.68) | 76 (53.5) | - |
| Urea, mmol/L | 2.8-7.6 | 218 | 3.97(3.23-5.06) | 28 (12.8) | 11 (5.0) |
| Creatinine, μmol/L | Varied with age[j] and sex | 225 | 36.60(26.80-48.90) | 8 (3.6) | 12 (5.4) |
| Cholinesterase, U/L | 5300-11300 | 40 | 8914.00(7690.00-10022.00) | - | 4 (10.0) |
| LDH[c], U/L | Varied with age[j] | 196 | 238.00(194.80-311.80) | 2 (1.0) | 36 (18.3) |
| α-HBDH[d], U/L | 72-182 | 22 | 185.50(162.50-231.50) | - | 13 (59.1) |
| Procalcitonin, ng/ml | < 0.1 | 186 | 0.05(0.03-0.08) | - | 41 (20.2) |
| **Infection-related indices** | | | | | |
| IL-6[e], pg/ml | < 7 | 145 | 3.63(2.36-6.85) | - | 35 (24.1) |
| CRP[f], mg/L | < 8 | 211 | - | - | 40 (19.0) |
| ESR[g], mm/h | Varied with age and sex[k] | 112 | 10.00(5.75-19.00) | - | 19 (17.0) |
| **Coagulation function** | | | | | |
| PT[h], s | 11-14.5 | 191 | 11.40(10.80-12.50) | 59 (30.9) | 6 (3.1) |
| APTT[i], s | 26-40 | 191 | 32.8(29.70-35.35) | 12 (6.3) | 25 (13.1) |
| D-dimer, ug/ml | 0-0.5 | 150 | 0.26(0.15-0.42) | - | 28 (18.7) |
| Serum troponin, ug/L | < 0.01 | 55 | - | - | 18 (30.0) |

a: aspartate aminotransferase; b: alanine aminotransferase; c: lactate dehydrogenase; d: α-hydroxybutyric dehydrogenase; e: interleukin-6; f: C-reactive protein; g: erythrocyte sedimentation rate; h: prothrombin time; i: activated partial thromboplastin time; l: interquartile range.

j: White blood cells ($\times 10^9$ cells/L): <28 days old: 10.0–24.0, 29 days–3 years old, 8.0–12.0, >3 years old: 4.0–10.0; Lymphocytes (%): <28 days old: 30–40, 29 days–3 years old: 50–70, >3 years old: 30–40; Hemoglobin ($\times 10^9$/L): <28 days old: 10.0–24.0, 29 days–3 years: 8.0–12.0, >3 years old: 4.0–10.0; LDH: 0–29 days old: 290–2000, 30 days–23 months old: 180–430, >23 months old: 110–290.

k: Creatinine (μmol/L): ≤2 months old: 22–90, 2 months–3 years old: 11–34, 3–15 years old: 21–65, >15 years old:



For peer review only - http://bmjopen.bmj.com/site/about/guidelines.xhtml

For peer review only

male: 64–104, female: 49–90; ESR (mm/h): male: 0–21, female: 0–26.

Figure 1 shows the difference between the results of the first and last laboratory tests for the 233 pediatric COVID-19 cases. The value of liver damage indices (AST [difference: 6.05 IU/L; 95%CI: 1.96–10.14 IU/L; p = 0.004]) and myocardial indices (LDH [difference, 40.33 IU/L; 95%CI, 5.11– 75.55 IU/L; p = 0.026]) were significantly decreased over the disease course. Additionally, the lymphocyte percentage (difference, -2.82%; 95%CI, -4.79% to −0.85%; $p < 0.006$), platelets count (difference, $-38.74\times10^9$ cells/L; 95%CI, −53.46 to $-24.02\times10^9$ cells/L; $p < 0.0001$), and prealbumin levels (difference: −25.15 mg/L; 95%CI: −39.41 to –10.89 mg/L; p = 0.001) were significantly increased by the final laboratory test. In order to remove age effects, the study examined the trends of different clinical indicators before and after treatment by age stratification (online supplementary Figure 2).

## Factors impacting the duration to return negative RT-PCR

Figure 2 shows the distribution in the number of days to return negative (i.e., the number of days from positive RT-PCR to negative RT-PCR results for SARS-CoV-2) in the 233 pediatric COVID-19 cases based on different classification criteria, including age, sex, and clinical type. This length of duration time from positive RT-PCR to negative for individual patients ranged from 0 to 32 days. The mean lengths of time to return negative RT-PCR for asymptomatic, mild, moderate, and severe cases were 7.86±4.26, 8.24±4.77, 8.56±3.18, and 15.25±9.57 days, respectively. Regarding patient age, the mean number of days to return negative was 7.73±7.73 and 8.66±4.76 for the groups aged less than five years old and more than five years old, respectively. Regarding patient sex, the mean length of time to return negative was 8.43±4.51 and 8.19±4.97 days in male and female patients, respectively.

We constructed Cox proportional hazards regression models to explore the factors that influenced the time to return negative. The results are shown in Table 3. After controlling for the factors of sex, age, and weight,



For peer review only - http://bmjopen.bmj.com/site/about/guidelines.xhtml

comparing the HRs for the time from positive RT-PCR to negative RT-PCR between patients who had infected family members and those who did not have infected family members yielded an HR and corresponding 95% confidence interval of 0.56 (0.41–0.79). Furthermore, comparisons of patients who had zero infected family members with those who had one, two, or at least three infected family members yielded HRs (95%CI) of 0.47 (0.32–0.69), 0.51 (0.34–0.76), and 0.56 (0.36–0.86), respectively. And a lower HR means a longer time to return negative. Regarding the clinical disease types, we found the group with severe disease had a significantly longer time to return negative RT-PCR (HR [95%CI]: 0.14 [0.03–0.59]), after controlling for demographic factors (p = 0.008), while the time from positive RT-PCR to negative of mild and moderate patients was not significantly different from that of asymptomatic patients. The symptom of the occurrence of emesis was significantly associated with the time to return negative (HR [95%CI]: 0.33 [0.14–0.78]), as was having complications (HR [95%CI]: 0.57 [0.37–0.89]). There was no significant association between use of traditional Chinese medicine (e.g, Forsythia, Honeysuckle, Isatis root) and antiviral drug and time to return negative (HR [95% CI]: 0.85 [0.64–1.13]).

For peer review only

**Table 3. Hazard Ratios of Time to Return Negative RT-PCR from COVID-19 in Cox Proportional Hazards Regression Model.**

| Risk factors | Univariate Cox regression[a] | | Multivariate Cox regression[a, b] | |
|---|---|---|---|---|
| | HR (95% CI) | *p* | HR (95% CI) | *p* |
| **Clinical characteristics** | | | | |
| Sex (versus male) | 1.10 (0.81-1.37) | 0.702 | - | - |
| Age | 0.82 (0.63-1.08) | 0.160 | - | - |
| Contact history | 1.40 (0.96-2.02) | 0.079 | 1.02(0.67-1.57) | 0.918 |
| **Clinical symptoms** | | | | |
| Fever | 0.94 (0.72-1.23) | 0.655 | 0.96(0.72-1.28) | 0.789 |
| Cold | 1.10 (0.27-4.45) | 0.890 | 1.15(0.28-4.67) | 0.844 |
| Diarrhea | 1.70 (0.97-3.13) | 0.063 | 1.56(0.85-2.90) | 0.155 |
| Emesis | 0.51 (0.24-1.11) | 0.089 | 0.33(0.14-0.78) | 0.012 |
| Nausea | 0.85 (0.60-1.20) | 0.348 | 0.76(0.53-1.10) | 0.147 |
| Running nose | 0.98 (0.63-1.54) | 0.934 | 0.94(0.58-1.53) | 0.814 |
| Coughing of phlegm | 0.94 (0.61-1.45) | 0.778 | 0.90(0.57-1.43) | 0.658 |
| Complications | 0.74 (0.50-1.08) | 0.12 | 0.57(0.37-0.89) | 0.013 |
| **Clinical type (versus asymptomatic)** | | | | |
| Mild | 0.91(0.65-1.28) | 0.596 | 0.92(0.65-1.31) | 0.663 |
| Moderate | 0.92(0.56-1.51) | 0.743 | 0.86(0.50-1.48) | 0.591 |
| Severe | 0.29(0.10-0.86) | 0.025 | 0.14(0.03-0.59) | 0.008 |
| **Household contact** | | | | |
| Family member infection | 0.65(0.48-0.89) | 0.006 | 0.56(0.41-0.79) | 0.001 |
| **Number of infectious family member** | | | | |
| 1 | 0.56(0.39-0.80) | 0.002 | 0.47(0.32-0.69) | <0.001 |
| 2 | 0.62(0.43-0.90) | 0.012 | 0.51(0.34-0.76) | 0.001 |
| ≥3 | 0.61(0.41-0.92) | 0.019 | 0.56(0.36-0.86) | 0.008 |
| **Other respiratory pathogens infection** | | | | |
| Combined with virus[c] infection | 0.92 (0.65-1.30) | 0.621 | 0.84(0.59-1.21) | 0.348 |
| Combined with bacterial infection[d] | 1.10 (0.82-1.44) | 0.574 | 1.04(0.78-1.40) | 0.771 |
| **Treatment (versus ATD)** | | | | |
| TCM[e]+ATD[f] | 0.80 (0.61-1.04) | 0.093 | 0.85(0.64-1.13) | 0.259 |

a: Results of Cox proportional hazards regression models are represented as hazard ratios and 95% confidence intervals. Wald tests were performed to calculate the *p*-values for trend.

b: Adjusted for sex, age, and weight.

c: Other detected viruses included adenovirus, influenza virus, parainfluenza virus, and respiratory syncytial virus.

d: Detected bacteria included Legionella pneumophila, Mycoplasma pneumoniae, and Chlamydia pneumoniae.

e: Traditional Chinese Medicine; f: Antiviral Drug



For peer review only

# Discussion

We found that nearly 70.4% of the pediatric COVID-19 cases were infected through close contact with family members. The pediatric COVID-19 patients in our cohort were diagnosed with mainly mild disease, followed by asymptomatic and moderate disease. The proportion of severe patients was lower compared with that generally observed in adult patients, a pattern which is consistent with other studies[18 19]. The median length of hospital stay in our pediatric COVID-19 patients was 12 days, which is similar to that reported for a pediatric COVID-19 case study in Wuhan[20]. Pediatric COVID-19 patients generally exhibited the clinical symptoms of fever, cough, nausea, and coughing of phlegm, which was consistent with other studies[21]. It has been reported that adult patients with gastrointestinal symptoms are more likely to develop severe conditions[22] which is consistent with the finding in pediatric patients. Notably, the pediatric COVID-19 cases who exhibited emesis symptom took a longer time to be clear from detectable SARS-CoV-2 (HR [95%CI]: 0.33 [0.14–0.78]).

In contrast with a similar study[10], we did find 17 asymptomatic pediatric COVID-19 patients who had abnormal X-ray or CT test results. Although the asymptomatic pediatric COVID-19 patients did not present the typical symptoms of pneumonia, the radiographic examinations were still able to provide some supportive evidence for diagnosis. Since most pediatric COVID-19 patients have mild type disease, routine CT examination is necessary to detect it because a plain X-ray examination may fail to show some lesion details, leading to a misdiagnosis or missed diagnosis[23]. We observed a tendency toward a higher detection of abnormalities in the lungs by X-ray or CT with increasingly more severe disease, evidenced by the detection of abnormalities in radiographic examinations for all the patients with moderate or severe type disease in our study ($p = <0.001$). Additionally, younger pediatric COVID-19 patients were found to be more prone to developing severe conditions ($p < 0.001$). Therefore, if they become infected with SARS-CoV-2, they would be more prone to developing a more severe condition with a more fragile immune system.



For peer review only - http://bmjopen.bmj.com/site/about/guidelines.xhtml

A small proportion of pediatric COVID-19 patients were found to have complications, although the incidence of complications in children with COVID-19 was lower than that observed in adults. Pediatric COVID-19 patients with underlying medical conditions were detected to have some abnormal clinical indices (e.g., elevated AST and procalcitonin levels and longer prothrombin time), and they took longer to return negative RT-PCR than did those without any complications (HR [95%CI]: 0.57 [0.37–0.89]). We suspected that pediatric COVID-19 patients whose physical functionality may be impaired before infection with SARS-CoV-2, e.g., by an underlying infection or other complications, will need more time to return negative from COVID-19.

Elevated levels of inflammatory factors such as IL-6 and CRP were observed in the pediatric COVID-19 patients. The reason for this finding could be that cytokine storm plays a role in the immune pathophysiology of COVID-19[23]. Similar immune mechanisms have been reported in previous influenza studies[24]. However, it would need more data to determine if cytokine storms are a major determinant of severity and probably of fatal outcome. Furthermore, some pediatric COVID-19 patients presented with abnormal coagulation function (elevated d-dimer and APTT levels). In contrast with the findings from a study of adult COVID-19 patients[25], the pediatric COVID-19 patients with these abnormalities had mostly mild or asymptomatic type disease, and there was no obvious evidence linking abnormal coagulation function with poor prognosis. Self-controlled studies of pediatric COVID-19 patients found significant increases in lymphocyte levels after treatment, suggesting that lymphocyte numbers are reduced in pediatric COVID-19 cases[10]. It has been suggested that this virus might directly infect lymphocytes, especially T lymphocytes, and initiate or promote lymphocyte cell death, resulting in lymphocyte depletion and a reduced antiviral response[26].

The Cox regression model indicated that the time from positive RT-PCR to negative is strongly influenced by the presence of SARS-CoV-2-infected individuals in their family. This finding suggests that the main transmission



For peer review only - http://bmjopen.bmj.com/site/about/guidelines.xhtml

route for COVID-19 in children is close contact with family members, which is consistent with findings from another research[27]. Therefore, social distancing and hygiene measures should be exercised in an ~~more~~ intimate transmission scenario (e.g., family settings[28]) containing a risk of higher viral load and exposure, which is also supported by the conclusions of Bielecki et al.[29] and Dalton CB et al.[30] that non-pharmaceutical measures can help reduce the probability of infection or prevent developing to more severe disease by quantitatively reduce the viral inoculum. We found 59 pediatric COVID-19 cases who were the first SARS-CoV-2-infected individuals in their families, which indicated that pediatric COVID-19 patients could be a primary source of infection, a potential causation pathway often ignored[8 31]. Due to the disease severity of pediatric patients are mainly mild and asymptomatic, it might be more difficult to detect such cases in the early stage of disease. In addition, we found that children with symptoms of emesis, the vast majority of age are below 5 years old which is a very fragile and vulnerable stage of life. On the one hand, these patients need longer time to return negative RT-PCR may be due to that they are weaker to clear the virus and their immunity system is still immature. On the other hand, the literature has also shown that the occurrence of virus infection can affect gastrointestinal function and cline to gastrointestinal symptoms[32-34] (e.g., emesis[19]).

This study had some limitations. Firstly, we failed to capture all asymptomatic patients as the nature of a hospital-based study. However, this failure had limited impact on the interpretation of our study because it mainly caused an underestimation of the true prevalence of asymptomatic patients which is beyond the objective of our research. Secondly, to study as large of a cohort as possible, we enrolled discharged pediatric COVID-19 patients from multiple hospitals, which can be very heterogeneous in terms of treatment and management characteristics. Possible limits due to the aspects were the use of traditional Chinese medicine and international normalize ration of PT which made the data hardly comparable with those of other nations; and caution should be also exercised in extrapolating these results to other countries, as children in China may have different baseline health conditions

For peer review only



For peer review only - http://bmjopen.bmj.com/site/about/guidelines.xhtml

and medical care access. Additionally, given this is a hospital-based study, we did not have accurate date if a patient was diagnosed before admission to hospital, the starting point of positive diagnosis was therefore defined by the data of admission. Caution should be exercised in explanation of these results.

## Conclusion

In conclusion, the pediatric cases with emesis symptom, complications or with infected family members will extend the length of duration from positive RT-PCR to negative. We suspected that the infecting viral load influences disease severity that emphasized on the importance of social distancing and interventions such as masks. It's difficult to detect asymptomatic pediatric COVID-19 patients; therefore, pose a potential risk to the community.

## Footnotes

**Contributors:** Weibing Wang, Guoying Huang and Zhiping Li conceptualized and designed the study, completed the literature search, critical revised the manuscript for important intellectual content. Hua Xu, Xianfeng Wang, Jinmiao Lu, Yidie Huang, Guangfei Wang, Yanling He, Jie Chen designed the data collection instruments, collected data and are responsible for the integrity of the data, reviewed and revised the manuscript. Shuli Ma, Shangrong Zou, Yuxia Cui, Ruijie Chen, Jin Lu, Xuyuan Li coordinated and supervised data collection, conducted the initial statistical analysis of epidemiology data, reviewed and revised the manuscript. Jianbo Shao, Hong Xu, Zhixi Liu, Xiaohua Ying constructed the Cox proportional hazards regression models and conducted the further statistical analysis of the study, and drafted the manuscript.

All authors approved the final manuscript as submitted to *BMJ Open* with the signature of the corresponding author and agree to be accountable for all aspects of the work.

**Funding:** This study was funded by the Bill & Melinda Gates Foundation [grant number INV-006277]. The funder



For peer review only - http://bmjopen.bmj.com/site/about/guidelines.xhtml

had no role in the study design, data collection, data analysis, data interpretation, or writing of the report. The corresponding author had full access to all data in the study and had final responsibility for the decision to submit for publication.

**Competing interests:** Jianbo Shao, Hong Xu, Zhixi Liu, Xiaohua Ying, Hua Xu, Xianfeng Wang, Jinmiao Lu, Yidie Huang, Guangfei Wang, Yanling He, Jie Chen, Shuli Ma, Shangrong Zou, Yuxia Cui, Ruijie Chen, Jin Lu, Xuyuan Li, Zhiping Li, Guoying Huang and Weibing Wang declare that they have no conflict of interest.

**Acknowledgments:** This study was sponsored by the Bill & Melinda Gates Foundation [grant number INV-006277]. We would like to extend my sincere thanks to Professor Stuart MacLeod (University of British Columbia) for his insightful comments. We thank Katie Oakley, PhD, from Liwen Bianji, Edanz Editing China (www.liwenbianji.cn/ac), for editing the English text of a draft of this manuscript.

**Data availability statement:** The data that support the findings of this study are available from the corresponding author, Weibing Wang, upon reasonable request.

**Ethics approval**

This study was approved by the ethics committee of the National Children's Medical Center, Children's Hospital of Fudan University [No.(2020) 187], and followed the Declaration of Helsinki. Written informed consents were obtained from the guardians of the patients prior to conducting the study.

For peer review only



For peer review only - http://bmjopen.bmj.com/site/about/guidelines.xhtml

# Reference


1. WHO. WHO Coronavirus (COVID-19) Dashboard. https://covid19.who.int/ (accessed August 12, 2021).
2. WHO. PANDEMIC https://www.who.int/news-room/detail/08-04-2020-who-timeline---covid-19(accessed May 10, 2020).
3. WHO. Fact sheet of Pneumonia. https://www.who.int/en/news-room/fact-sheets/detail/pneumonia(accessed May 10, 2020).
4. NHC P. Report on the development of maternal and child health in China (2019). http://www.nhc.gov.cn/fys/s7901/201905/bbd8e2134a7e47958c5c9ef032e1dfa2.shtml(accessed May 10, 2020; in Chinese).
5. WHO. Consensus document on the epidemiology of severe acute respiratory syndrome (SARS).2003.
6. Hon KLE, Leung CW, Cheng WTF, et al. Clinical presentations and outcome of severe acute respiratory syndrome in children. *The Lancet* 2003;361(9370):1701-03. doi: 10.1016/s0140-6736(03)13364-8
7. Peiris JSM, Lai ST, Poon LLM, et al. Coronavirus as a possible cause of severe acute respiratory syndrome. *The Lancet* 2003;361(9366):1319-25. doi: 10.1016/s0140-6736(03)13077-2
8. Peiris JS, Yuen KY, Osterhaus AD, et al. The severe acute respiratory syndrome. *N Engl J Med* 2003;349(25):2431-41. doi: 10.1056/NEJMra032498 [published Online First: 2003/12/19]
9. Assiri A, Al-Tawfiq JA, Al-Rabeeah AA, et al. Epidemiological, demographic, and clinical characteristics of 47 cases of Middle East respiratory syndrome coronavirus disease from Saudi Arabia: a descriptive study. *Lancet Infect Dis* 2013;13(9):752-61. doi: 10.1016/s1473-3099(13)70204-4 [published Online First: 2013/07/31]
10. Qiu H, Wu J, Hong L, et al. Clinical and epidemiological features of 36 children with coronavirus disease 2019 (COVID-19) in Zhejiang, China: an observational cohort study. *Lancet Infect Dis* 2020 doi: 10.1016/s1473-3099(20)30198-5 [published Online First: 2020/03/30]
11. NHC P. Diagnosis and treatment guidelines for 2019 novel coronavirus pneumonia (draft version 5). http://www.nhc.gov.cn/jkj/s3577/202002/a5d6f7b8c48c451c87dba14889b30147/files/3514cb996ae24e2faf65953b4ecd0df4.pdf (access May 20, 2020; in Chinese).
12. Thygesen K, Alpert JS, Jaffe AS, et al. Fourth Universal Definition of Myocardial Infarction (2018). *Circulation* 2018;138(20):e618-e51. doi: 10.1161/CIR.0000000000000617 [published Online First: 2018/12/21]
13. MedGen. Hepatic insufficiency. https://www.ncbi.nlm.nih.gov/medgen/227013 (accessed August 1, 2021). *National Center for Biotechnology information*
14. WHO. Clinical management of severe acute respiratory infection when novel coronavirus (nCoV) infection is suspected: interim guidance. January 28, 2020.(accessed May 10, 2020).
15. Chen Z, Fu J, Shu Q, et al. Diagnosis and treatment recommendation for pediatric coronavirus disease-19. *Zhejiang Da Xue Xue Bao Yi Xue Ban* 2020;49(1):1-8. [published Online First: 2020/02/18]
16. NHC P. National Health and Health Commission of the People's Republic of China. Diagnosis and Treatment Protocol for Novel Coronavirus Pneumonia (Trial Version 7).
17. Feng F, Dongchi Z, Yu C, et al. Diagnosis and prevention recommendations of Novel Coronavirus 2019 in peidatric patients. *Chinese Journal of Pediatrics* 2020(03):169-74. doi: 10.3760/cma.j.issn.0578-1310.2020.03.001
18. Davies NG, Klepac P, Liu Y, et al. Age-dependent effects in the transmission and control of COVID-19 epidemics. *Nat Med* 2020;26(8):1205-11. doi: 10.1038/s41591-020-0962-9 [published Online First: 2020/06/18]




For peer review only - http://bmjopen.bmj.com/site/about/guidelines.xhtml

19. Cui X, Zhao Z, Zhang T, et al. A systematic review and meta-analysis of children with coronavirus disease 2019 (COVID-19). *J Med Virol* 2021;93(2):1057-69. doi: 10.1002/jmv.26398 [published Online First: 2020/08/08]
20. Xia W, Shao J, Guo Y, et al. Clinical and CT features in pediatric patients with COVID-19 infection: Different points from adults. *Pediatr Pulmonol* 2020;55(5):1169-74. doi: 10.1002/ppul.24718 [published Online First: 2020/03/07]
21. Ng DC, Tan KK, Chin L, et al. Clinical and epidemiological characteristics of children with COVID-19 in Negeri Sembilan, Malaysia. *Int J Infect Dis* 2021;108:347-52. doi: 10.1016/j.ijid.2021.05.073 [published Online First: 2021/06/05]
22. Wan Y, Li J, Shen L, et al. Enteric involvement in hospitalised patients with COVID-19 outside Wuhan. *Lancet Gastroenterol Hepatol* 2020 doi: 10.1016/s2468-1253(20)30118-7 [published Online First: 2020/04/19]
23. Li H, Liu L, Zhang D, et al. SARS-CoV-2 and viral sepsis: observations and hypotheses. *The Lancet* 2020 doi: 10.1016/s0140-6736(20)30920-x
24. Iwasaki A, Pillai PS. Innate immunity to influenza virus infection. *Nat Rev Immunol* 2014;14(5):315-28. doi: 10.1038/nri3665 [published Online First: 2014/04/26]
25. Zhou F, Yu T, Du R, et al. Clinical course and risk factors for mortality of adult inpatients with COVID-19 in Wuhan, China: a retrospective cohort study. *Lancet* 2020;395(10229):1054-62. doi: 10.1016/s0140-6736(20)30566-3 [published Online First: 2020/03/15]
26. Hamming I, Timens W, Bulthuis ML, et al. Tissue distribution of ACE2 protein, the functional receptor for SARS coronavirus. A first step in understanding SARS pathogenesis. *J Pathol* 2004;203(2):631-7. doi: 10.1002/path.1570 [published Online First: 2004/05/14]
27. Su L, Ma X, Yu H, et al. The different clinical characteristics of corona virus disease cases between children and their families in China - the character of children with COVID-19. *Emerg Microbes Infect* 2020;9(1):707-13. doi: 10.1080/22221751.2020.1744483 [published Online First: 2020/03/27]
28. Kim J, Choe YJ, Lee J, et al. Role of children in household transmission of COVID-19. *Arch Dis Child* 2021;106(7):709-11. doi: 10.1136/archdischild-2020-319910 [published Online First: 2020/08/10]
29. Bielecki M, Zust R, Siegrist D, et al. Social distancing alters the clinical course of COVID-19 in young adults: A comparative cohort study. *Clin Infect Dis* 2020 doi: 10.1093/cid/ciaa889 [published Online First: 2020/07/01]
30. Dalton CB, Corbett SJ, Katelaris AL. COVID-19: implementing sustainable low cost physical distancing and enhanced hygiene. *Med J Aust* 2020;212(10):443-46 e1. doi: 10.5694/mja2.50602 [published Online First: 2020/05/02]
31. Jiehao C, Jin X, Daojiong L, et al. A Case Series of Children With 2019 Novel Coronavirus Infection: Clinical and Epidemiological Features. *Clin Infect Dis* 2020;71(6):1547-51. doi: 10.1093/cid/ciaa198 [published Online First: 2020/03/01]
32. Chiappini E, Licari A, Motisi MA, et al. Gastrointestinal involvement in children with SARS-COV-2 infection: An overview for the pediatrician. *Pediatr Allergy Immunol* 2020;31 Suppl 26:92-95. doi: 10.1111/pai.13373 [published Online First: 2020/11/26]
33. Parasa S, Desai M, Thoguluva Chandrasekar V, et al. Prevalence of Gastrointestinal Symptoms and Fecal Viral Shedding in Patients With Coronavirus Disease 2019: A Systematic Review and Meta-analysis. *JAMA Netw Open* 2020;3(6):e2011335. doi: 10.1001/jamanetworkopen.2020.11335 [published Online First: 2020/06/12]
34. Andrews PLR, Cai W, Rudd JA, et al. COVID-19, nausea, and vomiting. *J Gastroenterol Hepatol*




For peer review only - http://bmjopen.bmj.com/site/about/guidelines.xhtml

2021;36(3):646-56. doi: 10.1111/jgh.15261 [published Online First: 2020/09/22]

## Figure Legend:

**Figure 1 Comparing the clinical indicators of pediatric COVID-19 patients pre-therapy and post-treatment during hospitalization**

The effect of treatment in 233 pediatric COVID-19 patients. The red and green boxes represent the values of five clinical indicators before and after treatment, respectively. The scattered points on each box represent the values of each clinical indicator for each patient. The middle line of the box represents the medium value of the group. The height of box represents the interquartile range (IQR) of each group. The *p*-value is calculated based on the paired Student's *t*-test.

* represents a *p*-value of less than 0.05; ** represents a *p*-value of less than 0.005; *** represents a *p*-value of less than 0.001.

**Figure 2. Distribution of the time to return negative of the 233 pediatric COVID-19 patients**

The distribution of the mean time to return negative RT-PCR for the 233 pediatric COVID-19 patients during the period of admission, split in groups based on different classification criteria. The gradient blue bars represent the mean number of days to return negative at age ≤5 years and age >5 years. The purple and pink bars represent mean number of days to return negative for male and female patients, respectively. The four bars on the right represent the mean number of days to return negative with the four clinical types of COVID-19. The scattered points on each bar represent the number of days to return negative for each patient in the group. The error lines represent the mean value plus or minus a standard deviation. Turning negative was defined as the time at which a confirmed COVID-19 patient first received negative results for an RT-PCR test for SARS-CoV-2.

For peer review only



For peer review only - http://bmjopen.bmj.com/site/about/guidelines.xhtml

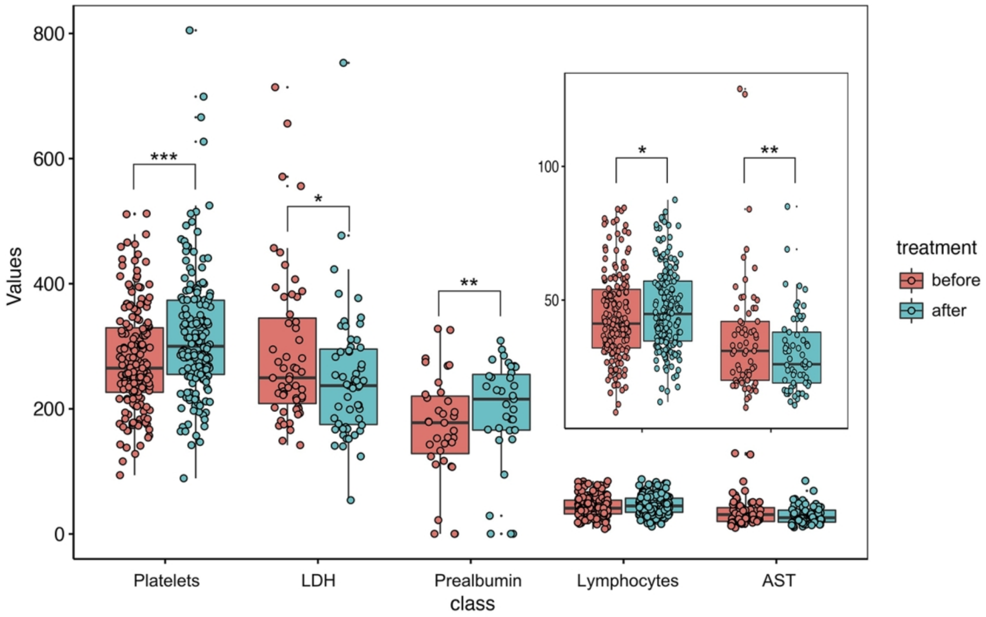


Figure 1 Comparing the clinical indicators of pediatric COVID-19 patients pre-therapy and post-treatment during hospitalization / The effect of treatment in 233 pediatric COVID-19 patients. The red and green boxes represent the values of five clinical indicators before and after treatment, respectively. The scattered points on each box represent the values of each clinical indicator for each patient. The middle line of the box represents the medium value of the group. The height of box represents the interquartile range (IQR) of each group. The p-value is calculated based on the paired Student's t-test.* represents a p-value of less than 0.05; ** represents a p-value of less than 0.005; *** represents a p-value of less than 0.001.

129x81mm (350 x 350 DPI)

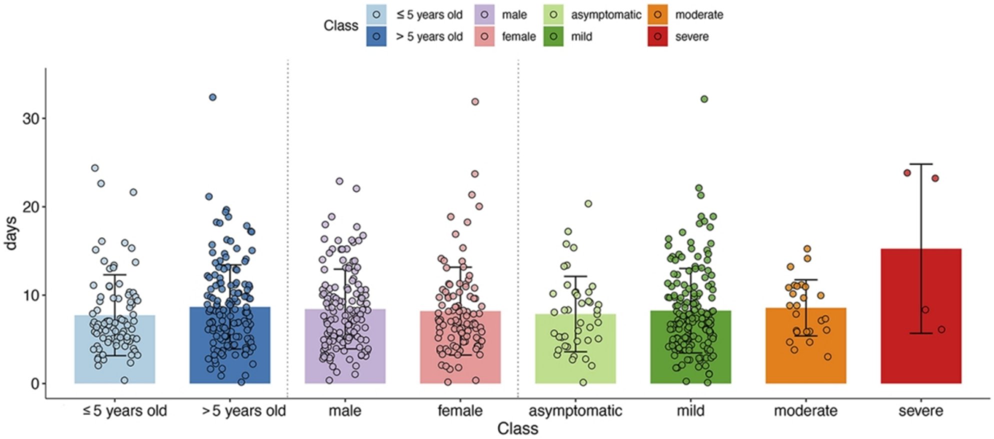


Figure 2. Distribution of the time to return negative of the 233 pediatric COVID-19 patients
The distribution of the mean time to return negative RT-PCR for the 233 pediatric COVID-19 patients during the period of admission, split in groups based on different classification criteria. The gradient blue bars represent the mean number of days to return negative at age ≤5 years and age >5 years. The purple and pink bars represent mean number of days to return negative for male and female patients, respectively. The four bars on the right represent the mean number of days to return negative with the four clinical types of COVID-19. The scattered points on each bar represent the number of days to return negative for each patient in the group. The error lines represent the mean value plus or minus a standard deviation. Turning negative was defined as the time at which a confirmed COVID-19 patient first received negative results for an RT-PCR test for SARS-CoV-2.

110x50mm (350 x 350 DPI)

# Title: Factors associated with the time to return negative RT-PCR from COVID-19 in pediatric patients: a retrospective cohort study

## Authors and Institutions:

Jianbo Shao[a, †], Hong Xu[b, †], Zhixi Liu[c, †], Xiaohua Ying[c, †], Hua Xu[d], Xianfeng Wang[e], Jinmiao Lu[f], Yidie Huang[f], Guangfei Wang[f], Yanling He[g], Jie Chen[h], Shuli Ma[i], Shangrong Zou[j], Yuxia Cui[k], Ruijie Chen[l], Jin Lu[m], Xuyuan Li[n], Zhiping Li[f, *], Guoying Huang [f, *], Weibing Wang [c, *]

[a]Department of Radiological Medicine, Wuhan Children's Hospital (Wuhan Maternal and Child Healthcare Hospital), Tongji Medical College, Huazhong University of Science & Technology, Wuhan 430016, China; and [b]Department of Nephrology, National Children's Medical Center, Children's Hospital of Fudan University, Shanghai 201102, China; and [c]School of Public Health, Fudan University, Shanghai 200032, China; and [d]Department of Pharmacy, Wuhan Children's Hospital (Wuhan Maternal and Child Healthcare Hospital), Tongji Medical College, Huazhong University of Science & Technology, Wuhan 430016, China; and [e]Department of Pediatrics, The Third People's Hospital of Shenzhen, Shenzhen 518020, China; and [f]Department of Clinical Pharmacy, National Children's Medical Center, Children's Hospital of Fudan University, Shanghai 201102, China; and [g]Department of Pharmacy, Guangzhou Women and Children's Medical Center, Guangzhou 510623, China; and [h]Department of Pharmacy, The Third People's Hospital of Kunming, Kunming 650000, China; and [i]Department of Pharmacy, Children's Hospital Affiliated to Zhengzhou University, Zhengzhou 450000, China; and [j]Department of Pharmacy, Guangzhou Eighth People's Hospital, Guangzhou 510060, China; and [k]Department of Pediatrics, Guizhou Provincial People's Hospital, Guiyang 550002, China; and [l]Wenzhou Municipal Key Laboratory of Paediatric Pharmacy, Department of Pharmacy, The Second

For peer review only

Affiliated Hospital and Yuying Children's Hospital of Wenzhou Medical University, Wenzhou 325027, China; and [m]Department of Pharmacy, The First Affiliated Hospital of USTC, Division of Life Sciences and Medicine, University of Science and Technology of China, Anhui 230001, China; and [n]Department of Biology, University of California, Santa Barbara 93101, California, the United States

[†] Jianbo Shao, Hong Xu, Zhixi Liu, Xiaohua Ying contributed equally to this manuscript

[*] Zhiping Li, Guoying Huang, Weibing Wang contributed equally to corresponding work of this manuscript

## Corresponding author:

Dr. Zhiping Li, PhD, Department of Clinical Pharmacy, National Children's Medical Center, Children's Hospital of Fudan University, Shanghai 201102, China

Email: zpli@fudan.edu.cn

Dr. Guoying Huang*, MD, PhD, Centre of Cardiovascular, National Children's Medical Center, Children's Hospital of Fudan University, Shanghai 201102, China

Email: gyhuang@shmu.edu.cn

Dr. Weibing Wang, PhD, Department of Epidemiology, School of Public Health, Fudan University, 138 Yi Xue Yuan Road, Shanghai 200032, China, 86-21-54237811

Email: wwb@fudan.edu.cn

## Contents:

Supplementary Table 1. Definitions of clinical types for COVID-19 pediatric patients
Supplementary Figure 1. The spatial distribution of the 12 COVID-19 tertiary hospitals
Supplementary Figure 2. Comparison of clinical indicators stratified by age pre-therapy and post-treatment

For peer review only

**Supplementary Table 1. Definitions of clinical types for COVID-19 pediatric patients**

| Classification of clinical types | Based on the clinical characteristics of confirmed COVID-19 pediatric cases |
|---|---|
| Asymptomatic disease | • No clinical symptoms or signs<br>• Normal chest imaging examination<br>• Positive RT-PCR test for SARS-CoV-2 or positive result for retrospectively diagnosed sero-specific antibody |
| Mild disease | • Acute upper respiratory tract infection (*e.g.*, fever, fatigue, myalgia, cough, sore throat, runny nose and sneezing, *etc.*)<br>• Pharyngeal congestion<br>• No positive signs of lungs<br>• Some may have no fever, or only other gastrointestinal symptoms (*e.g.*, nausea, vomiting, abdominal pain and diarrhea) |
| Moderate disease | • Pneumonia<br>• Clinical symptoms such as fever, cough<br>• Wheezing, dry and (or) moist rales were heard in the lungs<br>• Sub-clinical: some without clinical symptoms or signs but with abnormal pulmonary lesions |
| Severe disease | • Early symptoms of respiratory tract (*e.g.*, fever and cough, *etc*); may be accompanied by digestive tract symptoms (e.g., diarrhea, *etc*)<br>• Progression of disease within a week or so, with dyspnea, central cyanosis, hypoxia, pulse oximetry <0.92 |
| Critical disease | • ARDS<br>• Respiratory failure<br>• Multiple organ dysfunction (*e.g.*, shock, encephalopathy, myocardial injury or failure, coagulation dysfunction and acute kidney injury) |

For peer review only

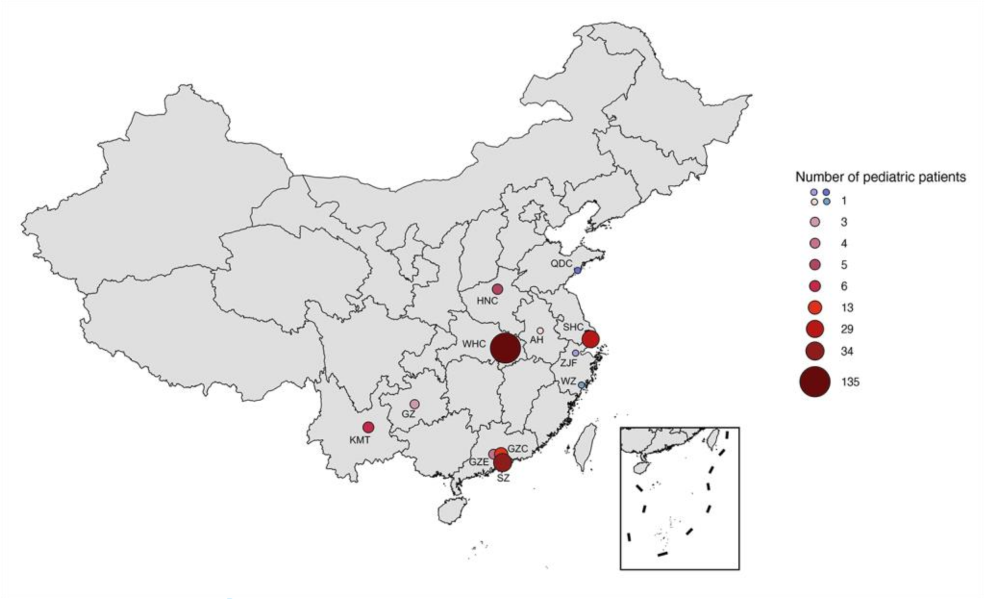


**Supplementary Figure 1. The spatial distribution of the 12 COVID-19 tertiary hospitals**

The number of patients enrolled in the study is varied by the size of the points and the hospitals varied by the color of the points. Abbreviations: WHC: Wuhan Children's Hospital (Wuhan Maternal and Child Healthcare Hospital), Tongji Medical College, Huazhong University of Science & Technology; SZ: The Third People’s Hospital of Shenzhen; SHC: National Children’s Medical Center, Children’s Hospital of Fudan University; GZC: Guangzhou Women and Children’s Medical Center; KMT: The Third People’s Hospital of Kunming; HNC: Children’s Hospital Affiliated to Zhengzhou University; GZE: Guangzhou Eighth People’s Hospital; GZ: Guizhou Provincial People’s Hospital; QDC: Qingdao Children’s Hospital; AH: The First Affiliated Hospital of USTC, Division of Life Sciences and Medicine, University of Science and Technology of China; ZJF: The First Hospital of Zhejiang University and WZ: The Second Affiliated Hospital and Yuying Children’s Hospital of Wenzhou Medical University.

For peer review only

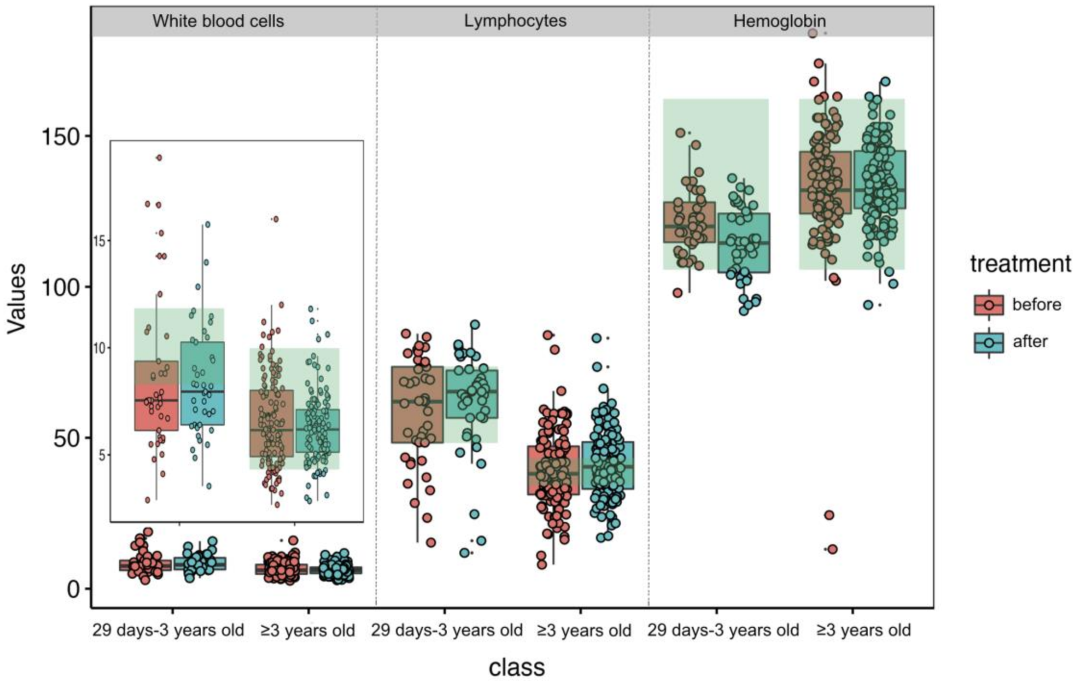


**Supplementary Figure 2. Comparison of clinical indicators stratified by age pre-therapy and post-treatment**

The red and green boxes represent the clinical indicator values before and after treatment, respectively. The scattered points on each box represent the values of each clinical indicator for each patient. The shaded green rectangle represents the normal reference range for an indicator for that age group. The middle line of the box represents the medium value of the group. The height of the box represents the interquartile range (IQR) of each group.

For peer review only

**STROBE 2007 (v4) Statement—Checklist of items that should be included in reports of *cohort studies***

| Section/Topic | Item # | Recommendation | Reported on page # |
|---|---|---|---|
| **Title and abstract** | 1 | (*a*) Indicate the study's design with a commonly used term in the title or the abstract | 1, 3 |
| | | (*b*) Provide in the abstract an informative and balanced summary of what was done and what was found | 3-5 |
| **Introduction** | | | 6 |
| Background/rationale | 2 | Explain the scientific background and rationale for the investigation being reported | 6 |
| Objectives | 3 | State specific objectives, including any prespecified hypotheses | 6 |
| **Methods** | | | 6-9 |
| Study design | 4 | Present key elements of study design early in the paper | 6-7 |
| Setting | 5 | Describe the setting, locations, and relevant dates, including periods of recruitment, exposure, follow-up, and data collection | 6-7 |
| Participants | 6 | (*a*) Give the eligibility criteria, and the sources and methods of selection of participants. Describe methods of follow-up | 6-7 |
| | | (*b*) For matched studies, give matching criteria and number of exposed and unexposed | Not Applicable |
| Variables | 7 | Clearly define all outcomes, exposures, predictors, potential confounders, and effect modifiers. Give diagnostic criteria, if applicable | 7-8 |
| Data sources/ measurement | 8* | For each variable of interest, give sources of data and details of methods of assessment (measurement). Describe comparability of assessment methods if there is more than one group | 7-8 |
| Bias | 9 | Describe any efforts to address potential sources of bias | Not Applicable |
| Study size | 10 | Explain how the study size was arrived at | 6-7 |
| Quantitative variables | 11 | Explain how quantitative variables were handled in the analyses. If applicable, describe which groupings were chosen and why | 8 |
| Statistical methods | 12 | (*a*) Describe all statistical methods, including those used to control for confounding | 8-9 |
| | | (*b*) Describe any methods used to examine subgroups and interactions | 8-9 |
| | | (*c*) Explain how missing data were addressed | 9 |
| | | (*d*) If applicable, explain how loss to follow-up was addressed | Not Applicable |
| | | (*e*) Describe any sensitivity analyses | Not Applicable |
| **Results** | | | 9-16 |

For peer review only

| | | | |
|---|---|---|---|
| Participants | 13* | (a) Report numbers of individuals at each stage of study—eg numbers potentially eligible, examined for eligibility, confirmed eligible, included in the study, completing follow-up, and analysed | 9-11 |
| | | (b) Give reasons for non-participation at each stage | Not Applicable |
| | | (c) Consider use of a flow diagram | Not Applicable |
| Descriptive data | 14* | (a) Give characteristics of study participants (eg demographic, clinical, social) and information on exposures and potential confounders | 9-11 |
| | | (b) Indicate number of participants with missing data for each variable of interest | 9-14 |
| | | (c) Summarise follow-up time (eg, average and total amount) | 9 |
| Outcome data | 15* | Report numbers of outcome events or summary measures over time | 9-16 |
| Main results | 16 | (*a*) Give unadjusted estimates and, if applicable, confounder-adjusted estimates and their precision (eg, 95% confidence interval). Make clear which confounders were adjusted for and why they were included | 9-16 |
| | | (*b*) Report category boundaries when continuous variables were categorized | 9-10 |
| | | (*c*) If relevant, consider translating estimates of relative risk into absolute risk for a meaningful time period | Not Applicable |
| Other analyses | 17 | Report other analyses done—eg analyses of subgroups and interactions, and sensitivity analyses | Not Applicable |
| **Discussion** | | | 17-20 |
| Key results | 18 | Summarise key results with reference to study objectives | 17 |
| **Limitations** | | | 20 |
| Interpretation | 20 | Give a cautious overall interpretation of results considering objectives, limitations, multiplicity of analyses, results from similar studies, and other relevant evidence | 20 |
| Generalisability | 21 | Discuss the generalisability (external validity) of the study results | 20 |
| **Other information** | | | 20-22 |
| Funding | 22 | Give the source of funding and the role of the funders for the present study and, if applicable, for the original study on which the present article is based | 21 |

*Give information separately for cases and controls in case-control studies and, if applicable, for exposed and unexposed groups in cohort and cross-sectional studies.

**Note:** An Explanation and Elaboration article discusses each checklist item and gives methodological background and published examples of transparent reporting. The STROBE checklist is best used in conjunction with this article (freely available on the Web sites of PLoS Medicine at http://www.plosmedicine.org/, Annals of Internal Medicine at http://www.annals.org/, and Epidemiology at http://www.epidem.com/). Information on the STROBE Initiative is available at www.strobe-statement.org.

For peer review only
